# Verteporfin‐Mediated In Situ Nanovaccine Based on Local Conventional‐Dose Hypofractionated Radiotherapy Enhances Antitumor and Immunomodulatory Effect

**DOI:** 10.1002/advs.202413387

**Published:** 2025-04-15

**Authors:** Zhifan Zhang, Lin Li, Yuchen Ge, Anni Chen, Shanchao Diao, Yueling Yang, Qianyue Chen, Yingling Zhou, Jie Shao, Fanyan Meng, Lixia Yu, Manman Tian, Xiaoping Qian, Zhaoyu Lin, Chen Xie, Baorui Liu, Rutian Li

**Affiliations:** ^1^ The Comprehensive Cancer Center of Nanjing Drum Tower Hospital Affiliated Hospital of Medical School Nanjing University Nanjing 210008 China; ^2^ Clinical Cancer Institute Nanjing University Nanjing 210008 China; ^3^ Department of Pathology Nanjing Drum Tower Hospital Affiliated Hospital of Medical School Nanjing University Nanjing 210008 China; ^4^ Department of Oncology Nanjing Drum Tower Hospital Clinical College of Traditional Chinese and Western Medicine Nanjing University of Chinese Medicine Nanjing 210008 China; ^5^ Nanjing International Hospital Medical School of Nanjing University Nanjing 210019 China; ^6^ State Key Laboratory for Organic Electronics and Information Displays & Institute of Advanced Materials (IAM) Jiangsu Key Laboratory for Biosensors Nanjing University of Posts & Telecommunications Nanjing 210023 China; ^7^ State Key Laboratory of Pharmaceutical Biotechnology Ministry of Education Key Laboratory of Model Animal for Disease Study Jiangsu Key Laboratory of Molecular Medicine Model Animal Research Center National Resource Center for Mutant Mice of China Nanjing Drum Tower Hospital School of Medicine Nanjing University Nanjing 210061 China

**Keywords:** hypofractionated radiotherapy, immunomodulatory effect, nanovaccination, radiosensitization, tumor‐targeting nanoparticles, verteporfin

## Abstract

In situ radiotherapy is the most successful cytotoxic therapy available for the treatment of solid tumors, while high‐dose radiotherapy per fraction is not yet widely and reliably used. To some extent, the major considerations of the disappointing results are on the risk of high‐dose irradiation‐induced damage to the surrounding normal tissues and the difficulty in distant metastasis control. To break these restraints, a gelatinase‐responsive amphiphilic methoxypolyethyleneglycol–PVGLIG–polycaprolactone (mPEG–PVGLIG–PCL) nanoparticles’ loading verteporfin (N@VP), a special photosensitizer that can also be excited by X‐rays to produce cytotoxic singlet oxygen and greatly enhance radiotherapy efficacy, is prepared in this study. Herein, it is shown that the formed N@VP combined with conventional‐dose radiation therapy (RT, 2 Gy (gray, a radiation dose unit)) can realize an antitumor effect no less than high‐dose RT (8 Gy) and minimize radiation dose necessary to achieve local tumor control. Moreover, this radiosensitive nanosystem can exert excellent systemic antitumor immunity and abscopal effect, providing a preferable “in situ vaccine” strategy based on conventional‐dose RT to achieve efficient systemic management of distant tumor metastasis. When combined with immunotherapy, this novel strategy for radiosensitization results in better immunotherapy sensitivity by stimulating significant immunogenic tumor cell death and synergistic antitumor immune responses.

## Graphical abstract

Here gelatinase‐responsive methoxypolyethyleneglycol–PVGLIG–polycaprolactone (mPEG–PVGLIG–PCL) nanoparticles’ loading verteporfin (N@VP) is developed, enhancing radiotherapy via X‐ray‐triggered singlet oxygen generation. Combined with 2 Gy (gray, a radiation dose unit) irradiation, N@VP achieves antitumor efficacy comparable to 8 Gy, and also promotes systemic antitumor immunity, abscopal effects, and synergizes with immunotherapy by inducing tumor cell pyroptosis, offering a more advantageous radiosensitization strategy for metastatic control based on conventional‐dose radiotherapy.

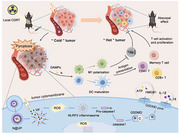

## 1 Introduction

Lung cancer is the second most frequently diagnosed cancer and the main cause of cancer death.^[^ ^1^ ^]^ Non‐small‐cell lung cancer (NSCLC), a heterogeneous group of tumors, accounts for about 85% of all new cases of lung cancer,^[^ ^2^ ^]^ which is characterized by high relapse rate and poor survival. In addition to immune checkpoint‐induced tumor immune suppression, the weak tumor immunogenicity and insufficient infiltration of tumor immune cells are more crucial mechanisms involving in immune escape, leading to ineffective immune checkpoint inhibitors’ (ICIs) treatment for NSCLC patients.^[^ ^3^ ^]^ Radiation therapy (RT) is a fundamental therapy of early‐ and advanced‐stage NSCLC, and radiation targeted to the tumor sites potentially enhances tumor immunogenicity by inducing sufficient DNA damage and release of tumor‐associated antigens (TAAs) during cell death and triggers more immune infiltration of tumor microenvironment (TME), thus activating powerful and tumor‐specific immunity synergistically with ICIs in NSCLC patients.^[^ ^4^ ^]^ Accumulating evidence indicated that radiation doses lower than 8–10 Gy (gray, a radiation dose unit) per fraction might propitiously generate sufficient cancer cell death to release antigens and trigger antigen‐specific adaptive immunity without aggravating hypoxia and immunosuppression.^[^ ^4^, ^5^ ^]^ Moreover, it is reported that hypofractionated radiotherapy administered to the tumor in three 8 Gy fractions (8 Gy × 3*f*) was similarly effective at controlling irradiated tumor growth, compared to a single 30 Gy fraction, while nonirradiated (abscopal) tumor growth suppression was only observed in mice treated with hypofractionated radiotherapy (8 Gy × 3*f*) instead of single dose (20 or 30 Gy) of irradiation when combined with ICIs.^[^ ^6^ ^]^ Although high‐dose radiotherapy (HDRT, >5 Gy) per fraction enhances tumor elimination and increases TAAs’ release compared with conventional‐dose radiotherapy (CDRT, 0.5–2 Gy), it is confined by inevitable radiation‐induced damage to the normal surrounding tissues especially when the radiation is delivered to the multifocal and small tumor sites.^[^ ^7^ ^]^ The standard treatment for early‐stage I and II NSCLC patients (lymph node negative) is stereotactic body radiation therapy (SBRT, high‐dose hypofractionated RT) to a biological equivalent dose of ≥100 Gy, while under this dosage of irradiation, the risk of irradiation‐induced heart injury, radiation esophagitis, radiation pneumonia and bronchial stenosis, or other serious clinical complications will be higher.^[^ ^8^ ^]^ Some tissues such as spinal cord may present asymptomatic subclinical damage and delayed responses to HDRT, making patients easily experience more serious side effects after reirradiation.^[^ ^9^ ^]^ Apart from the damage of surrounding tissues, tumor metastasis or difficulty in nonirradiated tumor control, which are the main causes for cancer‐related death, and HDRT‐induced suppressive immune responses are challenges worth addressing.^[^ ^10^ ^]^

To overcome these restrictions, we noticed an effective porphycene photosensitizer called verteporfin (benzoporphyrin derivative monoacid ring A, VP), which has been commonly used for clinical photodynamic therapy (PDT) of macular degeneration in the past decade years because it can be activated by laser light (689 nm), and produces singlet oxygen to occlude choroidal vessels with safe administration due to the fast elimination in the liver.^[^ ^11^ ^]^ The applications of PDT with verteporfin and laser light have also been reported in cancer treatment for its nonthermal cytotoxic effect mediated by reactive oxygen species (ROS), and a phase I/II study revealed that verteporfin (0.4 mg kg^−1^, equivalent to 5 mg kg^−1^ in mice)‐mediated invasive PDT was effective for locally advanced pancreatic cancer treatment with no treatment‐related long‐term side effects.^[^ ^12^ ^]^ However, the poor tissue penetration ability of laser light curbed the application of PDT for tumors in most cases located deep in the body. Currently, the PDT for deep‐seated tumors is only realizable through invasive delivery such as inserting optical fibers into tissues, while a few recent studies have proposed and confirmed that verteporfin, in addition to light excitation, can be activated by noninvasive techniques of X‐rays with superior tissue‐penetrating capability. Then, the activated verteporfin transfers energy from its electronically excited triplet state to nearby oxygen molecules to generate cytotoxic ROS, predominantly singlet oxygen (^1^O_2_), which would be a potential desired avenue to facilitate significant breakthroughs for deep‐seated cancer PDT.^[^ ^13^ ^]^

Based on this property, we designed a nanocarrier containing substrate of gelatinases (matrix metalloproteinase 2/9, MMP2/9), which highly and extensively expressed in mostly solid tumor tissues to trigger the release of verteporfin and the generation of abundant ^1^O_2_ through CDRT. Specifically, the main designs of this study lie in the following: 1) according to the oxygen fixation hypothesis, radiation directly and indirectly induces DNA damage fixation and DNA double‐strand breaks (DSBs) due to producing DNA radicals and reactive oxygen species by ionizing radiation (IR). Should tumor‐targeted verteporfin be X‐ray‐activatable, the radiotherapy could be directly sensitized through high levels of ROS triggered by X‐rays in local tumors. 2) Meanwhile, the verteporfin‐mediated radiosensitization could reverse the immunosuppressive TME after HDRT and enhance systemic antitumor immune responses by massive ROS‐induced tumor cells death, leading to better primary tumor and metastasis suppression, and synergistically promoting cancer immunotherapy. 3) Unlike visible or near‐infrared (NIR) light, it is expected that X‐ray‐induced PDT (X‐PDT) has shown promises as a more advantageous treatment for deep‐seated tumors in reforming the traditional PDT by better tissue penetration and superior dose distribution characteristics of X‐rays with the lower dose delivered to normal tissues but highest dose deposited onto the tumor site. In this work, we treated tumor‐bearing mice with precise RT utilizing 6 MeV ionizing radiation as external excitation sources for PDT and minimized X‐ray radiation doses to quite low values (2 Gy per fraction) in order to obtain high‐dose radiotherapy effect; meanwhile, to minimize the injury risk of normal tissues surrounding the tumor as verteporfin will not be released there. 4) The design of this amphiphilic nanoemulsifying and gelatinase‐responsive drug delivery system is simple, safe, and effective, which are major concerns of cancer‐specific delivery. It can improve aqueous solubility and the in vivo half‐life of poorly water‐soluble VP by encapsulating hydrophobic VP inside the hydrophobic core, and also realize efficient targeted‐VP delivery to tumor site and controlled release, thus improving the safety profile and cancer therapeutic effect of verteporfin.

**Figure 1:**
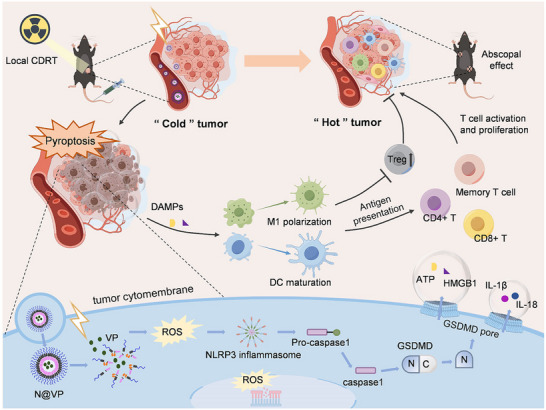
Schematic illustration. Schematic illustration of gelatinase‐responsive and N@VP‐mediated nanovaccine enhancing antitumor immune response and inducing immunological memory based on CDRT in the cancer treatment.

So far, however, there has been little detailed investigation of VP‐mediated X‐PDT and its application in cancer treatments. Our research has constructed and synthesized VP‐loaded methoxypolyethyleneglycol–PVGLIG–polycaprolactone (mPEG–PVGLIG–PCL) nanoparticles (N@VP), realizing both active‐targeting X‐PDT and cascaded ROS‐boosted sensitizing radiotherapy. The study presented in this paper is the first evaluation focused on N@VP‐mediated conventional‐dose X‐PDT to achieve parallel effects of HDRT and additional tumor vaccine effects, and also the first investigation to examine in detail the regulation of the complex tumor immune microenvironment (TIME) and the related molecular mechanism. This research has revealed that tumor‐targeted N@VP stimulated by conventional dose X‐ray irradiation induced Gasdermin‐D‐mediated pyroptosis, a potent form of immunogenic tumor cell death facilitating damage‐associated molecular pattern molecules (DAMPs) and antigens release, antigen presenting cells (APCs) recruitment, and then more effector T cells and memory T cells activation, thus inducing the generation of immunological memory which contributed to verteporfin‐mediated in situ nanovaccination (**Figure** **1**).

## 2 Results

### 2.1 Verteporfin Could Sensitize Radiotherapy by Inducing ^1^O_2_ Generation, DNA Damage, and Cell Proliferation Inhibition

^1^O_2_ generation is the crucial enabler in improving radiotherapy sensitization and detected with singlet oxygen sensor green (SOSG) fluorescent probe. The increment of SOSG intensity as a function of VP concentration and X‐ray dosage is plotted in Figure S1a (Supporting Information). It showed that VP activated by X‐rays led to radiation and drug dose‐dependent increment of ^1^O_2_ level. VP + IR demonstrated a higher ability of ^1^O_2_ generation, resulting in about 2–3‐fold increase, compared to VP or IR alone (Figure S1b, Supporting Information). The influence of ambient light during experiments on SOSG intensity was excluded (Figure S1c, Supporting Information). Accumulated intracellular ROS are well considered as mediators to induce DSBs, the most common DNA lesions, which can be detected by γ‐H2AX level.^[^ ^14^ ^]^ The mean fluorescence intensity (MFI) of γ‐H2AX measured using flow cytometry also demonstrated a positive correlation with irradiation dosage and VP concentrations (Figure S2a, Supporting Information). And the γ‐H2AX^+^ percentages of Lewis lung carcinoma (LLC) cells were greatest in the VP + IR group, which indicated that VP + IR brought about more DNA damage than VP or IR alone (Figure S2b, Supporting Information). To investigate the long‐term ability of LLC cells to form colonies from single cell after different treatments, we conducted clonogenic assay which was considered as the gold standard for assessment of radiosensitizing effects and calculated the survival rate. The results showed that there was a significant difference of survival rate between the VP + IR group and IR alone (Figure S3a, Supporting Information). Moreover, the fewer colonies counted in the VP treatment before IR group compared to VP treatment after IR group further confirmed that X‐ray‐activated VP increased LLC sensitivity to IR (Figure S3b, Supporting Information).

### 2.2 Synthesis and Characterization of N@VP

**Figure 2:**
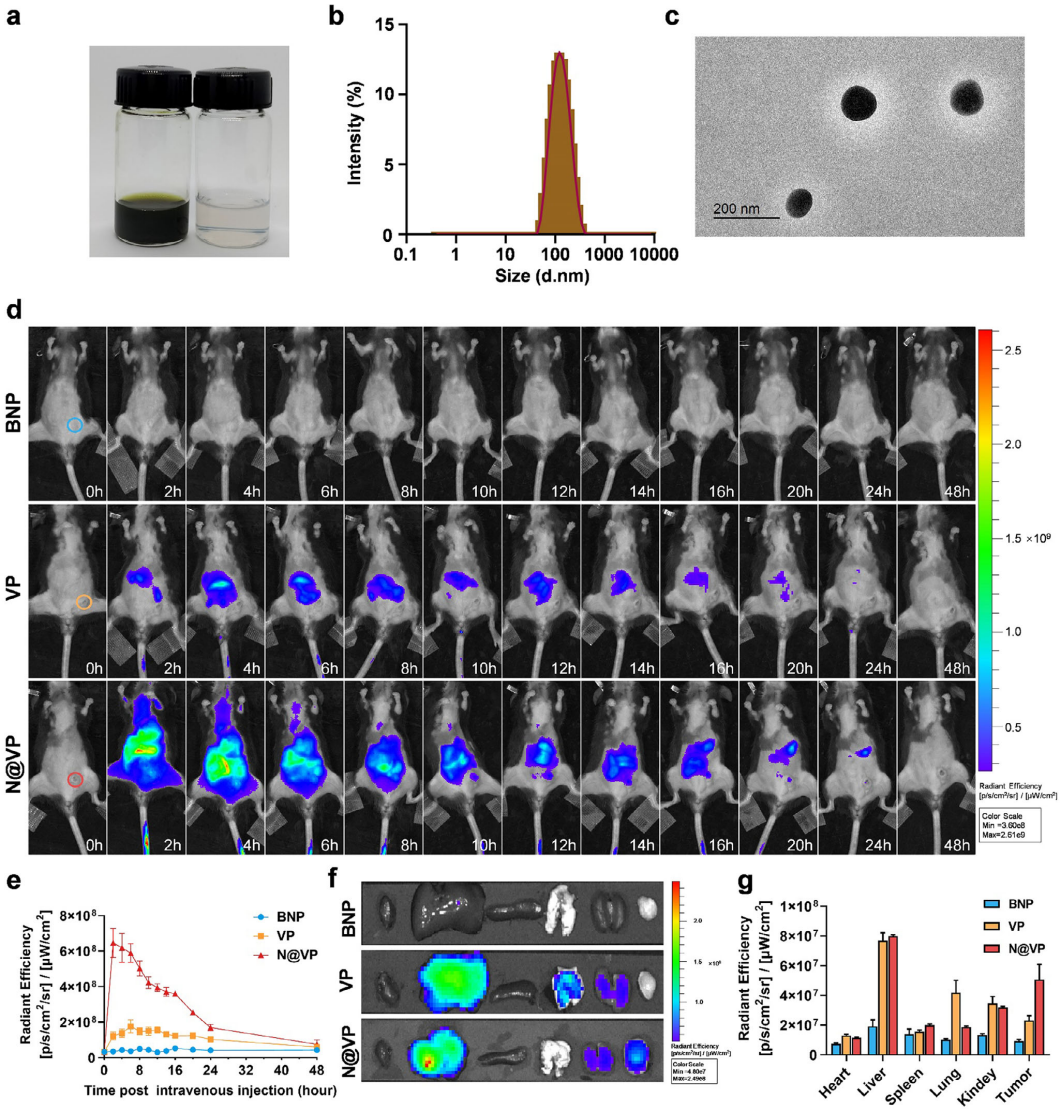
Characterization of N@VP. a) Photographs of the solution of synthesized N@VP (left) and blank nanoparticles (BNP) (right) in PBS. b) Diameter and c) TEM image of N@VP. Scale bar is 200 nm. d) In vivo NIR imaging at different times of the LLC‐bearing mice after tail intravenous injection of BNP, VP, and N@VP. Tumors are circled with blue, orange, and red circles at 0 h. e) Fluorescence signal was quantified (*n* = 3). f) Ex vivo NIR fluorescent images of the major organs (heart, liver, spleen, lung, and kidney) and tumors at 16th h after injection. g) Quantification of the mean VP fluorescence intensity of the organs and tumors (*n* = 3).

Next, a gelatinase‐responsive nanocarrier was constructed for administration of VP targeting to the tumor site (Figure S4, Supporting Information). The successful synthesis of mPEG–PVGLIG and mPEG–PVGLIG–PCL was confirmed by matrix‐assisted laser desorption/ionization‐time‐of‐flight‐mass spectra (MALDI‐TOF‐MS) (Figure S5, Supporting Information) and ^1^H NMR (Figure S6, Supporting Information), respectively. Then, VP‐loaded nanoparticles were prepared through the oil‐in‐water emulsion method (**Figure** **2**a). The hydrodynamic diameter and zeta potential of the formed N@VP were 107.55 ± 5.95 nm (PDI < 0.2) and −20.05 ± 8.35 mV, with great stability for at least 1 week in phosphate‐buffered saline (PBS) (Figure S7a,b, Supporting Information). Particle size changes of N@VP in 100% fetal bovine serum (FBS) also indicated the excellent stability of this amphiphilic nanoparticle under physiological conditions (Figure S7c, Supporting Information). Transmission electron microscope (TEM) image showed that the resultant N@VP exhibited spherical morphology with homogeneous sizes at ≈100 nm (Figure 2b,c). High‐performance liquid chromatography (HPLC) chromatograms of VP (regioisomers) are shown in Figure S7d (Supporting Information), and the encapsulation efficiency and loading efficiency of VP were determined by HPLC to be 84% and 16.8% in N@VP, respectively.

MMP2 and MMP9 are special subtypes of gelatinase family, which are highly expressed in most tumor tissues and involved in cancer progression through degrading gelatin, collagens IV and V in the extracellular matrix (ECM), while the synthesized N@VP nanoparticles could release VP after being cleaved by them (Figure S4, Supporting Information). Previous studies have shown that MMP2 and MMP9 are highly expressed in most tumor tissues, whereas normal tissues/cells and adjacent normal tissues surrounding tumors exhibited negligible expression.^[^ ^15^ ^]^ Immunohistochemical analysis revealed that the positive areas of MMP2 and MMP9 staining were relatively higher in LLC tissues, which provides a preferable environment for N@VP aggregation and VP release (Figure S8a,b, Supporting Information). After incubated with collagenase IV at 37 °C in PBS for 24 h, white precipitates dissociated from blank nanoparticles (BNP) were formed in the suspension and on the inner wall of glass bottle (Figure S7e, Supporting Information). The drug release effects of N@VP were then detected. The results showed that, under 2500 µg mL^−1^ gelatinase stimulation, drug release was rapid, achieving a maximum cumulative release of 85.92% within 8 h. Similarly, 500 and 5 µg mL^−1^ gelatinase also triggered a sustained and effective drug release, with cumulative rates of 76.39% and 53.78% over 24 h, respectively, indicating effective gelatinase responsiveness of nanoparticles in vitro (Figure S7f, Supporting Information). Figure S7g (Supporting Information) shows that the measurable VP signal was higher in VP‐treated or N@VP‐treated LLC cells after 3 h incubation, compared to the control group, suggesting good cell uptake capacity of N@VP in vitro. The in vitro cytotoxicity tests on tumor cells (LLC) and normal cells (human umbilical vein endothelial cells (HUVEC)) demonstrated that compared with free VP, N@VP exhibited pronounced antitumor activity against LLC cells (≈50% reduction cell viability) and little cytotoxicity to HUVEC cells even at the highest concentration, which underscores the application potential and safety of N@VP due to its tumor‐specific cytotoxicity (Figure S9a,b, Supporting Information).

Next, the in vivo gelatinase responsiveness, metabolism, and biodistribution of N@VP were studied via NIR imaging, which was essential for the evaluation of safety and feasibility of this drug delivery strategy and the selection of an optimal scheme. In vivo imaging demonstrated that, compared to BNP and VP, N@VP with a PEG outer shell exhibited a prolonged blood circulation time at early time points (0–8 h post injection), and subsequently, accumulated N@VP fluorescence could significantly be observed at tumor site during 10–16 h post injection (Figure 2d,e). In order to decrease the background fluorescence, 1,1'‐dioctadecyl‐3,3,3',3'‐tetramethylindotricarbocyanine iodide (DiR) was used as a more stable and brighter NIR‐fluorescent dye to study the specific tumor‐targeted and aggregation capability of mPEG–PVGLIG–PCL nanoparticles. Consistent with the in vivo dynamics of N@VP, DiR‐loaded nanoparticles (N@DiR) accumulated increasingly at tumor site and reached the peak about 10–16 h post injection, further indicating the in vivo gelatinase reactivity of nanoparticles (Figure S10a,b, Supporting Information).

Ex vivo NIR imaging at 16 h post injection confirmed that N@VP was mainly distributed in tumors and metabolic organs (liver/kidneys), with minimal retention in other organs including heart, lung, or spleen (Figure 2f,g). The majority of drugs, including nanoparticles, necessitate metabolism via the liver and/or kidneys for elimination from the body and the clinical pharmacokinetics of verteporfin also showed fast clearance of verteporfin by the liver (half‐life of elimination at 5–6 h which was not influenced by the age, gender, race, or mild hepatic or renal impairment),^[^ ^11^ ^]^ indicating the potential applications in the safe administration of radiosensitization to some extent. Besides, N@VP was completely metabolized over 48 h after administration and therefore did not accumulate in the body.

### 2.3 N@VP‐Sensitized Radiation Enhanced Antitumor Effect In Vitro

**Figure 3:**
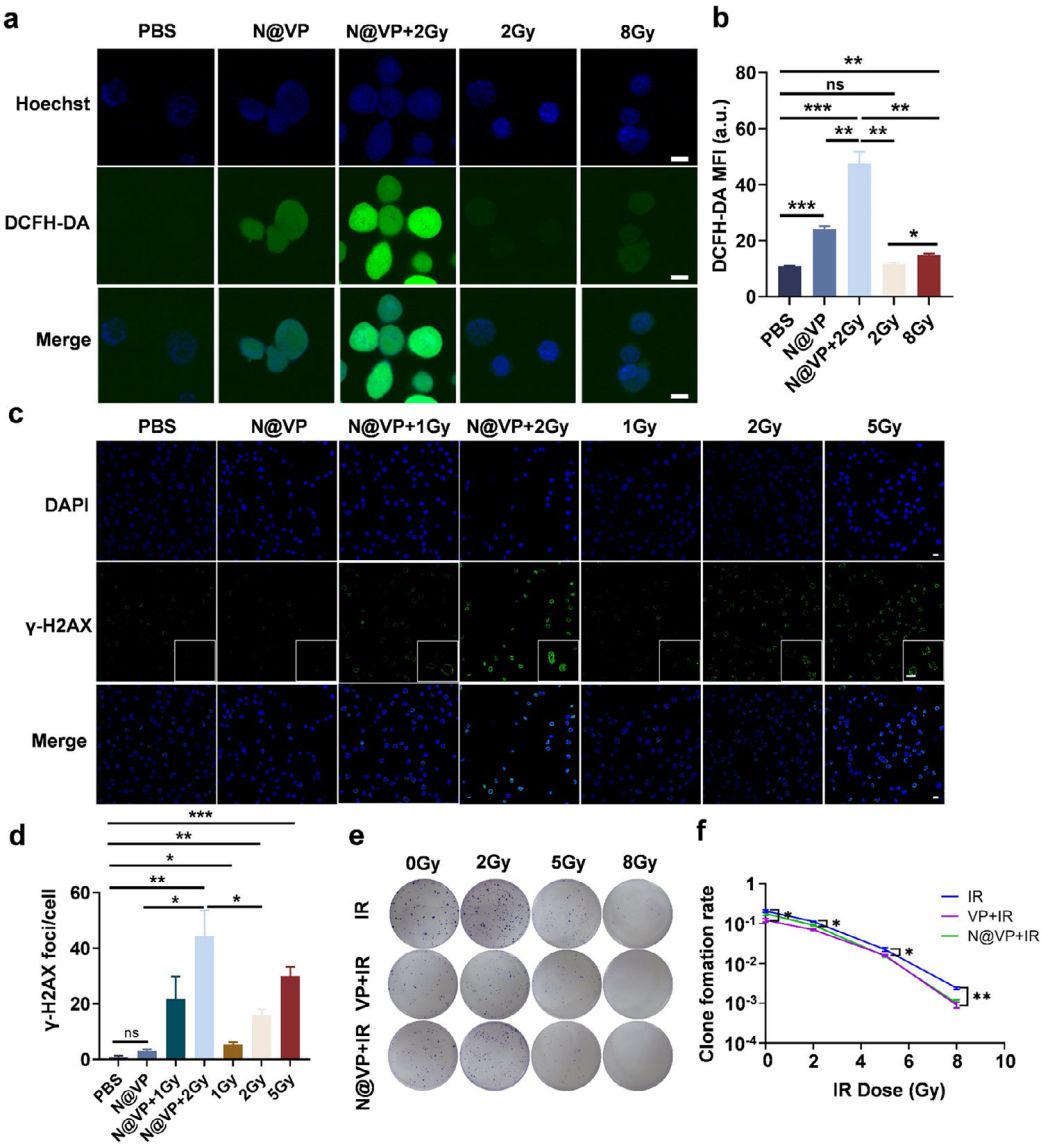
ROS generation, DNA damage, and inhibition of tumor cell proliferation induced by N@VP + 2 Gy in vitro. a) Immunofluorescence of the DCFH‐DA in LLC cells. Green: DCFH‐DA, blue: Hoechst. Scale bar: 10 µm. b) Mean fluorescence intensity of DCFH‐DA. c) Immunofluorescence of the γ‐H2AX in LLC cells. Green: γ‐H2AX, blue: DAPI. Scale bar: 10 µm. d) Counted fluorescent foci of γ‐H2AX in LLC cells. e) Clonogenic assay. The colony formation assay of LLC cells that were treated with 0/2/5/8 Gy IR alone or in combination with VP/N@VP and stained using crystal violet dye 10 days after treatment. The numbers of cells initially plated in the dish were 500 (0 Gy), 2000 (2 Gy), 5000 (5 Gy), and 10 000 (8 Gy). f) Survival rate of LLC cells after different treatments. *P*‐value was calculated by the unpaired *t‐*test, * *p* < 0.05; ** *p* < 0.01; and *** *p* < 0.001.

The synthesized N@VP was then used for in vitro radiosensitization and tumor inhibition evaluation. Under X‐ray irradiation, N@VP could exert most of its radiosensitization through intracellular ROS level increment and aggravated DNA damage. Intracellular ROS level was detected by 2′,7′‐dichlorodihydrofluorescein diacetate (DCFH‐DA) assay and the brightest DCFH‐DA intensity in N@VP + 2 Gy group was indicative of more intracellular ROS generation (**Figure** **3**a). Consistent with ^1^O_2_ detection in Figure S2a (Supporting Information), ROS level was significantly higher in 8 Gy group than in 2 Gy or PBS group (Figure 3b), while N@VP increased intracellular ROS level as well, likely owing to the cytotoxicity induced by the release of VP from nanoparticles.^[^ ^16^ ^]^

The most straightforward and efficacious approach for radiosensitization is targeting the DNA damage response directly. As shown in Figure 3c,d, more DNA‐damage‐induced γ‐H2AX foci formed with the increased irradiation dose. N@VP treatment alone inflicted little DNA damage, but when combined with 2 Gy irradiation, N@VP promoted upregulation of γ‐H2AX with a significant difference, compared to 2 Gy or N@VP alone. Subsequently, the long‐term cellular responses to DNA damage were evaluated by clonogenic survival assay. The results showed that LLC cell proliferation was suppressed by free‐VP (3.2 µm) incubation but not N@VP (3.2 µm VP). In contrast to IR group, clone formation rate of LLC cells was significantly decreased when treated with IR after N@VP incubation, further confirming that N@VP has a robust efficacy in sensitizing tumor cells to radiotherapy (Figure 3e,f).

### 2.4 N@VP‐Sensitized Radiation Enhanced Antitumor Effect In Vivo

Encouraged by in vitro studies, N@VP radiosensitizers were used for further in vivo antitumor investigation. First, we treated LLC‐bearing C57BL/6 mice randomly with PBS, N@VP alone, CDRT alone, HDRT alone, and N@VP combined with CDRT when tumor volume was around 100 mm^3^. To determine the antitumor effect of N@VP combined with CDRT, all mice of each group received respective treatment only once (Figure S11a, Supporting Information). Based on in vitro results, we chose 2 Gy as the irradiation dose for CDRT and 8 Gy for HDRT. Injection of N@VP was administered intravenous (i.v.) 16 h prior to RT on day 0, because nanoparticles mainly reached a peak in tumor tissues at 16 h according to NIR data. During 10 days of tumor volume measurement, only 8 Gy RT treatment and N@VP + 2 Gy treatment achieved effective tumor control (Figure S11b,c, Supporting Information). The photos and weight of tumors in different groups recorded at the end of experiment further demonstrated the excellent radiotherapy sensitizing effects of N@VP (Figure S11d,e, Supporting Information). There were no significant changes in both mice weight and the morphological structures of main organs among various groups (Figures S11f and S12, Supporting Information). Hematoxylin–eosin (H&E) staining of tumor tissues showed that tumor cells were sparsely packed and exhibited large areas of necrosis in N@VP + 2 Gy group, which demonstrated that N@VP combined with CDRT had obvious higher tumor‐killing efficacy with sufficient biosafety (Figure S13, Supporting Information).

**Figure 4:**
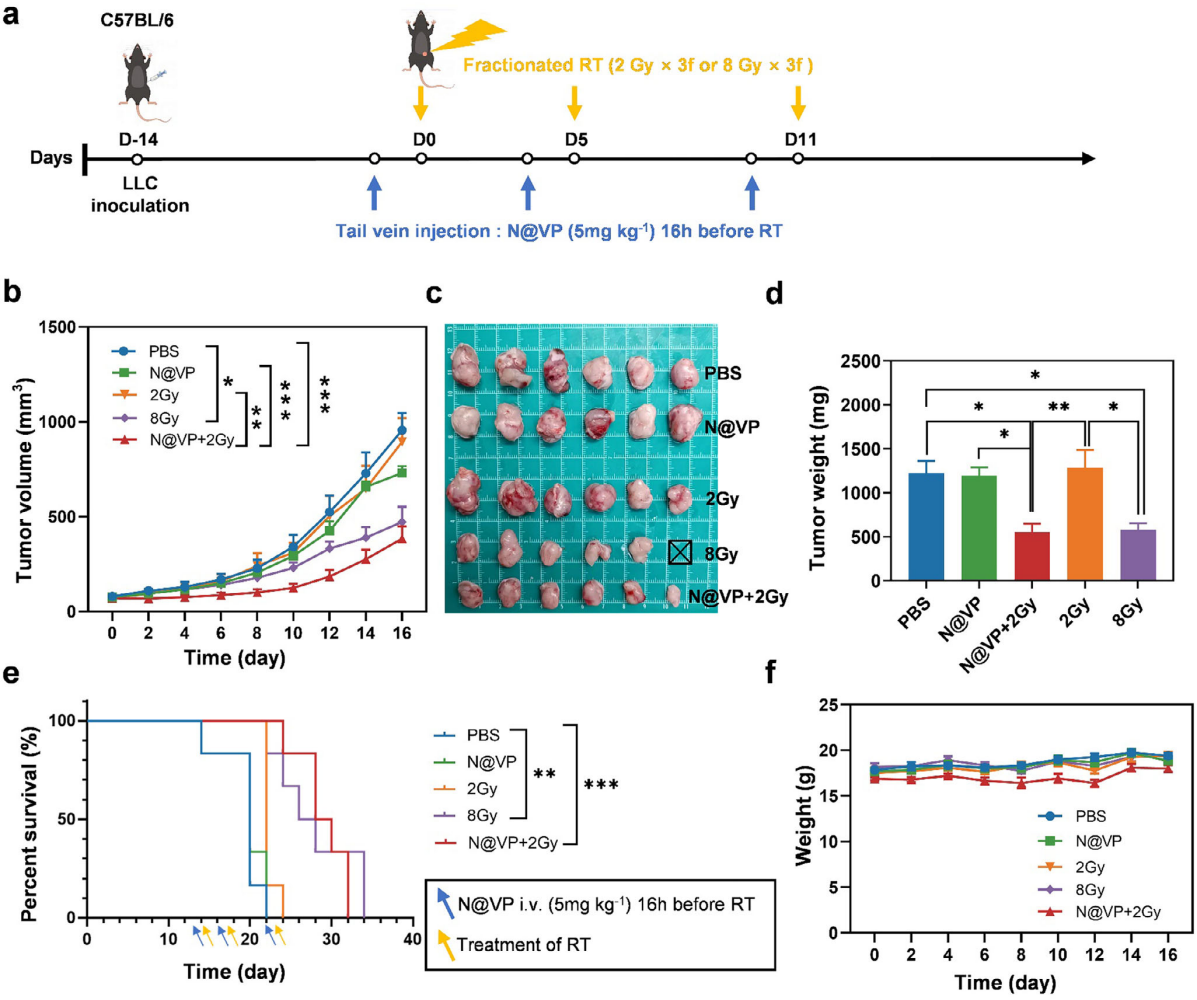
Antitumor effects of N@VP‐sensitized radiation. a) Schematic diagram of the experimental design. b) Tumor growth of mice bearing Lewis lung carcinoma in different groups (*n* = 6 in each group). *P*‐value was calculated by two‐way analysis of variance (ANOVA). *P*‐value: * *p* < 0.05; ** *p* < 0.01; and *** *p* < 0.001. c) Photographs of tumors isolated on D17. A mouse in 8 Gy group was dead on D10. d) Weight of tumors isolated on D17. One‐way ANOVA was used for comparisons. *P*‐value: * *p* < 0.05 and ** *p* < 0.01. e) The survival curve of mice in each group. Blue and yellow arrows indicate the time of tail vein administration and RT, respectively. f) Body weight curves (*n* = 6).

Next, we repeated the in vivo experiments but increased the number of treatments to three times. The LLC‐bearing mice were treated on days 0, 5, and 11 with various therapies (**Figure** **4**a). The results were congruent and further confirmed that N@VP combined with conventional‐dose hypofractionated RT (2 Gy × 3*f*) could exert more powerful tumor‐suppressive effect via radiosensitization than N@VP or 2 Gy × 3*f* alone, and this effect was comparable with that achieved by extremely high‐dose hypofractionated RT (8 Gy × 3*f*) (Figure 4b; Figure S14, Supporting Information). Tumor weights were lower in N@VP + 2 Gy and 8 Gy groups, compared to other groups (Figure 4c,d). Furthermore, survival was significantly prolonged in 8 Gy group as well as N@VP + 2 Gy group with median survival of 27 and 29 days, respectively (Figure 4e). Delightedly, in the N@VP + 2 Gy treatment group, there were no evident changes in weight measures and various serum biochemistry indexes including liver function, renal function, and cardiac function, indicating the favorable biological safety of this therapy (Figure 4f; Figure S15, Supporting Information). H&E staining of eyes from mice at the end of the fourth week following the last treatment revealed little significant chronic damage to the retina, choroid, and optic nerve, further underscoring the safety of our treatment approach in terms of ocular toxicity (Figure S16, Supporting Information).

In order to confirm that the synergistic effect of N@VP with radiotherapy is not solely dependent on VP or nanoparticles, extra control groups including VP, VP + 2 Gy, BNP, BNP + 2 Gy, and VP‐loaded non‐gelatinase‐responsive nanoparticles (mPEG‐PCL@VP) combined with 2 Gy irradiation were added. Mice were randomly assigned to different groups (*n* = 6) and received respective treatments according to the schema presented in Figure S17a (Supporting Information). The results showed significant tumor inhibition in N@VP + 2 Gy group and inconspicuous suppression of tumor growth in other groups as compared to the PBS group (Figure S17b,c, Supporting Information). Similarly, compared to N@VP + 2 Gy group, minimal inhibitory effect on tumor growth after both BNP or BNP + 2 Gy treatment excluded antitumor effect from BNP or a synergistic effect between BNP and irradiation, further reinforcing the role of VP combined with gelatinase‐responsive nanoparticles as an effective radiosensitizer for cancer therapy (Figure S18a–c, Supporting Information). No significant changes in mouse body weight were noted across different treatment conditions (Figures S17d and S18d, Supporting Information).

### 2.5 N@VP‐Enhanced Radiotherapy Augments Antitumor Immunity through Inducing Tumor Pyroptosis

**Figure 5:**
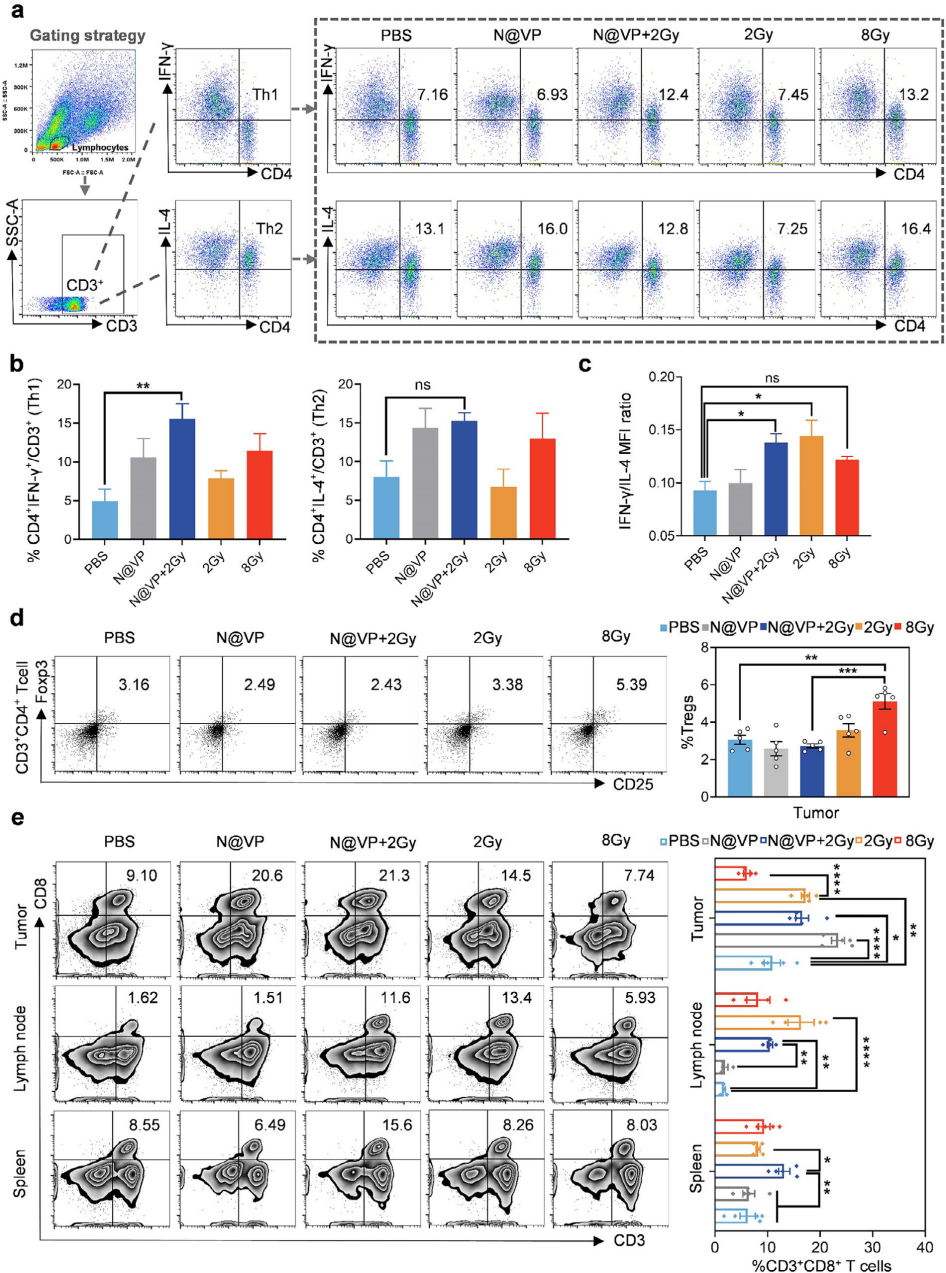
The immune microenvironment improved by N@VP + 2 Gy. a) Illustration of gating strategy and representative flow cytometry plots with corresponding quantification showing percentages of Th1 (CD4^+^ IFN‐γ^+^) and Th2 (CD4^+^IL‐4^+^) cells (gated on CD3^+^ T cells) in tumor tissues after various treatments. b) Th1 and Th2 cells percentages (*n* = 5) on CD3^+^ gate. c) Quantification of IFN‐γ/IL‐4 MFI ratio (*n* = 5). d) Representative flow cytometry dot plots of CD25^+^Foxp3^+^ cells gating on CD3^+^CD4^+^ T cells in tumors and the percentages of Treg cells’ (CD3^+^CD4^+^CD25^+^Foxp3^+^) populations in each group presented as histograms. e) Representative flow cytometry study of CD3^+^CD8^+^ T cells with corresponding quantification in tumors, lymph nodes, and spleens after various treatment stated above. *P*‐value of the data above was calculated by one‐way ANOVA (ns represents *p* > 0.05; *p < 0.05; **p < 0.01; ***p < 0.001; and ****p < 0.0001).

Based on the remarkable tumor suppressive ability and prolonged survival, we further investigated the immune status changes by flow cytometric analysis of the immune cell populations in tumors and lymphoid organs after various treatments that are illustrated in Figure 4a, in order to provide a mechanistic understanding of immune response and antitumor effect induced by N@VP‐enhanced radiotherapy. First, we studied the infiltration of T cells including T help (Th) cells, regulatory T cells (Treg cells), and CD8^+^ T cells in TME, which play crucial roles in antitumor immune process. Th1 cells, primarily responsible for cell‐mediated immune responses, are characterized by the secretion of Th1 cytokines such as tumor necrosis factor alpha (TNF‐α), interleukin (IL)‐2, and interferon‐gamma (IFN‐γ), which could directly induce tumor cellular apoptosis and stimulate cytotoxic T lymphocytes’ (CTLs) proliferation and differentiation,^[^ ^17^ ^]^ whereas Th2 cells predominantly secret IL‐4, which promotes the activity of immunosuppressive cells and suppresses Th1‐cell differentiation, leading to tumor immune evasion.^[^ ^18^ ^]^ Our results demonstrated that the percentages of Th1 cells in the tumor were apparently increased in N@VP + 2 Gy group (**Figure** **5**a,b). There were no notable differences of Th2 cell percentages among the five groups. Both 2 Gy and N@VP + 2 Gy treatment displayed an evident trend toward upregulating the ratio of IFN‐γ to IL‐4 expressed by CD3^+^CD4^+^ T cells, indicating a significant Th1‐mediated antitumor immune responses activation (Figure 5c). Another key subset of CD3^+^CD4^+^ T was FOXP3‐expressing Treg cells, functioning as negative modulators in antitumor immune response. Several studies have signified that increased infiltration of Treg cells into tumor tissues was often associated with tumor progression and poor prognosis.^[^ ^19^ ^]^ It was found that 8 Gy radiotherapy evoked a significant augment in the proportion of FOXP3^+^CD25^+^ Treg cells in tumor tissues. By contrast, Treg cell percentage was relatively lower in the N@VP + 2 Gy group (Figure 5d).

Effective antitumor immunity and immunological memory require T‐cell‐mediated in situ tumor immune response cooperating with peripheral immunity. To provide a clear elucidation of systemic antitumor immune reaction, we further quantitate the recruitment of CD8^+^ T cells into tumor tissues and secondary lymphoid organs (spleen and lymph nodes) using flow cytometry (Figure 5e). The results showed that N@VP, 2 Gy, and N@VP + 2 Gy treatment all had significantly promoted CD3^+^CD8^+^ T cell infiltration into tumor tissues, while the amount of CD3^+^CD8^+^ T cells in both spleen and lymph nodes was only significantly increased in the N@VP + 2 Gy group, indicating a more potent immune response triggered by N@VP‐enhanced radiotherapy. Furthermore, compared with PBS group, both CDRT (2 Gy) and HDRT (8 Gy) tended to promote CD3^+^CD8^+^ T cell infiltration into the draining lymph nodes, the main sites of antigen presentation and naïve T‐cell activation, while CD3^+^CD8^+^ T cell infiltration was more prominent in the CDRT group than that in the HDRT group (*p* < 0.0001 vs *p* = 0.05).

**Figure 6:**
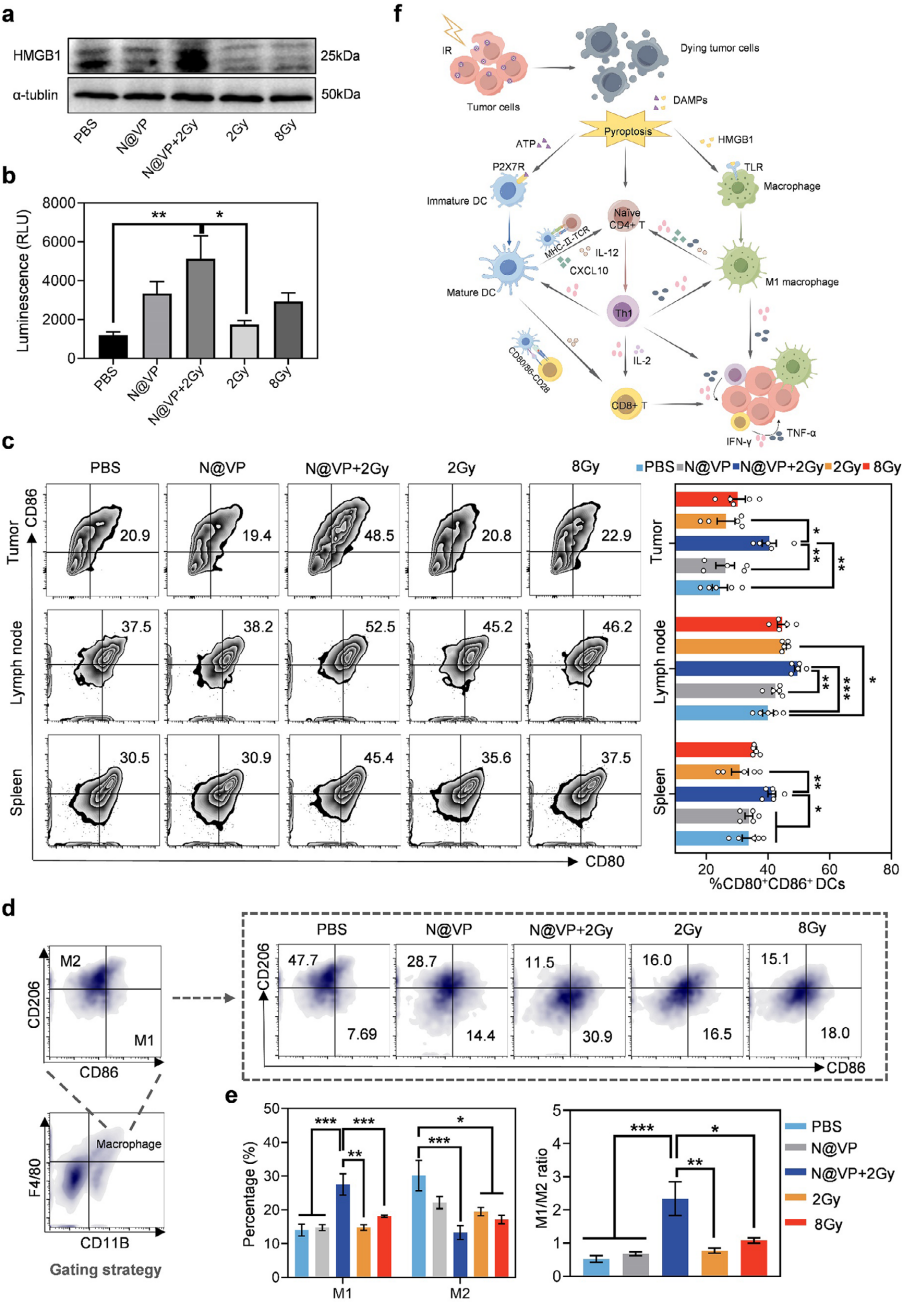
N@VP‐mediated X‐PDT induced immunogenic tumor cell death and APCs’ activation. a) Western blot bands of HMGB1 collected from tumor tissues at the sixth hours after different in vivo treatments. b) Release of ATP into the cell culture supernatants was measured luminometrically using an ATP assay kit. The supernatants were collected 12 h after treatments of PBS/N@VP in combination with 0/2/8 Gy. c) DC maturation in tumors, lymph nodes, and spleen tissues after various treatment in vivo was evaluated by surface co‐expression of CD80 and CD86 on CD11C^+^ DCs using flow cytometry. The percentages of CD80^+^CD86^+^ on CD11C^+^ gate was shown as histograms. d) Gating strategy of macrophages (CD11B^+^F4/80^+^), which were subdivided into M1 (CD86^+^CD206^‐^) and M2 (CD86^‐^CD206^+^) macrophages. Representative flow cytometry plots with e) corresponding quantification of M1 and M2 macrophage percentages, and calculated M1/M2 ratio was shown as well (*n* = 5 in each group). f) The schematic illustration of various immune responses induced by N@VP‐mediated X‐PDT. Data above were evaluated by one‐way ANOVA (ns represents *p* > 0.05; *p < 0.05; **p < 0.01; ***p < 0.001; and ****p < 0.0001).

Immunosurveillance is generally mediated by Th1 cells and CD8^+^ T cells, which take effect through recognizing specific antigenic epitopes emerging during malignant tumor progression.^[^ ^20^ ^]^ However, antigenicity of malignant cells is not sufficient enough to initiate T‐cell‐mediated anticancer immunity directly, and the activation of both naïve CD4^+^ and CD8^+^ T cells is dominantly mediated by mature APCs presenting tumor antigens in secondary lymphoid organs. As shown in **Figure** **6**a, compared to other groups, N@VP under X‐ray irradiation treatment caused salient overexpression of the high mobility group box 1 (HMGB1) protein in tumor tissues. In addition, we detected adenosine triphosphate (ATP) concentrations in LLC cell culture supernatant of all the treatment groups and found that more ATP was released from LLC cells that were treated with N@VP + 2 Gy (Figure 6b). HMGB1 and ATP are unique molecule types of DAMPs that are actively secreted, or passively released by stressed, injured or dying cells. Releasing DAMPs is an essential hallmark of treatment‐driven immunogenic cell death (ICD), for contributing to modulating the immune environment, most importantly APCs’ activation. Therefore, we assessed dendritic cells’ (DC) maturation and M1/M2 macrophages polarization by analyzing CD80^+^CD86^+^ DCs and CD86^+^M1/CD206^+^M2 macrophages using flow cytometry. As expected, N@VP + 2 Gy was able to prominently trigger more mature DC recruitment to tumor tissues and secondary lymphoid organs (Figure 6c). Furthermore, compared to PBS group, CDRT can significantly promote more mature DCs’ infiltration in the draining lymph nodes than HDRT (*p* = 0.04 and *p* = 0.11, respectively). Apart from CD80^+^CD86^+^ upregulation, N@VP + 2 Gy can also significantly promote the expression of major histocompatibility complex (MHC) class II, commonly expressed on APCs’ surface and considered as the most important complexes involved in antigen presentation by APCs (Figure S19a,b, Supporting Information). However, higher expression of MHC‐I was only observed in the 8 Gy group (Figure S19c,d, Supporting Information).

M1 macrophages percentages in TME as well as M1/M2 ratio were evidently increased in the N@VP + 2 Gy group while the proportion of M2 macrophages was considerably decreased in the N@VP + 2 Gy group compared to PBS group (Figure 6d,e). This was an exciting result signifying that N@VP‐enhanced radiotherapy greatly induced the macrophages polarizing from an anti‐inflammatory M2 phenotype to a proinflammatory M1 phenotype, which plays a vital role in antitumor response.

Collectively, the above results demonstrated that N@VP‐mediated X‐PDT was capable of relieving the immunosuppressive state and achieving antitumor effects by remodeling TIME (Figure 6f). It is reported that tumor immunogenicity induced by ICD would be more effective if it was fostered by focused ROS‐based stress such as PDT rather than by secondary or collateral stress.^[^ ^21^ ^]^ Thus, when tumor‐targeted N@VP cooperated with highly penetrated X‐rays, it tended to make more strongly immunogenic tumor by spatiotemporally stimulated emission of DAMPs and exposure of specific antigenic epitopes in the course of ICD. DAMPs binding to specific pattern recognition receptors (PRRs) expressed by APCs can initiate a cellular cascade of adaptive immune responses via distinct PRRs driving chemotactic movement, homing, activation, and maturation, finally leading to the cross presentation of tumor antigens to T cells.^[^ ^20^ ^]^

**Figure 7:**
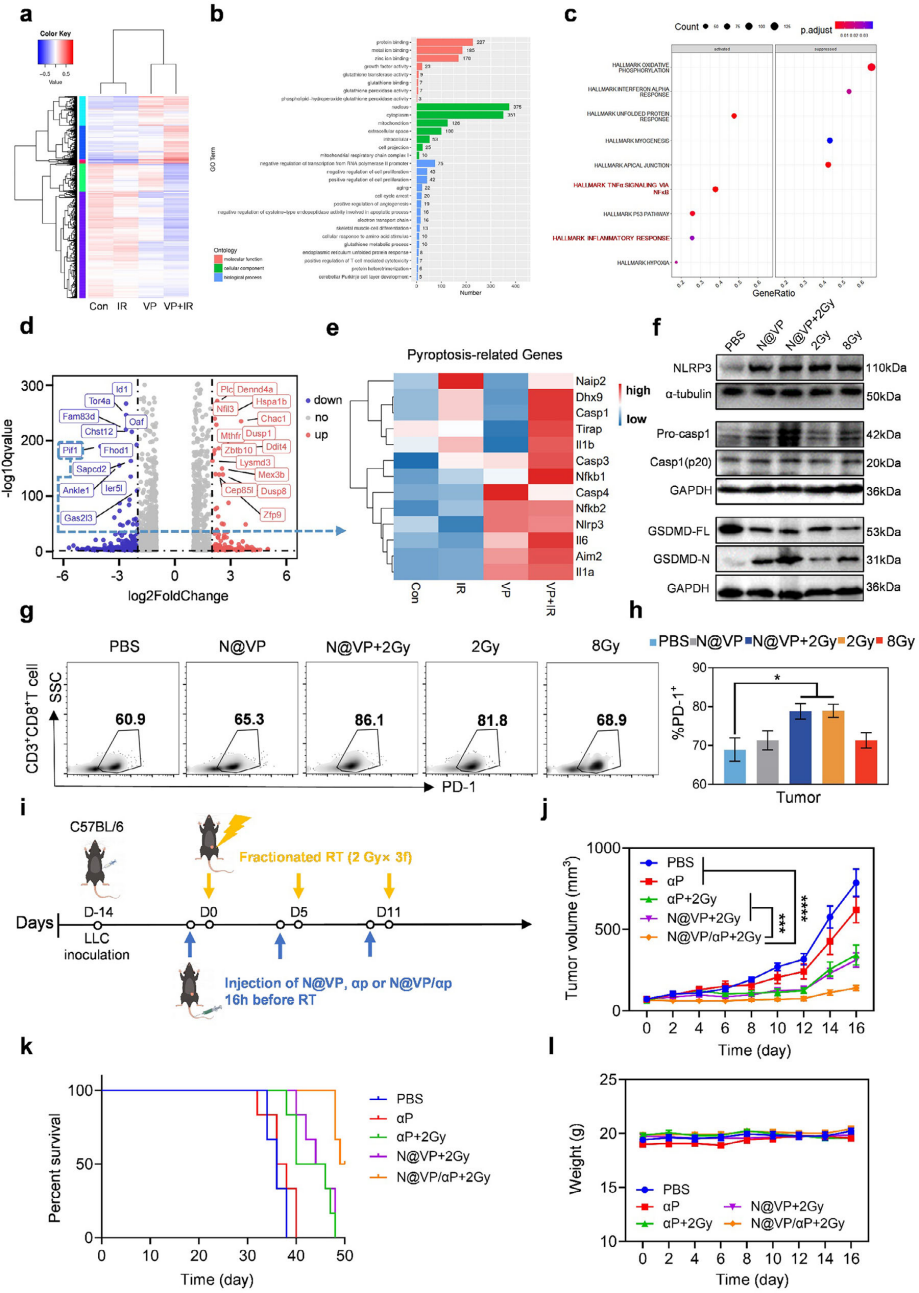
N@VP‐mediated radiosensitization promoted activation of pyroptosis in tumor cells to amplify immunogenic cell death and enhanced the efficacy of immunotherapy by upregulating PD‐1 expression on CD8^+^ T cells. a) Heatmap of DEGs of LLC cells in Con/IR/VP/VP+IR group. b) Gene Ontology (GO) enrichment analysis focused on comparing the gene expression differences between the Con group and the VP + IR group. c) GSEA identified the gene sets significantly enriched in Hallmark by DEGs in the two groups’ RNA sequencing data (VP + IR vs Con), *p*.adjust < 0.05. d) Volcanic maps of DEGs between two groups (VP + IR vs Con) were shown. Red points represent upregulated genes in VP + IR‐treated group while blue points represent downregulated genes. e) Heatmap of DEGs of pyroptosis pathway in each group. f) Western blot protein expression bands of tumor tissues isolated from C57BL/6 mice at 6^th^ hour after various treatments. g) Representative flow cytometry profiles and h) corresponding quantification of PD‐1^+^ expression on CD3^+^CD8^+^ T cells in tumor tissues from each group. *P*‐value was evaluated by one‐way ANOVA (*p < 0.05; **p < 0.01; ***p < 0.001; and ****p < 0.0001). i) Schematical illustration of the experimental procedure and the graphs of j) tumor growth, k) mice survival, and l) body weight in each group (*n* = 6).

After studying the altered immune effector cells and immune‐stimulating components in the TIME and lymphoid tissues, we then examined the differentially expressed genes (DEGs) and specific signaling pathways that may contribute to tumor cell immunological death and antitumor immunity caused by this X‐PDT treatment through high‐throughput RNA sequencing (RNA‐seq). Heatmap of DEGs showed that more genes were upregulated and downregulated in the VP + IR group (**Figure** **7**a). Compared to control group (Con), the enriched biological process in VP + IR group was mostly related to negative regulation of transcription from RNA polymerase II promoter, regulation of cell proliferation, aging, and cell cycle arrest (Figure 7b). Gene set enrichment analysis (GSEA) indicated that VP + IR treatment significantly activated inflammatory response and TNFα signaling via nuclear factor kappaB (NFκB) (Figure 7c; Figure S20, Supporting Information), whereas Kyoto Encyclopedia of Genes and Genomes (KEGG) pathway analysis further confirmed that DEGs were mainly involved in TNF signaling pathway and other inflammatory response‐related signaling pathways, such as phosphatidylinositol 3‐kinase (PI3K)–protein kinase B (Akt), mitogen‐activated protein kinases (MAPK), janus kinase (Jak)‐signal transducer and activator of transcription (STAT), forkhead box O (FoxO), and erythroblastic leukemia viral oncogene homologue (ErbB) signaling pathways (Figure S21, Supporting Information). These enriched pathways may be involved in the responses of tumor cell ICD.

Then, we identified and labeled the 25 genes most significantly up/downregulated in VP + IR group (Figure 7d) to find the key genes and activated pathways that induced the radiosensitization through enhanced DNA damage. Among these upregulated genes, *Dennd4a*, *Nfil3*, *Hspa1b*, *Chac1*, *Ddit4*, *Mex3b*, *Lysmd3*, *and Zbtb10* are mainly involved in oxidative stress and inflammation response, whereas *Plcxd2*, *Dusp1*, *Cep85l*, and *Dusp8* are generally considered as tumor‐suppressor genes by inhibiting proliferation of cancer cells. For the downregulated genes, *Id1*, *Tor4a*, *Fam83d*, *Chst12*, *Fhod1*, *Sapcd2*, and *Gas2l3* are known as proliferative and metastatic oncogenes contributing to tumor progression. Noteworthy, *Ankle1* downregulation has been found to evoke prolonged activation of the DNA damage response.^[^ ^22^ ^]^ And *PIF1* knockdown is also reported to elevate radiation efficiency by mediating cell pyroptosis through the DNA damage pathway.^[^ ^23^ ^]^ Heatmap manifested that VP + IR increased the level of DEGs related to pyroptosis (Figure 7e). In vitro results further demonstrated that N@VP combined with 2 Gy IR treatment significantly induces LLC cells to secret higher levels of IL‐1β and IL‐18 (Figure S22a,b, Supporting Information) and the cells exhibited typical pyroptosis morphology with cells swelling and membrane blebbing (Figure S22c, Supporting Information). To verify the occurrence of pyroptosis after N@VP‐mediated X‐PDT in vivo, we examined the expression of critical pyroptosis‐related proteins in tumor tissues via western blot assay, including domains‐containing protein 3 (NLRP3), cysteine aspartic acid‐specific protease (Caspase1, Casp1), Pro‐casp1, full length gasdermin D (GSDMD‐FL), and cleaved GSDMD (GSDMD‐N) (Figure 7f). The results confirmed the previous findings that obtained by RNA‐seq analysis, potently substantiating that N@VP‐mediated X‐PDT could activate the GSDMD‐dependent pyroptosis pathway, which was a newly uncovered manner of immunogenic cell death and characterized by DNA breakage, chromatin condensation, cell enlargement, membrane pores forming, membrane blebbing, bursting, and cytokine release.^[^ ^24^ ^]^

### 2.6 N@VP‐Enhanced Radiotherapy Induces Systemic Antitumor Immune Effect and Immune Synergism

Except for improved tumor immunogenicity, blocking immune checkpoints is another effective approach to obstructing tumor immune escape. Immunotherapy dominated by programmed death‐1/programmed death‐ligand 1 (PD‐1/PD‐L1) checkpoint inhibitors has recently emerged as a standard of treatment for advanced‐stage NSCLC.^[^ ^25^ ^]^ These ICI therapies aim to enhance local immune response against tumor by means of disrupting the binding of PD‐1 on the T‐cell surface and PD‐L1 on the tumor cell surface and restricting PD‐1/PD‐L1‐mediated T‐cell suppression. However, clinical trials have revealed that immunotherapy alone may not achieve a considerable and persistent therapeutic response.^[^ ^26^ ^]^ In our study, we found that the infiltration of CD8^+^ T cells significantly increased in the tumor, lymph node, and spleen when treated with N@VP in combination with CDRT (Figure 5e). Additionally, the expression of PD‐1 on CD8^+^ T cells was higher (Figure 7g,h). This suggests a more energetic response and promising synergistic effect when combined with anti‐PD‐1 antibody (αP) in cancer therapy. Therefore, to further strengthen our hypothesis, we then investigated the in vivo combination of CDRT enhanced by N@VP together with PD‐1 blockade therapy. In this experiment, mice were randomly divided into five groups (PBS, αP, αP + 2 Gy, N@VP + 2 Gy, and N@VP/αP + 2 Gy) and received different treatments once a week for a duration of 3 weeks, when the tumor volume reaches 50–100 mm^3^ (Figure 7i). The administered doses of αP and VP were 10 and 5 mg kg^−1^, respectively, in the N@VP, N@VP/αP and the free αP injected into each mouse. As previously forecasted, anti‐PD‐1 monotherapy contributed to almost little efficacy in tumor growth suppression, but such immunotherapy insensitive or tolerated status was successfully reversed when combined with 2 Gy or N@VP + 2 Gy treatment (Figure 7j; Figure S23a,b, Supporting Information). Besides, compared to αP + 2 Gy and N@VP + 2 Gy groups, N@VP/αP + 2 Gy treatment resulted in a significant extension in median survival of mice bearing Lewis lung carcinoma (Figure 7k). There were no obvious weight losses in each group (Figure 7l).

**Figure 8:**
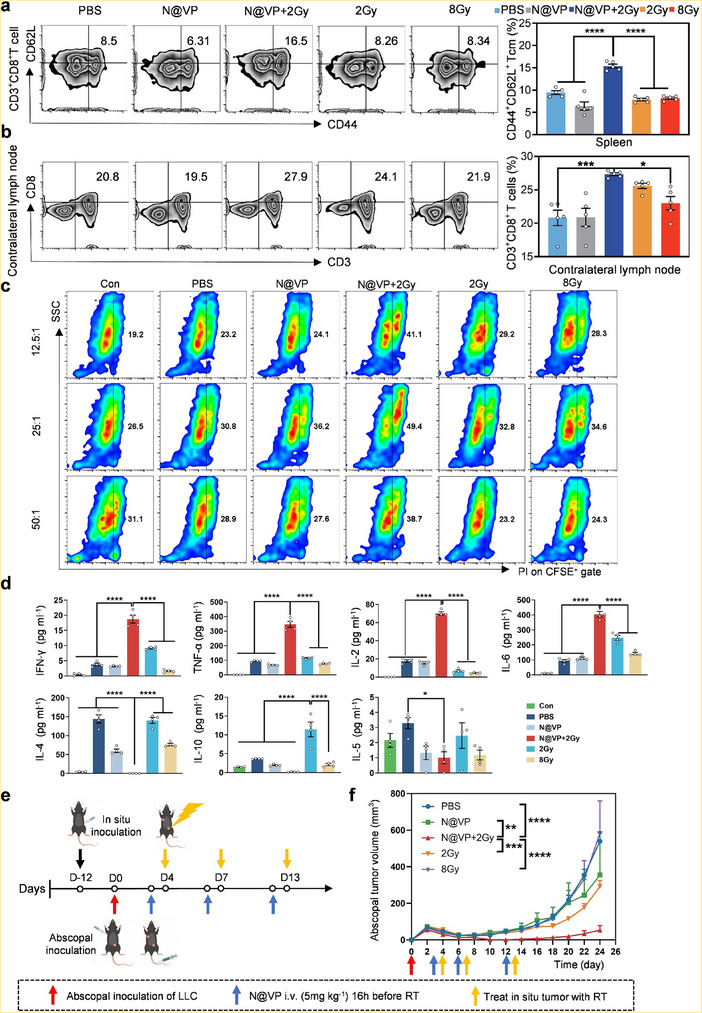
Abscopal‐antitumor effect of N@VP‐enhanced radiotherapy. a) Representative flow cytometry profiles with corresponding quantification of CD44^+^CD62L^+^ cells percentages (gated on CD3^+^CD8^+^ T cells) in spleens of C57BL/6 mice undergoing various treatments. b) Representative flow cytometry profiles with corresponding quantification of CD3^+^CD8^+^ T cells in contralateral lymph nodes of C57BL/6 mice undergoing various treatments. Data were evaluated by one‐way ANOVA (*p < 0.05; **p < 0.01; ***p < 0.001; and ****p < 0.0001). c) Killing effects of T cells against carboxyfluorescein diacetate succinimidyl ester (CFSE)‐labeled LLC cells. T cells were collected from spleens of mice in each group and then co‐incubated with CFSE‐labeled LLC cells for 6 h. Con group means normal mice without tumor or any other treatments. The figure exhibited representative flow cytometry profiles showing percentages of dead target cells (CFSE^+^PI^+^) when effective T cells/tumor cells ratios (E/T ratio) were 12.5:1, 25:1, and 50:1, respectively. d) The cytokine concentration (pg mL^−1^) of supernatants in the killing activity experiment was evaluated using the flow cytometry. *P*‐value was calculated by one‐way ANOVA (*p < 0.05; **p < 0.01; ***p < 0.001; and ****p < 0.0001). e) Schematic of the experimental procedure. The bilateral tumor model was used to assess abscopal effect produced by N@VP combined CDRT. f) Abscopal tumor growth curves (*n* = 6). *P*‐value was evaluated by two‐way ANOVA (*p < 0.05; **p < 0.01; ***p < 0.001; and ****p < 0.0001).

Based on the extraordinary antitumor immune response achieved on in situ tumor models, we wondered whether this combination therapy could also produce considerable abscopal effect on untreated distant metastases, which could promisingly be an outstanding breakthrough in cancer radiotherapy for metastatic tumors. Many studies have established that systemic and long‐term antitumor immunity depends mainly on the establishment of memory CD8^+^ T cells.^[^ ^27^ ^]^ As shown in Figure S24a,b (Supporting Information), the proportions of both CD3^+^CD8^+^ T cells and CD44^+^CD62L^‐ ^effector memory CD8^+^ T cells (Tem) were significantly elevated after N@VP + 2 Gy treatment in tumor microenvironment. However, CD44^+^CD62L^+^ central memory CD8^+^ T cells (Tcm), which are more enriched in secondary lymphoid organs (spleen and lymph nodes), have a longer lifespan than Tem and can respond more robustly to rechallenge by the same antigen, thus forming the basis of vaccines,^[^ ^28^ ^]^ were observed more infiltration in N@VP + 2 Gy group (**Figure** **8**a; Figure S25a,b, Supporting Information). N@VP‐mediated radiosensitization also increased CD3^+^CD8^+^ T cells’ infiltration in contralateral lymph nodes, indicating an effective systemic immune response (Figure 8b). Moreover, results of target‐specific killing assay in vitro demonstrated that compared with other groups, splenic T cells from N@VP + 2 Gy treatment group had more notable tumor‐killing effect when co‐incubated with tumor cells for 6 h (Figure 8c; Figure S26a,b, Supporting Information). The concentrations of immune‐promoting Th1 cytokines (IFN‐γ, TNF‐α, and IL‐2) as well as proinflammatory signal (IL‐6) determined in culture supernatants and tumor tissues were evidently higher in N@VP + 2 Gy group (Figure 8d; Figure S27, Supporting Information). The above data mutually indicated that N@VP + 2 Gy could efficiently elicit an effective robust antitumor memory immune response systemically. Such memorably tumor‐specific T‐cell immunoreaction could in theory also restrain the growth of nontreated abscopal tumors and metastatic lesions. Hereafter, to further strengthen our conclusions, 1 × 10^6^ LLC cells were inoculated in the contralateral axilla of subcutaneous LLC‐bearing C57BL/6 mice to simulate distant metastasis models before various treatments on in situ tumors (Figure 8e). For mice with primary tumors treated by PBS, N@VP, CDRT (2 Gy), or HDRT (8 Gy), obvious growth of secondary tumor that was considered as indication of steady tumor metastases showed up on day 6 after tumor regression (Figure S28, Supporting Information). Although there was a tendency of secondary tumor inhibition in 2 Gy group, the difference (*p* = 0.1797) was not conspicuous. By contrast, N@VP + 2 Gy treatment exhibited significantly delayed metastases with a distant metastasis rate of 50% and no abscopal tumor growth during the therapy, indicating strong capacity of inducing a resultful in situ nanovaccine based on local conventional‐dose hypofractionated radiotherapy (Figure 8f).

## 3 Discussion

Currently, radiation therapy is still the most commonly used and standard therapy for localized cancer, and its devices have been applicable in most cancer hospitals/departments around the world. However, the risk of irradiation‐induced complications rises with increasing dose.^[^ ^29^ ^]^ Many types of radiosensitizer have reported to accentuate the effects of radiotherapy mostly in indirect mechanisms including improving the oxygen level in tumor tissues, targeting DNA synthesis/damage/repair proteins and inducing tumor cell apoptosis.^[^ ^30^ ^]^ Although chemotherapeutic agents have been commonly tried to enhance radiotherapy efficacy clinically, it is needed for the development of a sensitizer that can advance the radiotherapy efficacy at a lower effective dose and also functions as an ideal drug to induce tumor cell death, because the superimposed systemic toxicity of combined chemo‐radiotherapy may interfere further intensification of both radiotherapy and combined therapy.^[^ ^30^, ^31^ ^]^ Different from other radiosensitizers, this study has presented a newly emerging photodynamic therapy based on X‐ray irradiation, which could directly enhance radiotherapy efficacy through increasing the generation of cytotoxic ROS and DNA breakages triggered by irradiation. Besides, compared with conventional HDRT and CDRT, this therapy has provided a relatively safe irradiation dose with decreased treatment frequency for patients requiring long‐term radiotherapy, leading to fewer complications but better antitumor efficacy. It means that such X‐PDT‐induced SBRT can achieve universal application in almost all parts of the body, resolving the main limitation of applying HDRT to the radiation‐intolerant organs and tissues. More importantly, for highly radio‐insensitive tumors, regulating DC activation status and Treg function is predominant to successfully generate effector T‐cell‐mediated antitumor immune response,^[^ ^32^ ^]^ while this therapy is also able to stimulate superior antitumor immune response than conventional SBRT, including M1 macrophage polarization, DCs’ maturation, Treg infiltration inhibition, and T‐cell activation, through inducing Caspase‐1‐GSDMD‐pyroptosis pathway in tumor cells. The gasdermin family protein, particularly GSDMD, serves a critical role in executing typical pyroptosis pathway. Upon cleavage by activated caspase1, GSDMD forms its N‐terminal domain (GSDMD‐N), which binds to cellular membrane phospholipids and creates pores on the membrane, followed by rupture of tumor cell membrane and then fast release of multiple pro‐inflammatory signals (IL‐1β and IL‐18) and cellular alarmins (ATP, HMGB1, chemokines, and other cytokines).^[^ ^33^ ^]^ These inflammatory signals amplify inflammation in tumor tissues and mobilize immune cells in response to threats. It is reported that ATP could promote DC maturation and pro‐inflammatory cytokines’ secretion,^[^ ^5c^ ^]^ leading to initiation of robust in vivo immunologic cascade, including IFN‐γ production, T‐cell amplification, plenitude polarization of CD8^+^ T cells, and decrease of Tregs.^[^ ^4^, ^34^ ^]^ HMGB1, another main component of DAMPs, acts as a “natural adjuvant” that stimulates DC maturation and upregulated IFN‐γ expression in CD8^+^ T cells.^[^ ^35^ ^]^ Besides, it is considered that HMGB1 is also one of the essential M1 polarization inducers through the NFκB–NLRP3 inflammasome pathway.^[^ ^36^ ^]^ Recent studies have shown that irradiation could also induce nontypical pyroptosis through caspase3/GSDME pathway, which is a critical determinant of radiosensitivity of nasopharyngeal carcinoma.^[^ ^37^ ^]^ However, up to date, there have been relatively a few studies reported in the area of pyroptosis‐associated mechanism by irradiation in NSCLC.

The study revealed that N@VP‐mediated X‐PDT effectively elicited tumor cell pyroptosis and potentiated tumor immunogenicity, thus facilitating APC maturation and activating immune infiltration in the tumor microenvironment (Figure 6f). Mature DC upregulated expression of MHC‐II and co‐stimulatory molecules (CD80/CD86), promoting naïve CD4^+^ T cell differentiation to Th1 CD4^+^ cells, meanwhile, more effectively activating and presenting tumor antigens to T cells.^[^ ^38^ ^]^ A polarization of M2 macrophages into M1 macrophages after N@VP‐enhanced radiotherapy is considered to be an effective antitumor immunological response inducer because M1 macrophage is a favorable macrophage subtype associated with remarkable tumor‐killing capacity via engulfing tumor cells and secreting tumor cell toxic cytokines directly. In addition, mature DC and M1 macrophages are capable of secreting IL‐12 and CXCL10 to induce Th1 differentiation and chemotactic movement, respectively.^[^ ^39^ ^]^ In return, activated Th1 cells produce IFN‐γ, TNF, and IL‐2 to exert powerful antitumor immune effect by facilitating antigen processing and presentation of DCs, promoting M1 polarization and enhancing T differentiation, survival, and activity, ultimately forming a context of robust immunostimulation with positive feedback.^[^ ^38^, ^40^ ^]^

The research also found that CDRT (2 Gy per fraction) appeared to show a significant advantage in playing immunoregulatory effect than HDRT (8 Gy per fraction). Although 8 Gy treatment prominently upregulated MHC‐I expression on DCs, the proportion of Tregs were also increased compared with other groups. The tumor‐specific immune responses seemed to be hindered by high‐dose hypofractionated radiotherapy (8 Gy × 3*f*) through the compensatory Treg‐response‐driven immunosuppressive state, which may result in increased risk of grievous treatment‐related complications post HDRT for long‐term survivors of NSCLC.^[^ ^41^ ^]^

In addition, triggering the antitumor response of the whole body is the key therapeutic strategy for malignant tumors. Under limited circumstances, radiotherapy alone can induce such an abscopal effect, but this phenomenon was relatively rare. According to a retrospective review, there were only a total of 41 reported cases of successful abscopal effect from 1969 to 2014.^[^ ^42^ ^]^ Currently, combining radiotherapy with immunologic adjuvants or immunotherapies, such as toll‐like receptors agonists, granulocyte‐macrophage colony‐stimulating factors, and especially ICIs, has been shown to improve abscopal response rates in metastatic tumor diseases.^[^ ^43^ ^]^ However, the rates were still very low. A phase I/II trial reported that no out‐field objective response and no abscopal effect were found when receiving radiotherapy in combination with anti‐PD‐L1 treatment,^[^ ^44^ ^]^ which means the strategies that could realize more resultful and stable abscopal effect on progressed tumors are still needed to be explored. Significantly, our study revealed a novel strategy based on CDRT, whereby persistent and obvious growth delays of nonirradiated secondary tumors during the treatment were observed in LLC‐bearing mice even in the absence of any adjuvant or immunotherapy. This therapy, therefore, held promise for allowing extended application of radiotherapy in metastatic tumor diseases.

Immunotherapies, especially ICIs, which enhance the immune system's ability to recognize and eradicate cancer cells, have significantly impacted NSCLC patients’ survival. However, not all patients benefit from immunotherapy due to lack of sufficient T‐cell infiltration, impaired activation of immune cells, and immune‐related adverse events,^[^ ^45^ ^]^ while in this study, N@VP combined with IR treatment successfully promoted more mature immune cells’ infiltration, particularly antigen‐specific memory CD8^+^ T cells, by inducing tumor cell pyroptosis, and also enhanced sensitivity to checkpoint‐targeted immunotherapies through upregulating PD‐1 expression, thus realizing dual sensitization of both radiotherapy and immunotherapy in the combined treatment of tumors. Moreover, tumor‐targeted N@VP nanoparticles along with local CDRT further minimized systemic toxicity, showing a more promising modality for the treatment of advanced lung cancer in the future.

## 4 Conclusion

Taken together, in this work, to relieve immunosuppression and realize radiotherapy sensitization, a gelatinase‐responsive verteporfin‐loaded nanoplatform was designed. On the one hand, as described above, this nanocomposite containing VP and gelatinase‐responsive nanocarriers could target VP to tumor tissues and realize excellent antitumor effect no less than SBRT by elevated levels of ROS production and DNA damage under conventional‐dose X‐ray irradiation. On the other hand, this novel X‐PDT strategy could exert a potent immunomodulatory effect far superior to conventional SBRT by pyroptosis‐induced tumor cells’ ICD process, including enhancing the immunogenicity of tumor cells, promoting functional CD4^+^ and CD8^+^ T‐cell infiltration, stimulating DC maturation, and provoking macrophages M1 polarization, ultimately transforming the immunosuppressive microenvironment into an immune‐responsive tumor microenvironment and increasing sensitivity to immunotherapy. Our findings further validated that an effective systemic antitumor immune response was activated in tumor metastasis mouse models after N@VP‐mediated radiosensitization, providing a preferable in situ vaccination based on conventional‐dose radiotherapy to achieve efficient systemic management of tumor distant metastasis.

## 5 Experimental Section

### The Ethical Approval Statement

All animal experiments were carried out in accordance with the guidelines for animal care and approved by Animal Care Committee at Nanjing Drum Tower Hospital, The Affiliated Hospital of Nanjing University Medical School (Nanjing, China) (approval number: 2022AE01015).

### Statistical Analysis

All quantitative data were shown as mean ± SEM (standard error of the mean), *n* ≥ 3. For comparison of two groups, Student's *t*‐test, and for three or more groups’ comparison, one‐way ANOVA and two‐way ANOVA were used, unless otherwise stated. For comparison of groups in mice survival, the log‐rank (Mantel–Cox) test was used. Statistical analysis was performed using GraphPad Prism version 8.0. *P*‐value style: ns represented *p* > 0.05; *p < 0.05; **p < 0.01; ***p < 0.001; and ****p < 0.0001. Flow cytometry data were analyzed by FlowJo X. The ^1^H NMR spectrum was created by MestReNova, and other graphs were created through GraphPad Prism version 8.0 unless otherwise stated.

## Conflict of Interest

The authors declare no conflict of interest.

## Supporting information



Supporting Information

## Data Availability

The data that support the findings of this study are available on request from the corresponding author. The data are not publicly available due to privacy or ethical restrictions.

## References

[1] H. Sung , J. Ferlay , R. L. Siegel , M. Laversanne , I. Soerjomataram , A. Jemal , F. Bray , Ca‐Cancer J. Clin. 2021, 71, 209. 33538338 10.3322/caac.21660

[2] C. Gridelli , A. Rossi , D. P. Carbone , J. Guarize , N. Karachaliou , T. Mok , F. Petrella , L. Spaggiari , R. Rosell , Nat. Rev. Dis. Primers 2015, 1, 15009. 27188576 10.1038/nrdp.2015.9

[3] a) M. Peng , Y. Mo , Y. Wang , P. Wu , Y. Zhang , F. Xiong , C. Guo , X. Wu , Y. Li , X. Li , G. Li , W. Xiong , Z. Zeng , Mol. Cancer 2019, 18, 128; 31443694 10.1186/s12943-019-1055-6 PMC6708248

[4] a) E. B. Golden , A. E. Marciscano , S. C. Formenti , Int. J. Radiat. Oncol., Biol., Phys. 2020, 108, 891; 32800803 10.1016/j.ijrobp.2020.08.023

[5] a) S. Demaria , E. B. Golden , S. C. Formenti , JAMA Oncol. 2015, 1, 1325; 26270858 10.1001/jamaoncol.2015.2756

[6] a) C. Vanpouille‐Box , A. Alard , M. J. Aryankalayil , Y. Sarfraz , J. M. Diamond , R. J. Schneider , G. Inghirami , C. N. Coleman , S. C. Formenti , S. Demaria , Nat. Commun. 2017, 8, 15618; 28598415 10.1038/ncomms15618 PMC5472757

[7] F. G. Herrera , C. Ronet , M. Ochoa de Olza , D. Barras , I. Crespo , M. Andreatta , J. Corria‐Osorio , A. Spill , F. Benedetti , R. Genolet , A. Orcurto , M. Imbimbo , E. Ghisoni , B. Navarro Rodrigo , D. R. Berthold , A. Sarivalasis , K. Zaman , R. Duran , C. Dromain , J. Prior , N. Schaefer , J. Bourhis , G. Dimopoulou , Z. Tsourti , M. Messemaker , T. Smith , S. E. Warren , P. Foukas , S. Rusakiewicz , M. J. Pittet , et al., Cancer Discovery 2022, 12, 108. 34479871 10.1158/2159-8290.CD-21-0003 PMC9401506

[8] a) K. L. Miller , T. D. Shafman , M. S. Anscher , S. M. Zhou , R. W. Clough , J. L. Garst , J. Crawford , J. Rosenman , M. A. Socinski , W. Blackstock , G. S. Sibley , L. B. Marks , Int. J. Radiat. Oncol., Biol., Phys. 2005, 61, 64; 15629595 10.1016/j.ijrobp.2004.02.066

[9] a) P. M. Medin , T. P. Boike , Int. J. Radiat. Oncol., Biol., Phys. 2011, 79, 1302; 21183290 10.1016/j.ijrobp.2010.10.052 PMC3074505

[10] M. Jarosz‐Biej , R. Smolarczyk , T. Cichon , N. Kulach , Int. J. Mol. Sci. 2019, 20, 3212. 31261963 10.3390/ijms20133212 PMC6650939

[11] J. M. Houle , A. Strong , J. Clin. Pharmacol. 2002, 42, 547. 12017349 10.1177/00912700222011607

[12] M. T. Huggett , M. Jermyn , A. Gillams , R. Illing , S. Mosse , M. Novelli , E. Kent , S. G. Bown , T. Hasan , B. W. Pogue , S. P. Pereira , Br. J. Cancer 2014, 110, 1698. 24569464 10.1038/bjc.2014.95 PMC3974098

[13] a) S. Clement , W. Deng , E. Camilleri , B. C. Wilson , E. M. Goldys , Sci. Rep. 2016, 6, 19954; 26818819 10.1038/srep19954 PMC4730142

[14] W. M. Bonner , C. E. Redon , J. S. Dickey , A. J. Nakamura , O. A. Sedelnikova , S. Solier , Y. Pommier , Nat. Rev. Cancer 2008, 8, 957. 19005492 10.1038/nrc2523 PMC3094856

[15] a) L. Liang , H. Cen , J. Huang , A. Qin , W. Xu , S. Wang , Z. Chen , L. Tan , Q. Zhang , X. Yu , X. Yang , L. Zhang , Mol. Cancer 2022, 21, 186; 36171576 10.1186/s12943-022-01651-4 PMC9516831

[16] a) G. She , J. C. Du , W. Wu , T. T. Pu , Y. Zhang , R. Y. Bai , Y. Zhang , Z. D. Pang , H. F. Wang , Y. J. Ren , J. Sadoshima , X. L. Deng , X. J. Du , Theranostics 2023, 13, 560; 36632235 10.7150/thno.79227 PMC9830444

[17] I. Raphael , S. Nalawade , T. N. Eagar , T. G. Forsthuber , Cytokine 2015, 74, 5. 25458968 10.1016/j.cyto.2014.09.011 PMC4416069

[18] Q. Shang , X. Yu , Q. Sun , H. Li , C. Sun , L. Liu , Biomed. Pharmacother. 2023, 170, 115976. 38043444 10.1016/j.biopha.2023.115976

[19] A. Tanaka , S. Sakaguchi , Cell Res. 2017, 27, 109. 27995907 10.1038/cr.2016.151 PMC5223231

[20] J. Fucikova , O. Kepp , L. Kasikova , G. Petroni , T. Yamazaki , P. Liu , L. Zhao , R. Spisek , G. Kroemer , L. Galluzzi , Cell Death Dis. 2020, 11, 1013. 33243969 10.1038/s41419-020-03221-2 PMC7691519

[21] D. V. Krysko , A. D. Garg , A. Kaczmarek , O. Krysko , P. Agostinis , P. Vandenabeele , Nat. Rev. Cancer 2012, 12, 860. 23151605 10.1038/nrc3380

[22] H. Jiang , N. Kong , Z. Liu , S. C. West , Y. W. Chan , Adv. Sci. 2023, 10, 2204388. 10.1002/advs.202204388 PMC10131833 36825683

[23] a) T. Zhang , D. M. Wu , P. W. Luo , T. Liu , R. Han , S. H. Deng , M. He , Y. Y. Zhao , Y. Xu , Cell Death Dis. 2022, 13, 167; 35190532 10.1038/s41419-022-04561-x PMC8861163

[24] a) Y. Lu , F. Xu , Y. Wang , C. Shi , Y. Sha , G. He , Q. Yao , K. Shao , W. Sun , J. Du , J. Fan , X. Peng , Biomaterials 2021, 278, 121167; 34624752 10.1016/j.biomaterials.2021.121167

[25] P. Chen , Y. Liu , Y. Wen , C. Zhou , Cancer Commun. 2022, 42, 937. 10.1002/cac2.12359 PMC9558689 36075878

[26] a) N. A. Rizvi , J. Mazieres , D. Planchard , T. E. Stinchcombe , G. K. Dy , S. J. Antonia , L. Horn , H. Lena , E. Minenza , B. Mennecier , G. A. Otterson , L. T. Campos , D. R. Gandara , B. P. Levy , S. G. Nair , G. Zalcman , J. Wolf , P. J. Souquet , E. Baldini , F. Cappuzzo , C. Chouaid , A. Dowlati , R. Sanborn , A. Lopez‐Chavez , C. Grohe , R. M. Huber , C. T. Harbison , C. Baudelet , B. J. Lestini , S. S. Ramalingam , Lancet Oncol. 2015, 16, 257; 25704439 10.1016/S1470-2045(15)70054-9 PMC5726228

[27] a) M. Enamorado , S. Iborra , E. Priego , F. J. Cueto , J. A. Quintana , S. Martinez‐Cano , E. Mejias‐Perez , M. Esteban , I. Melero , A. Hidalgo , D. Sancho , Nat. Commun. 2017, 8, 16073; 28714465 10.1038/ncomms16073 PMC5520051

[28] a) R. S. Akondy , M. Fitch , S. Edupuganti , S. Yang , H. T. Kissick , K. W. Li , B. A. Youngblood , H. A. Abdelsamed , D. J. McGuire , K. W. Cohen , G. Alexe , S. Nagar , M. M. McCausland , S. Gupta , P. Tata , W. N. Haining , M. J. McElrath , D. Zhang , B. Hu , W. J. Greenleaf , J. J. Goronzy , M. J. Mulligan , M. Hellerstein , R. Ahmed , Nature 2017, 552, 362; 29236685 10.1038/nature24633 PMC6037316

[29] D. M. Cannon , M. P. Mehta , J. B. Adkison , D. Khuntia , A. M. Traynor , W. A. Tome , R. J. Chappell , R. Tolakanahalli , P. Mohindra , S. M. Bentzen , G. M. Cannon , J. Clin. Oncol. 2013, 31, 4343. 24145340 10.1200/JCO.2013.51.5353 PMC3837093

[30] a) M. Gao , C. Liang , X. Song , Q. Chen , Q. Jin , C. Wang , Z. Liu , Adv. Mater. 2017, 29, 1701429; 10.1002/adma.201701429 28722140

[31] P. F. Nguyen‐Tan , Q. Zhang , K. K. Ang , R. S. Weber , D. I. Rosenthal , D. Soulieres , H. Kim , C. Silverman , A. Raben , T. J. Galloway , A. Fortin , E. Gore , W. H. Westra , C. H. Chung , R. C. Jordan , M. L. Gillison , M. List , Q. T. Le , J. Clin. Oncol. 2014, 32, 3858. 25366680 10.1200/JCO.2014.55.3925 PMC4239304

[32] M. W. Knitz , T. E. Bickett , L. B. Darragh , A. J. Oweida , S. Bhatia , B. Van Court , S. Bhuvane , M. Piper , J. Gadwa , A. C. Mueller , D. Nguyen , V. Nangia , D. G. Osborne , X. Bai , S. E. Ferrara , M. K. Boss , A. Goodspeed , M. A. Burchill , B. A. J. Tamburini , E. D. Chan , C. R. Pickering , E. T. Clambey , S. D. Karam , J. Immunother. Cancer 2021, 9, 001955. 10.1136/jitc-2021-002585 PMC8016081 33789881

[33] a) X. Liu , S. Xia , Z. Zhang , H. Wu , J. Lieberman , Nat. Rev. Drug Discovery 2021, 20, 384; 33692549 10.1038/s41573-021-00154-z PMC7944254

[34] a) L. Aymeric , L. Apetoh , F. Ghiringhelli , A. Tesniere , I. Martins , G. Kroemer , M. J. Smyth , L. Zitvogel , Cancer Res. 2010, 70, 855; 20086177 10.1158/0008-5472.CAN-09-3566

[35] Q. Gao , F. Li , S. Wang , Z. Shen , S. Cheng , Y. Ping , G. Qin , X. Chen , L. Yang , L. Cao , S. Liu , B. Zhang , L. Wang , Y. Sun , Y. Zhang , Cell. Immunol. 2019, 343, 103850. 30153900 10.1016/j.cellimm.2018.08.011

[36] a) Z. Li , W. J. Fu , X. Q. Chen , S. Wang , R. S. Deng , X. P. Tang , K. D. Yang , Q. Niu , H. Zhou , Q. R. Li , Y. Lin , M. Liang , S. S. Li , Y. F. Ping , X. D. Liu , X. W. Bian , X. H. Yao , J. Exp. Clin. Cancer Res. 2022, 41, 74; 35193644 10.1186/s13046-022-02291-8 PMC8862393

[37] M. Di , J. Miao , Q. Pan , Z. Wu , B. Chen , M. Wang , J. Zhao , H. Huang , J. Bai , Q. Wang , Y. Tang , Y. Li , J. He , T. Xiang , D. Weng , L. Wang , J. Xia , C. Zhao , J. Exp. Clin. Cancer Res. 2022, 41, 328. 36411454 10.1186/s13046-022-02533-9 PMC9677691

[38] J. Borst , T. Ahrends , N. Babala , C. J. M. Melief , W. Kastenmuller , Nat. Rev. Immunol. 2018, 18, 635. 30057419 10.1038/s41577-018-0044-0

[39] a) C. D. Mills , K. Kincaid , J. M. Alt , M. J. Heilman , A. M. Hill , J. Immunol. 2000, 164, 6166; 28923981 10.4049/jimmunol.1701141

[40] D. M. Mosser , J. P. Edwards , Nat. Rev. Immunol. 2008, 8, 958. 19029990 10.1038/nri2448 PMC2724991

[41] a) Y. Shen , C. Lu , Z. Song , C. Qiao , J. Wang , J. Chen , C. Zhang , X. Zeng , Z. Ma , T. Chen , X. Li , A. Lin , J. Guo , J. Wang , Z. Cai , Nat. Commun. 2022, 13, 3419; 35701426 10.1038/s41467-022-31141-6 PMC9198048

[42] Y. Abuodeh , P. Venkat , S. Kim , Curr. Probl. Cancer 2016, 40, 25. 26582738 10.1016/j.currproblcancer.2015.10.001

[43] a) M. J. Frank , P. M. Reagan , N. L. Bartlett , L. I. Gordon , J. W. Friedberg , D. K. Czerwinski , S. R. Long , R. T. Hoppe , R. Janssen , A. F. Candia , R. L. Coffman , R. Levy , Cancer Discovery 2018, 8, 1258; 30154192 10.1158/2159-8290.CD-18-0743 PMC6171524

[44] A. Levy , C. Massard , J. C. Soria , E. Deutsch , Eur. J. Cancer 2016, 68, 156. 27764686 10.1016/j.ejca.2016.09.013

[45] a) K. Haratani , H. Hayashi , Y. Chiba , K. Kudo , K. Yonesaka , R. Kato , H. Kaneda , Y. Hasegawa , K. Tanaka , M. Takeda , K. Nakagawa , JAMA Oncol. 2018, 4, 374; 28975219 10.1001/jamaoncol.2017.2925 PMC6583041

