# Supplementary material for: Verteporfin‐Mediated In Situ Nanovaccine Based on Local Conventional‐Dose Hypofractionated Radiotherapy Enhances Antitumor and Immunomodulatory Effect
Source: Adv Sci (Weinh). 2025 Apr 15;12(20):2413387. doi: 10.1002/advs.202413387 (PMC12120762; doi:10.1002/advs.202413387)
Supplement: Supplementary file 1 — Supporting Information [file ADVS-12-2413387-s001.docx]

Supporting information for

**Verteporfin-mediated in situ nano-vaccine based on local conventional-dose hypofractionated radiotherapy enhances antitumor and immunomodulatory effect**

Zhifan Zhang, Lin Li, Yuchen Ge, Anni Chen, Shanchao Diao, Yueling Yang, Qianyue Chen, Yingling Zhou, Jie Shao, Fanyan Meng, Lixia Yu, Manman Tian, Xiaoping Qian, Zhaoyu Lin, Chen Xie, Baorui Liu* and Rutian Li*

**Corresponding author:** Rutian Li (Email: rutianli@nju.edu.cn);

Baorui Liu (Email: baoruiliu@nju.edu.cn)

**Supporting Information**

Methods

Figure S1- Figure S28

Methods

**Materials**

Methoxypolyethyleneglycol-NHS (mPEG-NHS, MW: 5, 000) and polycaprolactone-NH_2_ (PCL-NH_2_, MW: 10, 000) were obtained from Xi'anruixi Biological Technology Co., Ltd (Xi'an, China). The substrate peptide of gelatinase (PVGLIG, Pro-Val-Gly-Leu-Ile-Gly) was synthesized by Shanghai HD Biosciences Company (Shanghai, China). All other reagents including verteporfin were purchased from Aladdin (Shanghai, China) unless otherwise indicated. Mouse PD-1 antibody (anti-mPD-1, αP) was provided by Selleck (Clone: RMP1-14).

**Cell lines**

Mouse Lewis lung carcinoma (LLC) cells were obtained from Cell Bank of Shanghai Institute of Biochemistry and Cell Biology and cultured in Roswell Park Memorial Institute (RPMI) 1640 (Gorning, USA) supplemented with 10% fetal bovine serum (FBS) (Gibco, USA) at 37 °C and 5% CO_2_.

**X-Irradiation**

For X-irradiation, the cells maintained in the dishes were exposed to different doses of X-irradiation of using 6 MeV (million electron volts) beam that was generated by a clinical linear accelerator (Elekta Precise, Sweden). While X-irradiation was applied to tumors of mice, with the rest of body covered by lead. All irradiations were carried out at room temperature in the radiotherapy department of Nanjing Drum Tower Hospital, the Affiliated Hospital of Nanjing University Medical School (Nanjing, China).

**Mice**

6–8 weeks old female C57BL/6 mice were kept under SPF conditions at 21–25 °C, with humidity at 50–70% and a 12h light/dark cycle. The mice had access to adequate food and water ad libitum at all times. C57BL/6 female mice were purchased from the Beijing Vital River Laboratory Animal Technology Co., Ltd. (Beijing, China) and adaptive feeding for a week in the laboratory before the experiment. All animal experiments were carried out in accordance with the guidelines for animal care and approved by Animal Care Committee at Nanjing Drum Tower Hospital, The Affiliated Hospital of Nanjing University Medical School (Nanjing, China) (approval number: 2022AE01015).

**Singlet oxygen generation test**

To quantify the amount of ^1^O_2_ generated from VP samples with different molar ratios, the black 96-well plates with 200 µL of VP suspension and 1.6 µL of Singlet oxygen sensor green (SOSG, Thermo Fisher, Cat. S36002) fluorescent probe (0.5 mM) in each well were exposed to different dosage of X-ray radiation (6 MeV LINAC) and each group was set up with 4-6 replicate wells. Before and after irradiation, the SOSG fluorescence in each well at 525 nm upon 488 nm excitation was recorded using a Varioskan Lux microplate reader (Thermo Fisher Scientific). The increase of SOSG fluorescence after irradiation was then calculated. The whole experiment was carried out on ice in the dark.

**H2AX phosphorylation test using flow cytometry**

DNA damage was evaluated by the levels of γ-H2AX, a DNA break-associated protein. LLC cells were seeded and cultured in a 24-well plate (1 × 10^5^ per well) until logarithmic growth phase, at which time cells were incubated with serum-free medium with or without verteporfin for 3 hours in the dark, and then the serum-free medium was replaced with fresh serum-containing and verteporfin-free medium prior to X-ray radiation. Cells were harvested by trypsin digestion one hour after irradiation and washed twice in phosphate-buffered saline (PBS). Pre-cooled 70% ethanol was added to cell precipitate drop by drop while vortexing and then stored at -20°C overnight. Cells were collected by centrifugation and washed twice in pre-cold FBS-PBS (PBS containing 2% FBS). For antibody treatment, cells were resuspended in 100 μl of FBS-PBS containing 5 ul of FITC anti-H2AX Phospho (Ser139) Antibody (BioLegend, Cat. 613404) and incubated in the dark at room temperature for 60 min. Unbound antibody was washed off with FBS-PBS prior to flow analysis. Expression of γ-H2AX was measured using BD Accuri C6 (BD Bioscience, USA) and analyzed by FlowJo software.

**Proliferative capacity assay**

Long-term influence on proliferative capacity of the LLC cells after different treatment was evaluated by clonogenic assay. Cells were counted and planted in dishes. As described above, X-rays were administered to cells after 3 hours of incubation with serum-free medium with or without VP/N@VP. Following drug and varied doses of X-ray irradiation treatment, cells were incubated for 10 days. On day 10, cells were ﬁxed with 95% ethyl alcohol at room temperature for 20 min. Then, cells were stained with crystal violet solution (C0121, Beyotime, China) for 15 min, followed by washing twice with PBS. Colonies containing ≥ 50 cells were counted under the microscope and the survival rate was calculated.

$$\text{Survival rate = }\frac{\text{Number of colonies}}{\text{Number of cells inoculated}}\text{ }$$

**Synthesis of N@VP nanoparticles**

First of all, the mPEG-peptide (mPEG-PVGLIG) was synthesized by dissolving the mPEG-NHS (200 mg) and gelatinase-sensitive peptide PVGLIG (26 mg) in 3 mL dimethyl formamide (DMF) containing 3% triethylamine and then stirring at 300 rpm for 3 hours at room temperature. Then, the solution was dialyzed using 3500-Da MWCO dialysis bag for 3 days to remove the unconjugated peptides. Next, the purified mPEG-PVGLIG conjugates (0.02 mol) were dehydrated to powder and then mixed with PCL-NH_2_ (0.02 mol), 4-dimethylaminopyridine (DMAP, 0.03 mol), 1-ethyl-3-(3-dimethyl aminopropyl) carbodiimide (EDC, 0.02 mol) and N-Hydroxysuccinimide (NHS, 0.02 mol) in 3 ml DMF. These reaction mixtures were stirred for 24 hours at 30℃, then the generated mPEG-PVGLIG-PCL copolymer was purified by dialysis (MWCO 14kDa) for 24 hours. At last, the dialyzed samples were lyophilized into powder and stored at 4℃ for further use.

Verteporfin-loaded nanoparticles (N@VP) were prepared by single o/w emulsion and solvent evaporation method (Figure S4, Supporting Information). Briefly, mPEG-PVGLIG-PCL copolymer (25 mg) and VP (5 mg) were dissolved in the 2.5 ml methylene chloride (DCM). The mixture was emulsified in 7.5 ml 5% polyvinyl alcohol (PVA) aqueous solution by sonication (XL2000, Misonix, USA) for 3 min (65 W) to obtain an o/w emulsion. Then, this emulsion was diluted in 20 ml 1% PVA aqueous solution and stirring for 3 hours in the draught cupboard to remove DCM. The obtained emulsion was filtered and centrifugated (20000 rpm, 40 min) to collect N@VP microspheres without non-incorporated VP, and finally the collected microspheres were resuspended in 5.19 mL PBS to prepare N@VP solution. The blank nanoparticles (BNP) were prepared in the same procedure without adding verteporfin.

**Nanoparticle characterization**

The matrix-assisted laser desorption/ionization time-of-flight (MALDI-TOF) mass spectra of mPEG-NHS and mPEG-PVGLIG were measured by a Bruker Autoflex TOF/TOF Spectrometer. Proton nuclear magnetic resonance (^1^H NMR) of mPEG-PVGLIG, PCL-NH_2_ and mPEG-PVGLIG-PCL were conducted on a Bruker Ultra Shield Plus 400 MHz Spectrometer. CDCl_3_ was used as the solvent. The morphology and structure of N@VP nanoparticles were characterized by a transmission electron microscope (TEM, JEM-100 S, JEOL, Japan). Size distribution and zeta potential of N@VP nanoparticles were measured by dynamic light scattering (DLS) and the laser Doppler anemometry (Brookhaven Instruments Corporation, USA) at 25 °C with a 90° scattering angle. The encapsulation efficiency of VP was analyzed by HPLC (at 410 nm). The mobile phase was composed of 0.08 M (NH_4_)_2_SO_4_: acetonitrile: tetrahydrofuran: acetic acid (52: 28: 28: 5) with a flow rate of 1 ml min^-1^.

**Nanoparticle cellular uptake**

LLC cells were seeded in a 35 mm confocal dish (1 × 10^5^ per dish) and treated in the logarithmic growth phase. After incubating with serum-free medium containing free-VP or N@VP for 3 hours in the dark, the cell culture medium was replaced by a fresh one. Then, the cellular uptake capacity was visualized via confocal laser scanning microscopy (Leica, Germany). Fluorescence of VP was measured at 696 nm with excitation at 405 nm.

**Gelatinase-responsive release of N@VP**

To explore the gelatinase-responsive release of N@VP, a solution of N@VP with or without type Ⅳ collagenase (Cat. 17104019, Gibco) was enclosed in a dialysis bag (molecular weight cut off: 14k Da) and immersed in 5 ml of PBS with gentle agitation at 37°C for 24 h. The concentrations of VP (Excitation: 405 nm, Emission: 696 nm) in the dialysate at various time points (0, 4, 8, 12, 18, 24 hours) were measured using a Varioskan Lux microplate reader (Thermo Fisher Scientific).

**In vitro cytotoxicity assay**

LLC cells and HUVEC cells were seeded into 96-well plates at a density of 5000 cells per well and cultured for 24 hours. Subsequently, different concentrations of VP-loaded nanoparticles (N@VP), free VP, and blank nanoparticles (BNP) were added to the respective wells. After an additional incubation period of 24 hours, CCK8 reagent was added to each well and incubated for 1-2 hours at 37 °C. The absorbance of the cultures was then measured at a wavelength of 450 nm using a Varioskan Lux microplate reader (Thermo Fisher Scientific).

**In vivo tumor-targeting of N@VP nanoparticles**

Using 1,1′-dioctadecyl-3,3,3′,3′-tetramethylindotricarbocyanine iodide (DiR) as a more stable and brighter near-infrared fluorescence (NIR) fluorescent dye to study the tumor-targeting ability of mPEG-PVGLIG-PCL-based nanoparticles. DiR-loaded nanoparticles (N@DiR) was prepared exactly the same as VP-loaded nanoparticles, except that VP was replaced by DiR. The BNP, VP (5 mg kg^-1^), N@VP (VP: 5 mg kg^-1^) and N@DiR solution were tail vein injected into LLC bearing C57BL/6 female mice, and in vivo distribution of nanoparticles was studied by real-time NIR imaging using CRi Maestro In Vivo Imaging System (Cambridge Research & Instrumentation, Massachusetts, USA). The NIR of VP was imaged at 790 nm with excitation at 680 nm and the NIR of DiR was imaged at 740 nm with excitation at 790 nm.

**Intracellular ROS detection**

In consideration of failure in cell penetration of SOSG, we employ a cell-penetrating fluorescent probe of ROS called 2′,7’- Dichlorodihydrofluorescein diacetate (DCFH-DA) (Sigma-Aldrich, St. Louis, MO, Cat. 4091-99-0) for intracellular ROS detection. 2 mg of DCFH-DA was dissolved in 400 μL dimethylsulfoxide (10mM) to prepare DCFH-DA stock solution. LLC cells were seeded in a 35 mm confocal dish (1 × 10^5^ per dish) and treated in the logarithmic growth phase with serum-free medium with or without N@VP for 3 hours prior to X-ray radiation. Cells in each confocal dish were washed twice with PBS immediately after irradiation and incubated with 1 mL PBS containing 1 uL DCFH-DA stock solution at 37℃ for 20 min. After washing with PBS, cells were stained with Hoechst (Beyotime, Shanghai, China). Then, cells were imaged on the confocal laser scanning microscopy (Leica, Germany) with green and blue fluorescence acquired sequentially. Quantitation of mean fluorescence intensity was analyzed using ImageJ software.

**Immunofluorescence staining of γ-H2AX**

DNA damage was detected by immunofluorescence staining of γ-H2AX using the DNA Damage Assay Kit by γ-H2AX Immunofluorescence (C2035S; Beyotime, Shanghai, China). Treatment of cells was done as before. Briefly, logarithmically growing LLC cells in a 35 mm confocal dish were treated with serum-free medium with or without N@VP for 3 hours. After X-ray irradiation (2 Gy or 8 Gy), the cells in dishes were washed twice with cold PBS and fixed with immunostaining fixative solution for 15 min. After washing with immunostaining washing buffer containing 0.5% Triton X-100, cells were blocked at 4°C for 20 min using QuickBlock™ Blocking Buffer for Immunol Staining. For the primary antibody treatment, cells were incubated with an anti-γ-H2AX primary antibody (rabbit monoclonal; 1:200) at room temperature for 2 hours, followed by washing with immunostaining washing buffer. Then LLC cells were incubated with an anti-rabbit FITC-conjugated secondary antibody (1:200) for 2 hours at room temperature. DAPI was used for cell nuclei staining. Fluorescent images were obtained using the confocal laser scanning microscopy (Leica, Germany).

**Extracellular ATP assay**

Extracellular release of ATP was determined using an Enhanced ATP Assay Kit (Beyotime, Shanghai, China). LLC cells reached logarithmic growth phase in a 24-well plate, followed by incubation with serum-free medium with or without N@VP for 3 hours in the dark. And then the culture medium was replaced with a fresh one prior to X-ray radiation. Cell-free supernatants were collected 6 hours after X-ray irradiation to determine the extracellular ATP levels. Detecting solution was added to a 96-well plate and left undisturbed for 5 min. The collected supernatants were then added to the wells and mixed fast before measuring the luminescence signals by the Varioskan Lux microplate reader (Thermo Fisher Scientific).

**RNA sequencing and analysis**

LLC cells collected from Con, VP, IR and VP + IR groups (3 samples per group) were pooled into one replicate set each of Con, VP, IR and VP + IR cells. Total RNA from collected cells in each group was isolated using TRIzol reagent (Thermo Fisher Scientific, Waltham, MA, USA). Services including library construction, library quality inspection and RNA sequencing were provided by GENEWIZ. In brief, RNA libraries were prepared from Total RNA (1 μg) of each sample according to standard Illumina protocols. Transcriptome sequencing was subsequently conducted on the Illumina HiSeq platform. The raw image data of sequencing results were identified by image base calling using Bcl2fastq software (version 2.17.1.14). After preliminary quality analysis by built-in software of Illumina instrument, the raw data of sequenced samples (Pass Filter Data) was obtained and stored in FASTQ file format. The quality of FASTQ files was analyzed by the software FastQC (version 0.10.1) and the raw data were filtered using Cutadapt (version 1.9.1) to remove low-quality reads, Illumina adapter contaminations, higher N rate sequences and reads < 75 bp.

P values were adjusted by multiple hypothesis testing based on the Benjamini-Hochberg method and calculated to q values (p.adjust), in order to better control false-positive rates. Analysis of differential genes (q value < 0.05) between the Con and the VP + IR group was performed using DESeq, and significantly upregulated and downregulated genes (absolute value of logFC ≥ 2 and -log10qvalue > 100) were labeled in the volcano plot. GO and KEGG enrichment analysis used GOSeq and the database (http://en.wikipedia.org/wiki/KEGG) respectively. GSEA was performed based on the hallmark gene set (mh.all.v2023.1.Mm.symbols.gmt) (https://www.gsea-msigdb.org/gsea/msigdb). All data were analyzed using the R software (version 4.2.2).

**Construction and therapy of Lewis lung carcinoma mouse models**

Female C57BL/6 mice (6~8 weeks old) were challenged with LLC cells (100 μl, 1 × 10^6^ cells per mouse) by subcutaneous injection into the left groin to establish Lewis lung carcinoma mouse models. Tumor volumes (0.5×length × (width)^2^) were measured using a caliper and calculated every 2 days. Mice were randomly assigned to different groups (n=5~6) and received their respective treatments when tumor volumes reached approximately 100 mm^3^. Mice were treated with PBS, 2 Gy irradiation, N@VP (5 mg kg^-1^ VP i.v.), N@VP + 2 Gy (5 mg kg^-1^ VP i.v. 16 hours before irradiation) and 8 Gy irradiation. Animals were euthanized if they suffered from poor quality of life (ascites, emaciation and the like) or when tumor volume reached 2000 mm^3^. For in vivo safety studies, the major organs and tumors were excised at 10 days and 6 hours respectively after the treatment and fixed with 10% neutral-buffered formalin for hematoxylin-eosin (H&E) staining. Besides, on the 3^rd^ day after the last treatment, blood serum (3 samples per group) was collected for biochemical index detection, including indicators of liver function (aspartate transaminase [AST], alanine transaminase [ALT], lactate dehydrogenase [LDH]), renal function (creatinine [CREA], UREA) and cardiac function (creatine kinase [CK], creatine kinase isoenzymes [CK-MB], α-hydroxybutyrate dehydrogenase [α-HBDH]).

To explore the abscopal effect of treatment, the secondary LLC tumor (100 μl, 1 × 10^6^ cells per mouse) was subcutaneously injected in the right underarm of the LLC-bearing female C57BL/6 mice at day 12 after the primary tumor implantation, in order to simulate distant metastasis. Treatment was started at day 4 after the distant tumor implantation (6 mice for each group) and the CDRT (2 Gy/fraction) and HDRT (8 Gy/fraction) were performed at day 4, day 7 and day 13. X-ray irradiation was only delivered to the first tumor (in situ tumor), and during the treatment, distant tumor growth of mice was monitored every 2 days.

To evaluate the immunotherapy (ICIs) sensitization, we firstly established Lewis lung carcinoma mouse models as indicated above. And the LLC-bearing female C57BL/6 mice subsequently received various treatments (n=6 for each group): PBS, mouse PD-1 antibody (αP, 10 mg kg^-1^), αP plus 2 Gy irradiation (αP i.v. 16 h before RT), N@VP + 2 Gy and N@VP/αP + 2 Gy irradiation (5 mg kg^-1^ VP and 10 mg kg^-1^ αP, N@VP/αP i.v. 16 h before RT). Tumor volumes and mouse survival were monitored until the mouse was sacrificed.

**Immunohistochemical staining**

Mouse/human tumors and mouse organ tissues were fixed with 4% paraformaldehyde solution, embedded in paraffin blocks and prepared as 4 μm sections. H&E staining was done in mouse organ tissue and Lewis lung carcinoma sections for toxicity analysis. For MMP2/MMP9 staining, endogenous peroxidase blocking was performed using a 1% H_2_O_2_ in methyl alcohol for 30 min, followed by microwave-mediated antigen retrieval in 10 mM citrate buffer (pH 6) for 30 min. After washing, the sections underwent a blocking step with 5% bovine serum albumin in PBS for 1 h. Additionally, all slides were incubated with MMP2 rabbit polyclonal antibody (Proteintech, China; Cat. 10373-2-AP) or MMP9 rabbit primary antibody (Abclonal, China; Cat. A25299) at a consistent concentration (1:200) overnight at 4℃. After washing three times with PBS, slides were incubated with a horseradish peroxidase (HRP)-labeled anti-rabbit secondary antibody (Beyotime, Cat. A0208; 1:50) at room temperature for 30 min. The sections were then processed using 0.05% 3,3´-diaminobenzidine tetrahydrochloride and counterstained with modified harris hematoxylin. Finally, the sections were observed and photographed using a ZEISS microscope. The percentage of MMP2-positive area and MMP9-positive area were measured using ImageJ software.

**Secretion of IL-1β and IL-18**

LLC cells were incubated in 24-well plates for 24 hours, followed by different treatments. The supernatant was collected 12 hours later and centrifuged (300g, 10 min) to remove cellular debris. Then, the levels of IL-1β and IL-18 in the supernatant were measured using Mouse IL-1β High Sensitivity ELISA kit (MULTI SCIENCES, Cat. EK201BHS) and Mouse interleukin 18 (IL-18) ELISA kit (UpingBio, Cat. SYP-M0193).

**Western blot analysis**

Tumor tissues were isolated from mice at the 6^th^ hour after various treatment and trimmed with scissors. Then the tumors were flash-frozen in the liquid nitrogen, ground into paste and lysed on ice with RIPA buffer (Thermo Fisher Scientific) containing 1% protease and phosphatase inhibitor cocktail (Thermo Scientific) for 30 min. Total protein was extracted and quantified by BCA Protein Quantification Kit (Vazyme, Nanjing, China). 20 μg total protein of each sample was separated in a 12% Tris-glycine SDS-PAGE gel and electroblotted onto PVDF membranes (Millipore). The membranes were blocked in 5% nonfat milk for 2 hours and incubated with primary antibodies at 4 ℃ overnight. Primary antibodies included NLRP3 (Beyotime, Cat. AF2155), Caspase1(Thermo Fisher Scientific, Cat. 14-9832-82), cleaved Caspase1 (Proteintech, Wuhan, China; Cat. 81482-1-RR), GSDMD, cleaved GSDMD (Abcam, Cat. ab219800) and HMGB1 (Beyotime, Cat. AF0180). Blots were then incubated with HRP-conjugated secondary antibody (Beyotime, Cat. A0354; 1:5000) for 2 hours at room temperature. All primary antibodies were diluted 1:1000.

**In vivo immunological analysis using flow cytometry**

Spleens, tumor-draining lymph nodes and tumor tissues were isolated from mice in each group at the end of experiment. Single-cell suspension from the spleen and lymph nodes was obtained through 40-mm nylon cell strainers (Biosharp) while tumor tissues were chopped, and then digested with serum-free RMPI-1640 containing 1 mg mL^-1^ collagenase IV (Sigma-Aldrich) for 2 hours at 37°C. After terminating the digestion by addition of growth medium, cells were filtered through 40-mm nylon cell strainers. Red blood cells in single-cell suspensions of spleen and tumor samples were then removed using red blood cell lysis buffer (Biosharp). The obtained single-cell suspensions without red blood cells were stained with antibodies (diluted 1:20 with PBS) for 20 min at room temperature in the dark, and then washed twice with PBS before analysis using BD Accuri C6 PLUS Flow Cytometry (BD Biosciences). All raw data were further analyzed using FlowJo X software.

Antibodies used for flow cytometry including purified anti-mouse CD16/32 antibody (Clone: 93; Cat. 101302), anti-mouse CD3 (FITC, Clone: 17A2; Cat. 100204), anti-mouse CD4 (APC, Clone: RM4-4; 116014), anti-mouse IFN-γ (PE/Cyanine7, Clone: W18272D; Cat. 505826), anti-mouse IL-4 (PE, Clone: 11B11; Cat. 504104), anti-mouse CD4 (PE/Cyanine7, Clone: GK1.5; Cat. 100422), anti-mouse CD25 (PE, Clone: A18246A; Cat. 113704), anti-mouse FoxP3 (Alexa Fluor® 647, Clone: MF-14; Cat. 126408), anti-mouse CD8 (APC, Clone: 53-6.7; Cat. 100712), anti-mouse CD11C (FITC, Clone: N418; Cat. 117306), anti-mouse CD80 (APC, Clone: 16-10A1; Cat. 104714), anti-mouse CD86 (PE/Cyanine7, Clone: GL-1; Cat. 105014), anti-mouse I-A/I-E (MHC II; PE, Clone: M5/114.15.2; Cat. 107608), anti-mouse CD11b (FITC, Clone: M1/70; Cat. 101206), anti-mouse F4/80 (PE/Cyanine7, Clone: BM8; Cat. 123114), anti-mouse CD86 (PE, Clone: A17199A; Cat. 159204), anti-mouse CD206 (APC, Clone: C068C2; Cat. 141708), anti-mouse CD8 (PerCP/Cyanine5.5, Clone: 53-6.7; Cat. 100734), anti-mouse CD44 (PE, Clone: IM7; Cat. 103024), anti-mouse CD62L (PE/Cyanine7, Clone: W18021D; Cat. 161214), and anti-mouse PD-1 (PerCP/Cyanine5.5, Clone: 29F.1A12; Cat. 135208) were all purchased from Biolegend.

**Specific tumor cell killing capacity of splenic T cells**

Single-cell suspension from the spleen in each group was prepared at the end of the experiment as described above and incubated with RPMI medium 1640 containing 10% FBS in 10 cm dishes at 37°C and 5% CO_2_ for 2 hours. Then the suspended T cells were collected, counted and resuspended in fresh RPMI growth medium for further use. Target cells (LLC) were harvested in the logarithmic growth phase and incubated with CSFE-FITC (Vazyme) for 15 min in PBS at 37°C. CFSE-labeled LLC cells were then incubated with effector cells (suspended T cells) in a 96-well round-bottom plate for 6h at 37°C, 5% CO_2_. The effector-to-target (E/T) ratio was set to 12.5: 1, 25: 1, and 50: 1. After incubation, cells were collected and stained with PI (Vazyme) to distinguish dead target cells (CFSE^+^, PI^+^) from living cells (CFSE^+^, PI^−^). Cytotoxicity assays were performed via BD Accuri C6 PLUS (BD Biosciences). All raw data were analyzed using FlowJo X software. Culture supernatants were also collected after 6 hours of incubation for cytokine measurement as described below.

**Cytokine measurement**

Cytokine levels in the above-mentioned supernatants were measured using LEGENDplex™ MU Th1/Th2 Panel (8-plex) w/ VbP V03 (BioLegend, USA) according to the manufacturer’s instructions.

Tumors from each treatment group were excised, weighed, and flash-frozen in liquid nitrogen. Then, tumor samples were lysed in RIPA buffer containing 1% protlytic protease and phosphatase inhibitor cocktail (P002, New Cell & Molecular Biotech Co., Ltd, China) at a ratio of 1 mL buffer per 100 mg tissues for half an hour on the ice, followed by centrifugation (12,000 ×g, 10 min, 4°C). The protein concentrations of supernatant were determined by BCA to be 4 μg μL^-1^. Then, 25 μL of diluted samples (100 μg total protein per sample) were analyzed using LEGENDplex™ MU Th Cytokine Panel. The cytokines were detected via BD Accuri C6 PLUS (BD Biosciences) and concentrations were calculated by LEGENDplex™ software.

**Statistical analysis**

All quantitative data are shown as mean ± SEM, n≥3. For comparisons of two groups, Student's t-test, and for three or more groups comparisons, one-way ANOVA and two-way ANOVA were used, unless otherwise stated. For comparisons of groups in mice survival, log-rank (Mantel-Cox) test was used. Statistical analysis was performed using GraphPad Prism version 8.0. P-value style: ns represented p > 0.05; *p < 0.05; **p < 0.01; ***p < 0.001; ****p < 0.0001. Flow cytometry data were analyzed by FlowJo X. The ^1^H NMR spectrum was created by MestReNova and other graphs were created through GraphPad Prism version 8.0 unless otherwise stated.


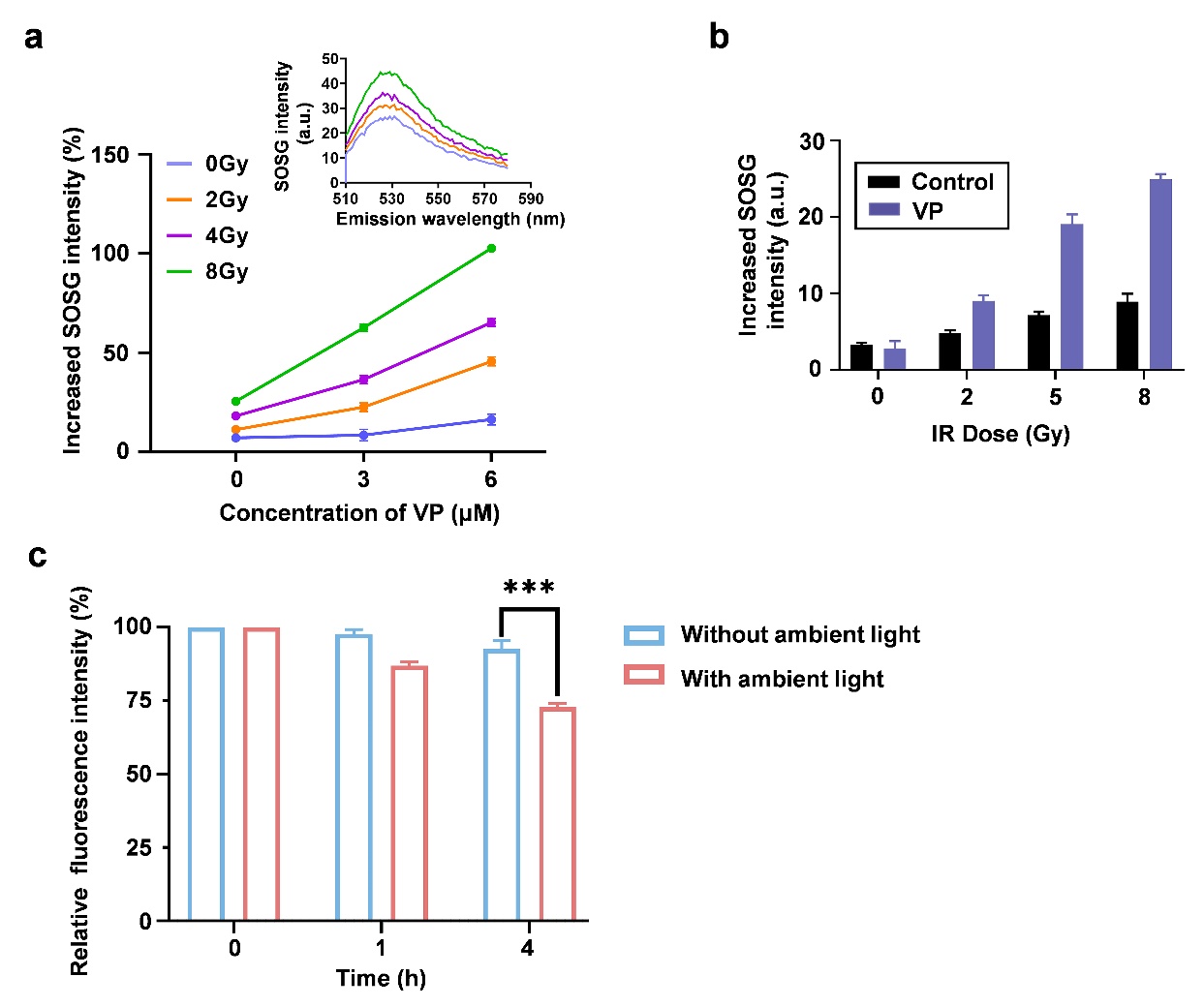


**Figure S1.** ^1^O_2_ generation induced by VP-mediated radiosensitization. a) Increased SOSG intensity of VP (0/3/6 μM) after different dosages of irradiation treatments (n=4). Graph in the upper-right corner: SOSG fluorescence emission spectra (at 488nm excitation) when the samples were subjected to various doses of X-ray irradiation. b) The variation of increased SOSG intensity with X-ray dose in control group and VP group. c) Relative SOSG fluorescence intensity of VP (suspended in PBS) received ambient light excitation (incandescent light) or not for 0, 1 and 4 hours.


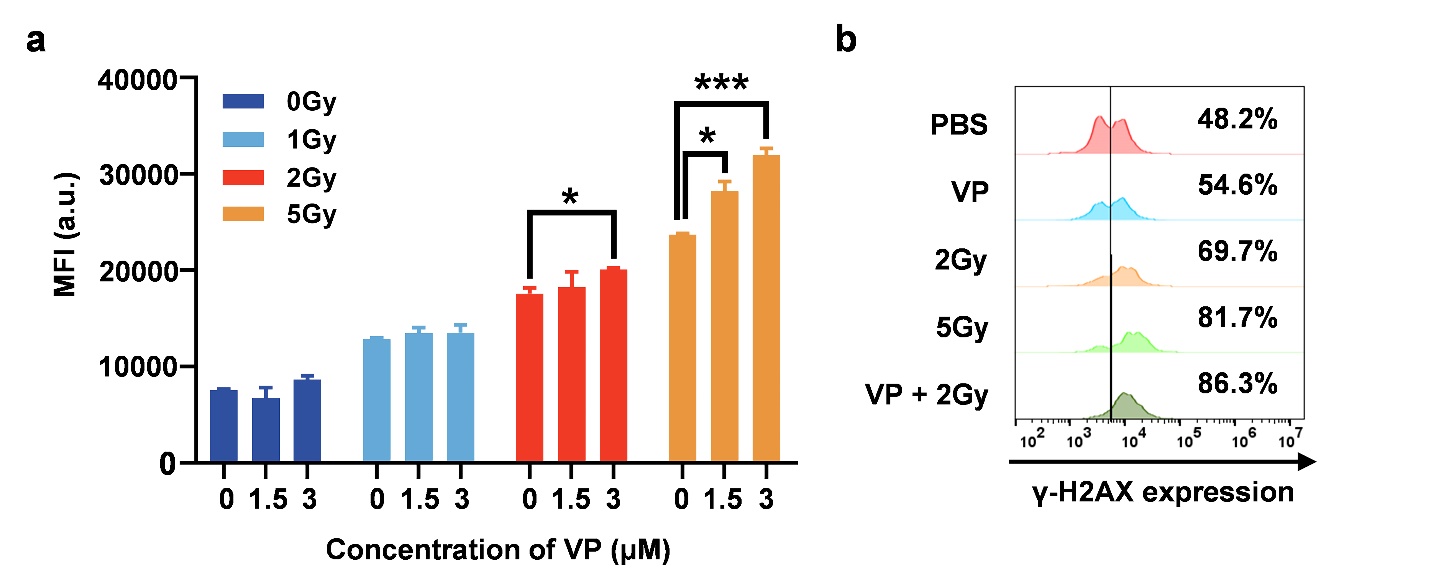


**Figure S2.** DNA damage induced by VP-mediated radiosensitization. a) The mean fluorescence intensity of γ-H2AX in LLC cells treated with VP (0/1.5/3 μM) and IR (0/1/2/5 Gy) was quantified by flow cytometry (n=3). Statistical significance was calculated by Students’ t-test. P-value: * p < 0.05; ** p < 0.01; *** p < 0.001. b) Flow cytometry analysis of γ-H2AX-FITC expression in LLC cells after different treatments.


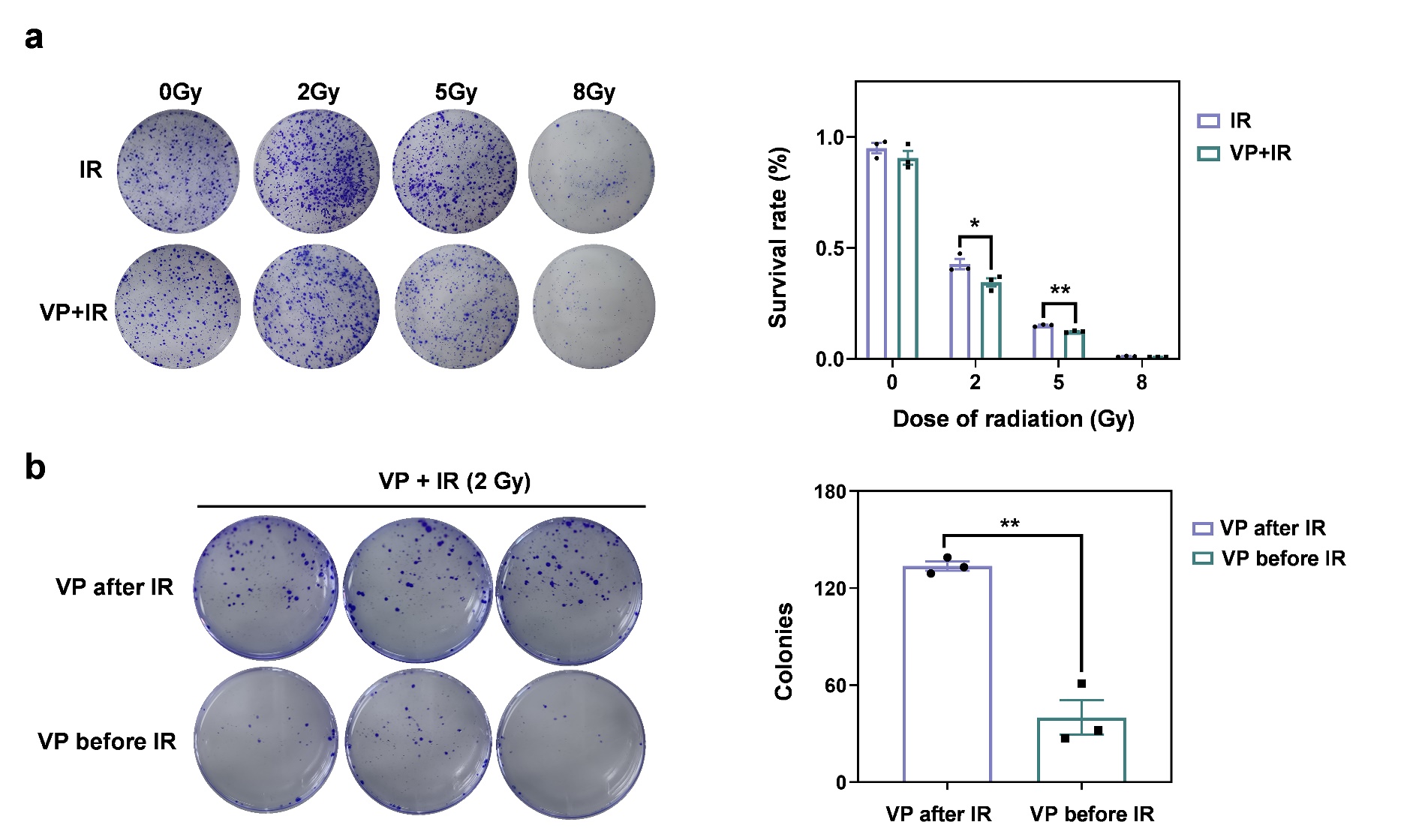


**Figure S3.** Radiosensitization of VP. a) Clonogenic assay. Example of stained colonies at 10^th^ day following IR treatment or VP + IR treatment (left), along with the survival rates of various treatment groups (right). Cells were initially seeded at densities of 500 cells (0 Gy), 2,000 cells (2 Gy), 5000 cells (5 Gy) and 20,000 cells (8 Gy) in each 6-cm dish. b) Comparison of surviving LLC colonies between VP treatment before IR and VP treatment after IR. 1,000 LLC cells were initially inoculated per well in 6-well plates. P value was calculated by Students's t-test, p-value: * p < 0.05; ** p < 0.01.


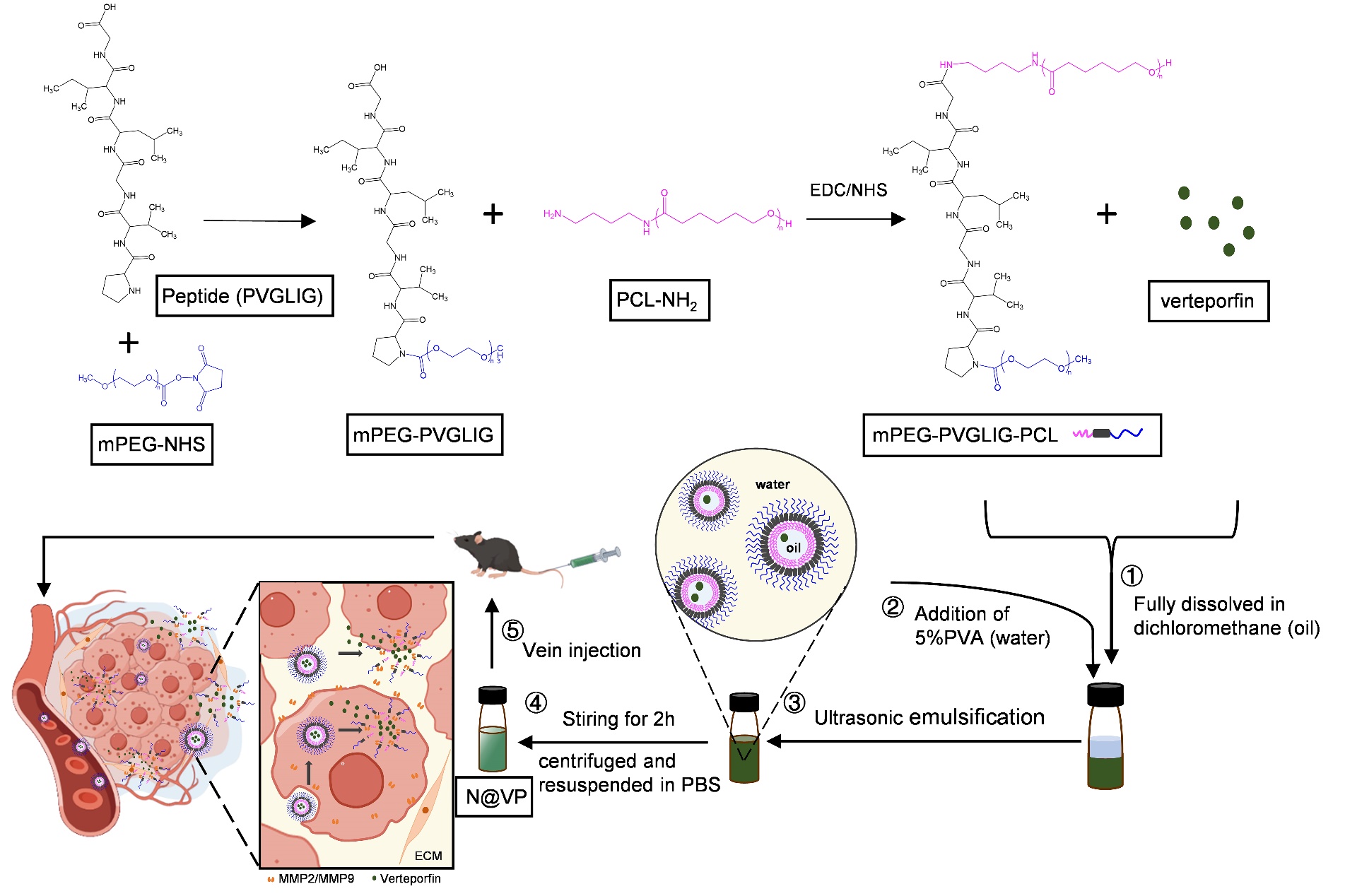


**Figure S4.** Schematic representation for N@VP actively targeting tumors in vivo, and synthesis process of the nanocarriers (mPEG-PVGLIG-PCL) and verteporfin-loaded nanoparticles (N@VP).


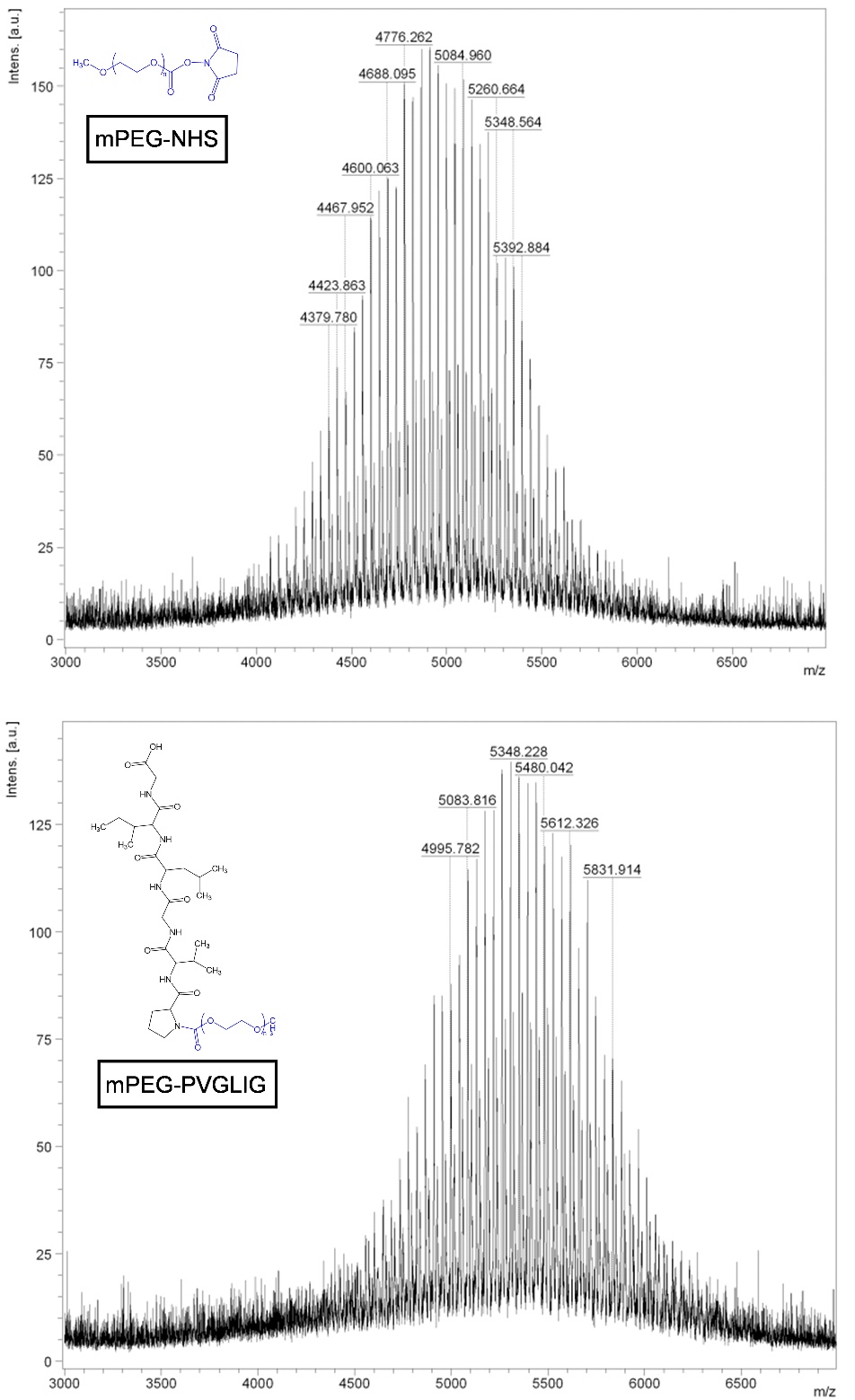


**Figure S5.** MALDI-TOF mass spectrum of mPEG-NHS and mPEG-PVGLIG.

**
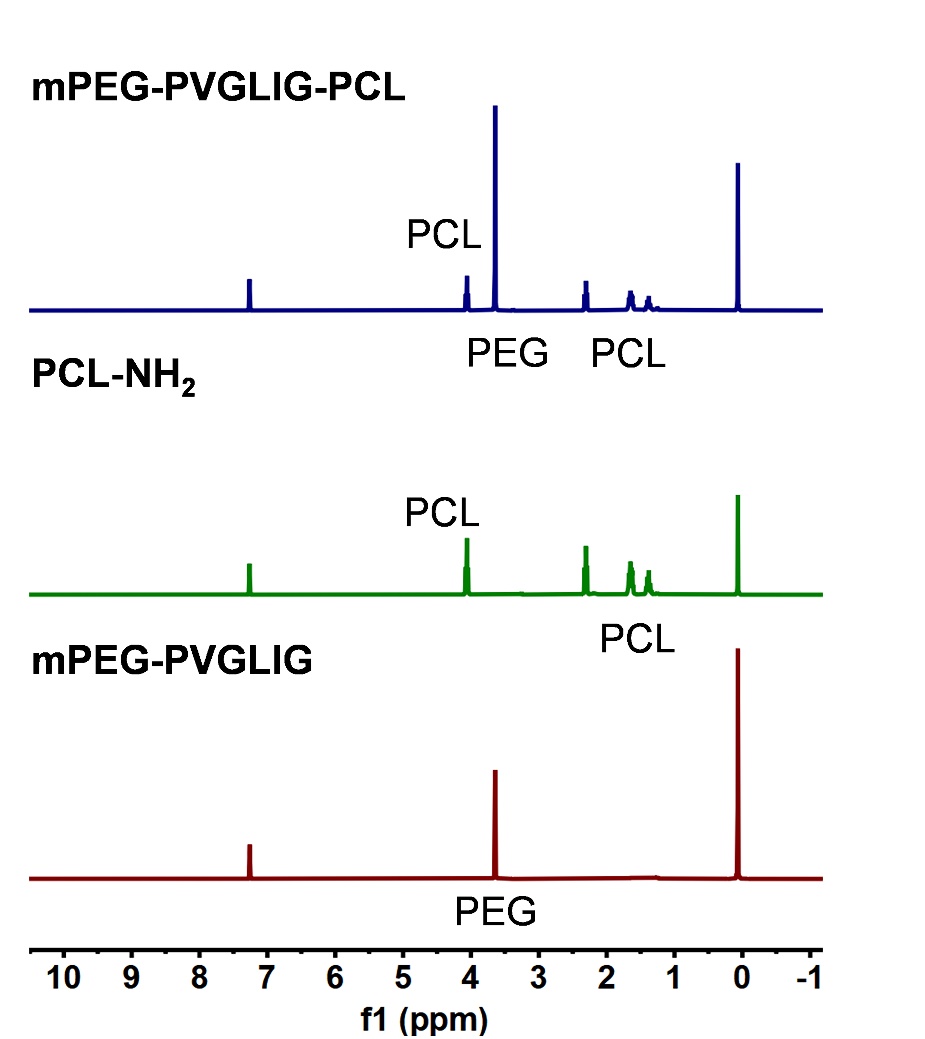
**

**Figure S6.** ^1^H NMR spectrum of mPEG-PVGLIG, PCL-NH_2_ and mPEG-PVGLIG-PCL in CDCl_3_.

**
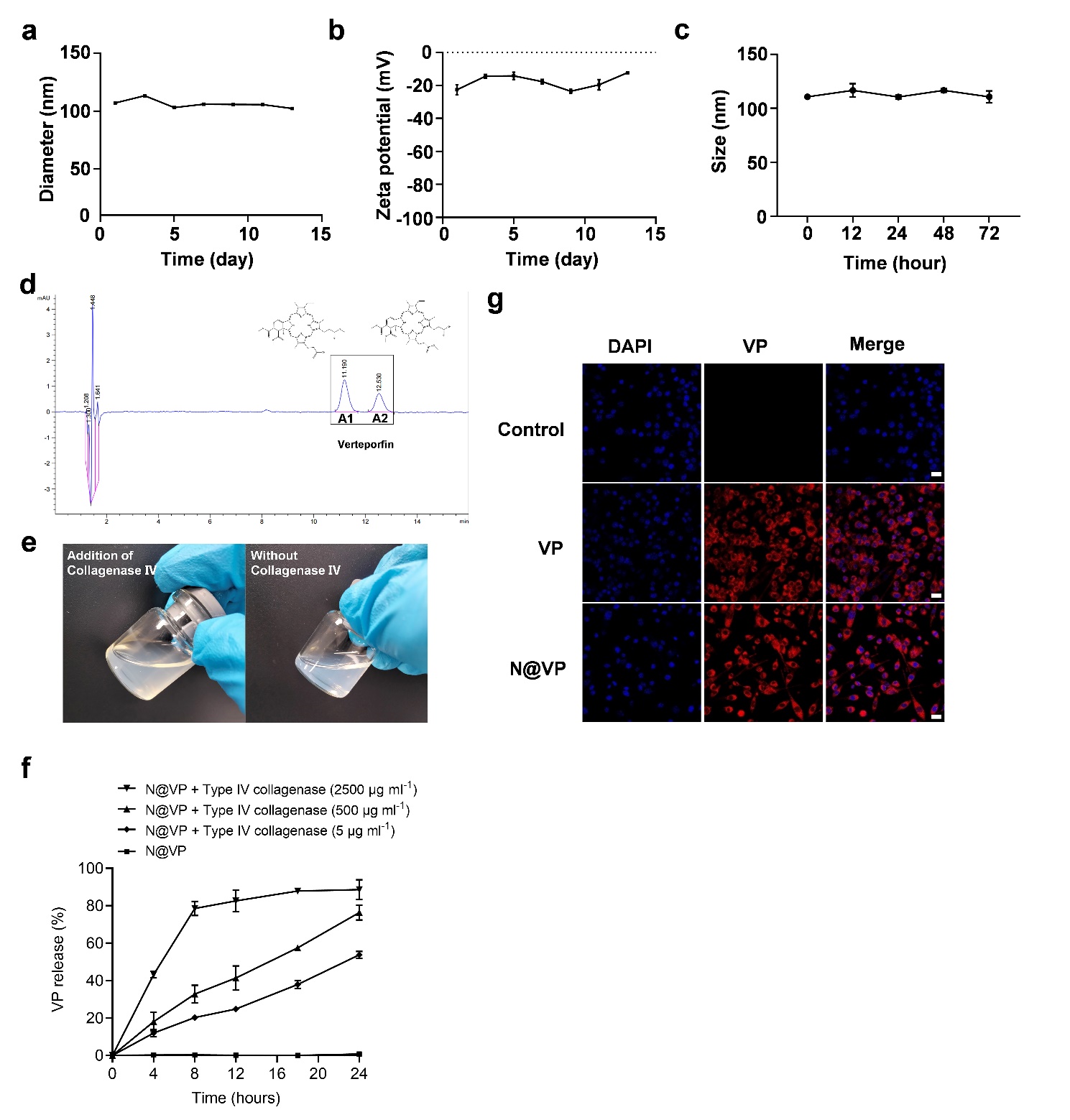
**

**Figure S7.** Characterization of N@VP. Changes of N@VP a) particle size and b) zeta potential with time. c) The stability studies in particle size changes of the N@VP in 100% FBS. The size distribution of the nanoparticles was measured at different time points (0, 12, 24, 48, 72 h) by DLS instrument during incubation with FBS at 37℃. d) HPLC chromatogram of VP. A1 and A2 are regioisomers of VP. e) Blank mPEG-PVGLIG-PCL nanoparticles were incubated with or without Collagenase Ⅳ in PBS at 37 ℃ for 24 hours. f) Cumulative release rate of VP from N@VP after treatment with different concentrations of gelatinase. g) Cellular uptake of VP and N@VP were visualized using confocal laser scanning microscopy (blue: DAPI; Red: VP). Scale bar was 20 μm.

**
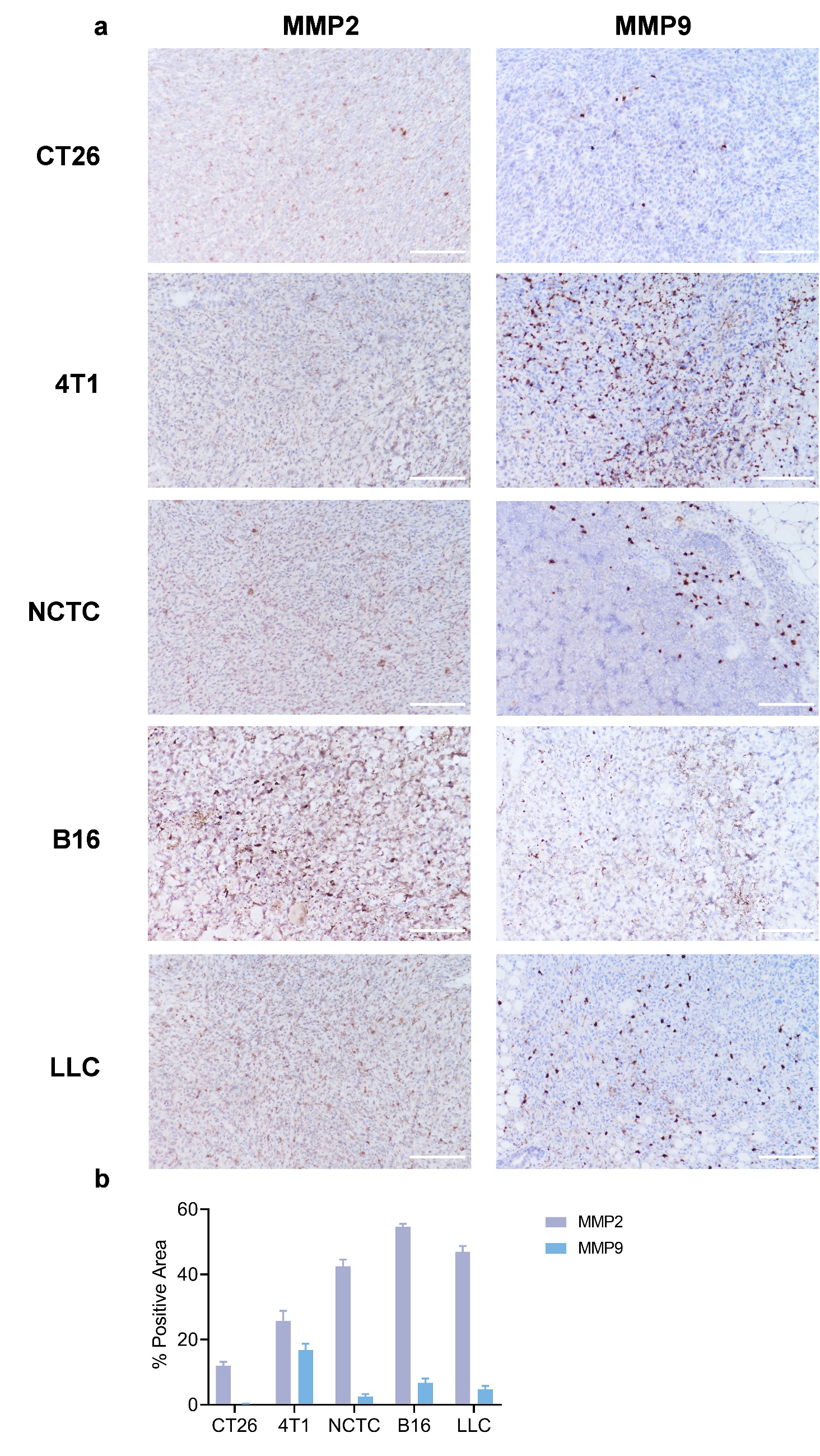
**

**Figure S8.** Expression of MMP2 and MMP9 in various tumor tissues. a) Immunohistochemical staining of the gelatinase (MMP2/9) expression in CT26 cells, 4T1 cells, NCTC cells, B16 cells and LLC cells. Scale bar, 200 µm. b) The percentages of MMP2 and MMP9 positive-stained area measured by ImageJ software.


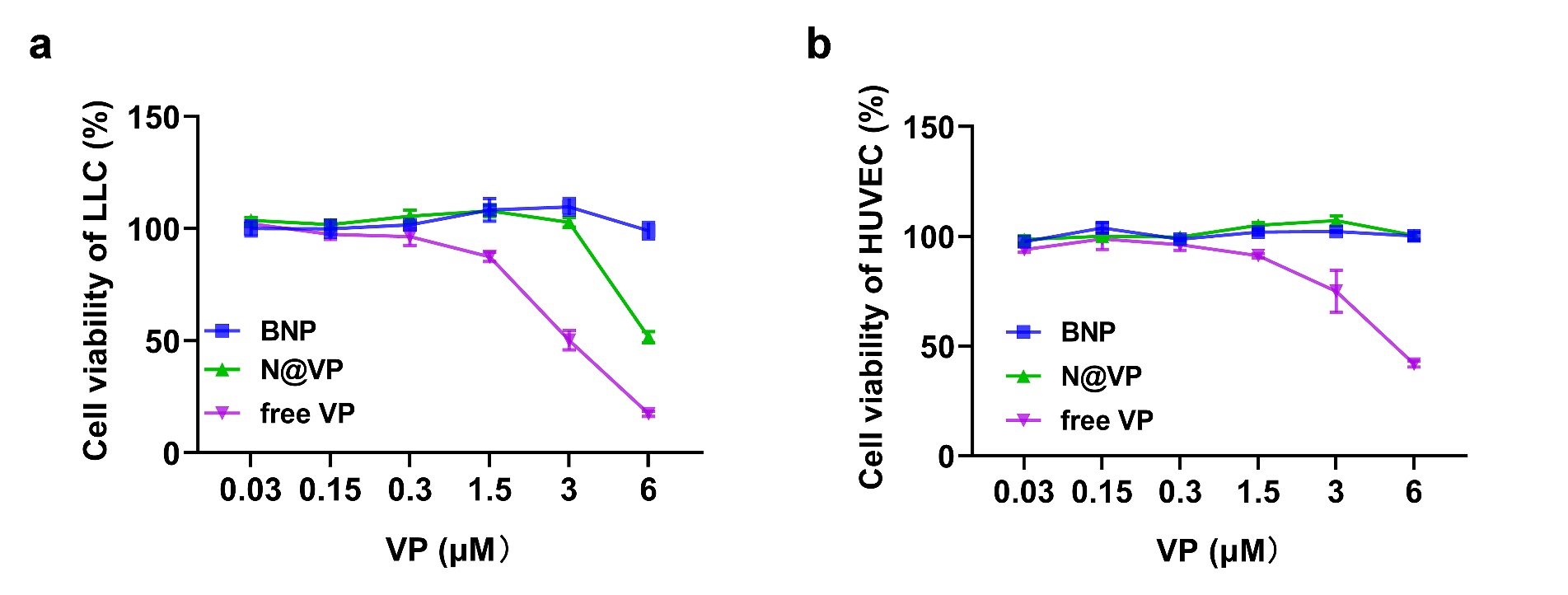


**Figure S9.** In vitro cytotoxicity assay. Viability of a) LLC cells and b) HUVEC cells treated with various concentrations of VP-loaded nanoparticles (N@VP), free VP, and blank nanoparticles (BNP) for 24 h. P value was evaluated by one-way ANOVA (*p < 0.05; **p < 0.01; ***p < 0.001; ****p < 0.0001).

**
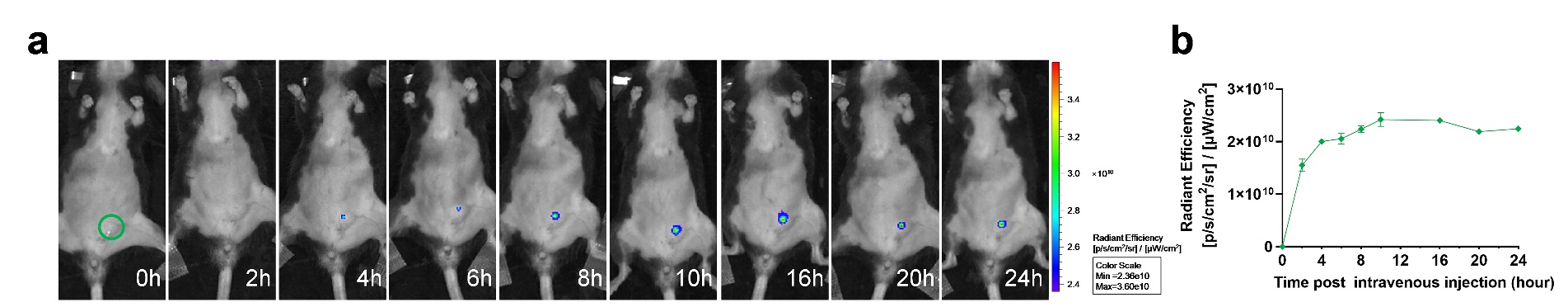
**

**Figure S10.** Biodistribution and tumor targeting of N@DiR in vivo. a) The in vivo NIR imaging and b) the quantitative curve (n=3) of DiR signals within tumor tissues over time after systemically delivering N@DiR. Tumor was circled with green circle at 0 h.


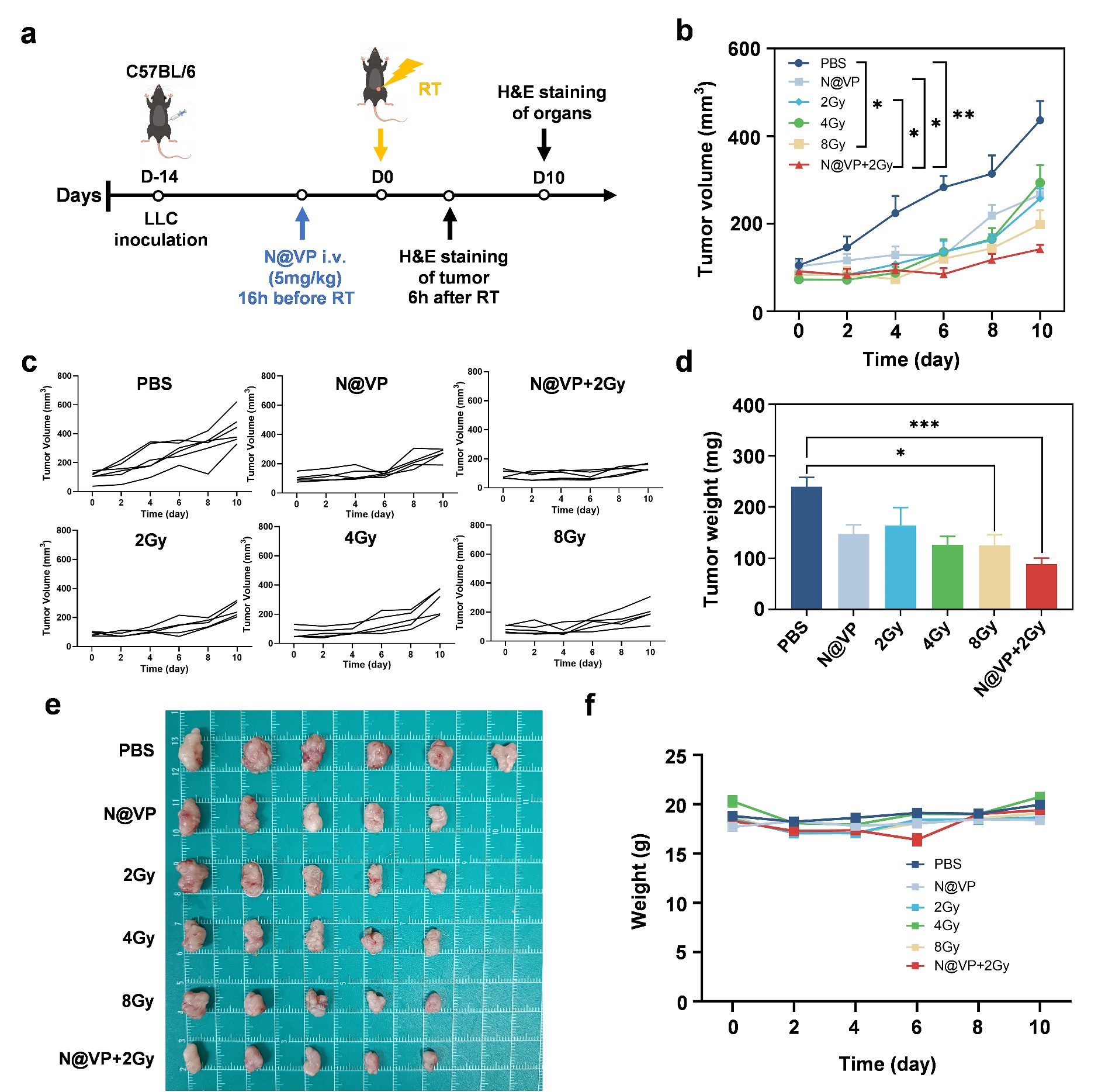


**Figure S11.** In vivo tumour inhibitory effect of N@VP + 2 Gy. a) Illustration of the experimental procedure. b, c) Tumor growth on mice bearing Lewis lung cancer xenograft with different treatments (n=5-6). P value was calculated by two-way ANOVA. P-value: * p < 0.05; ** p < 0.01. d) Weight and e) photographs of tumors isolated at the endpoint. P value was calculated by one-way ANOVA. P-value: * p < 0.05; *** p < 0.001. f) Changes of mice body weight during treatment in each group.

**
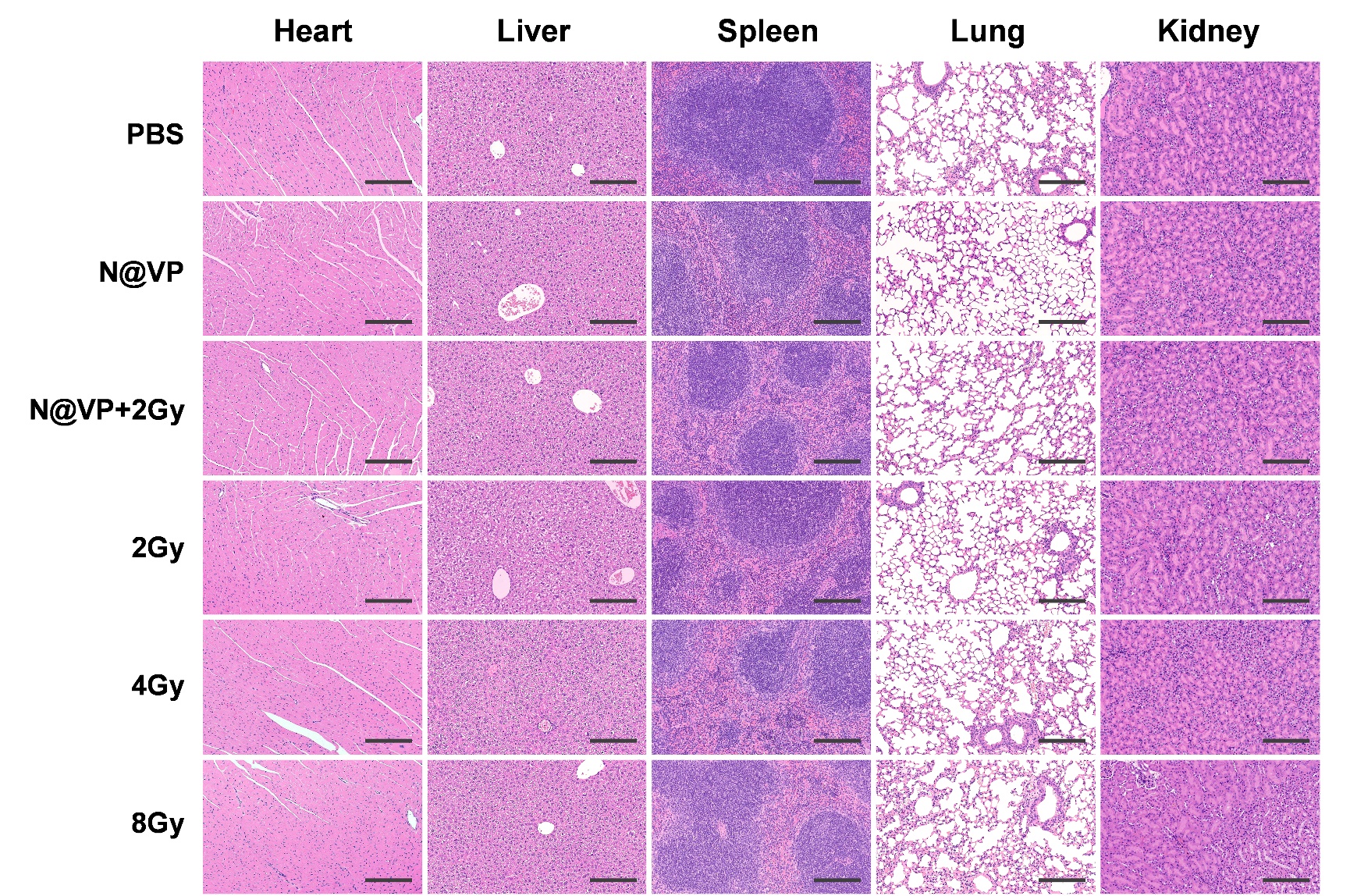
**

**Figure S12.** Histopathology analyses of organ sections from Lewis lung cancer xenografted mice after the tumor growth inhibition experiment. The scale bar is 200 μm.

**
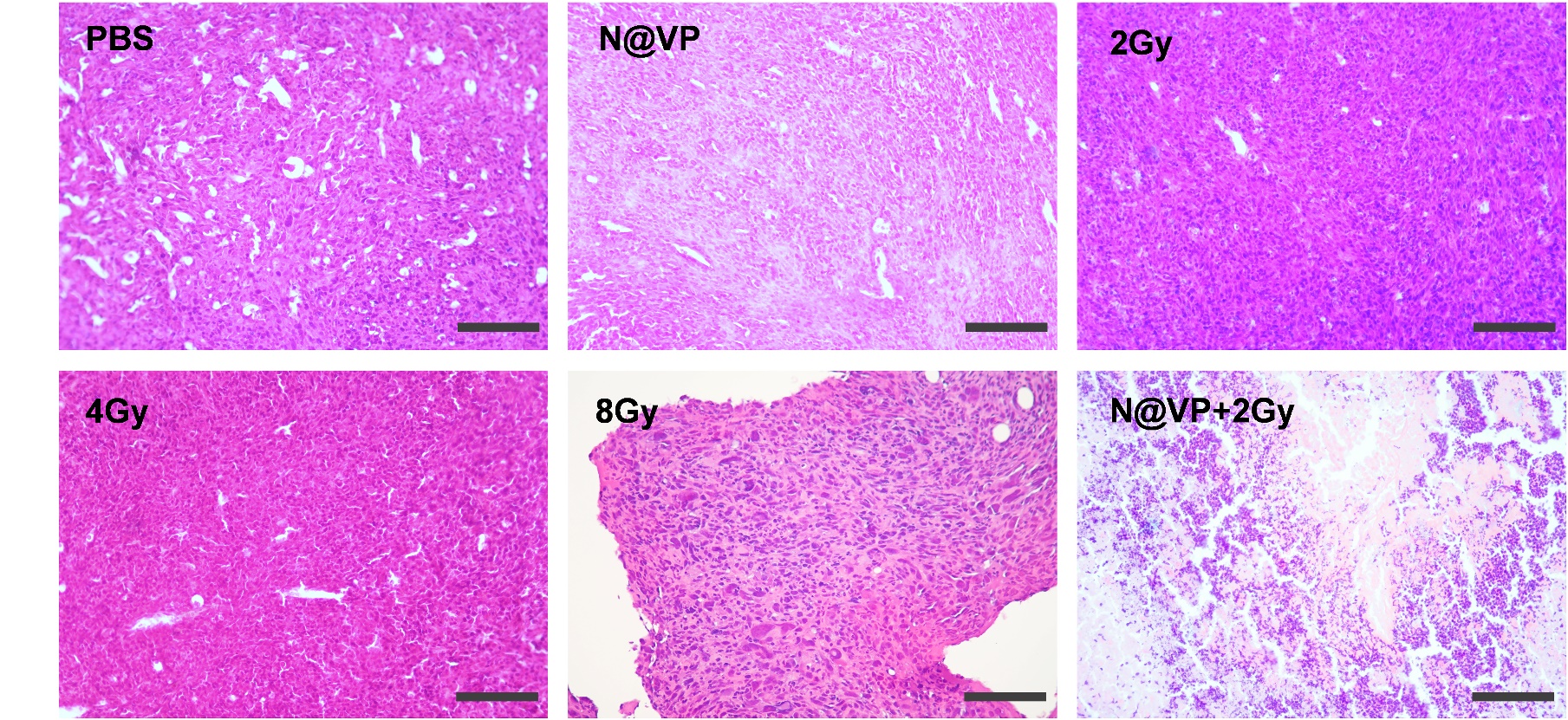
**

**Figure S13.** Histopathology analyses of tumor sections from Lewis lung cancer xenografted mice after the tumor growth inhibition experiment. The scale bar is 100 μm.

**
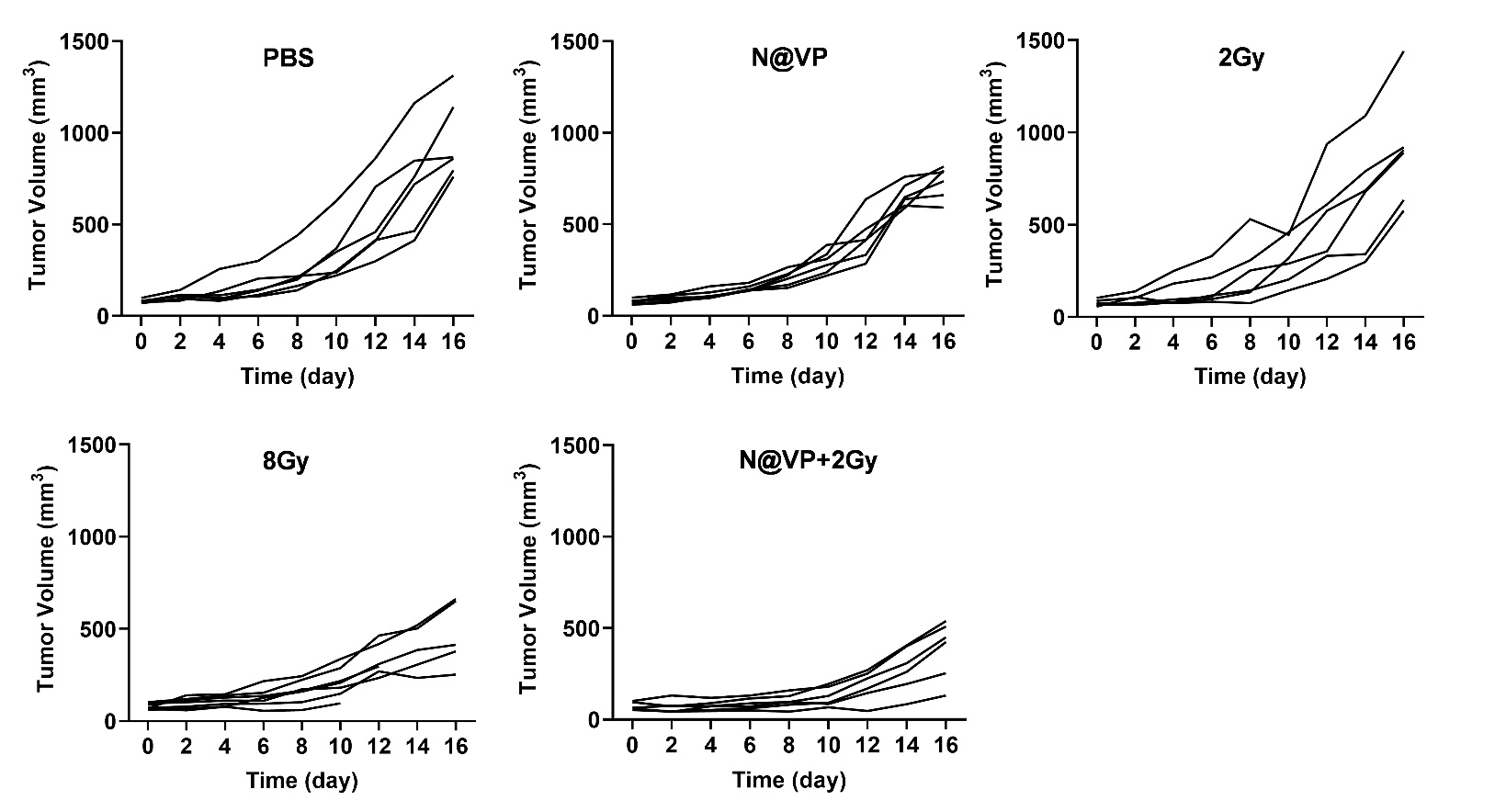
**

**Figure S14.** Tumor growth of C57BL/6 mice bearing Lewis lung cancer xenograft with different treatments (n=6).

**
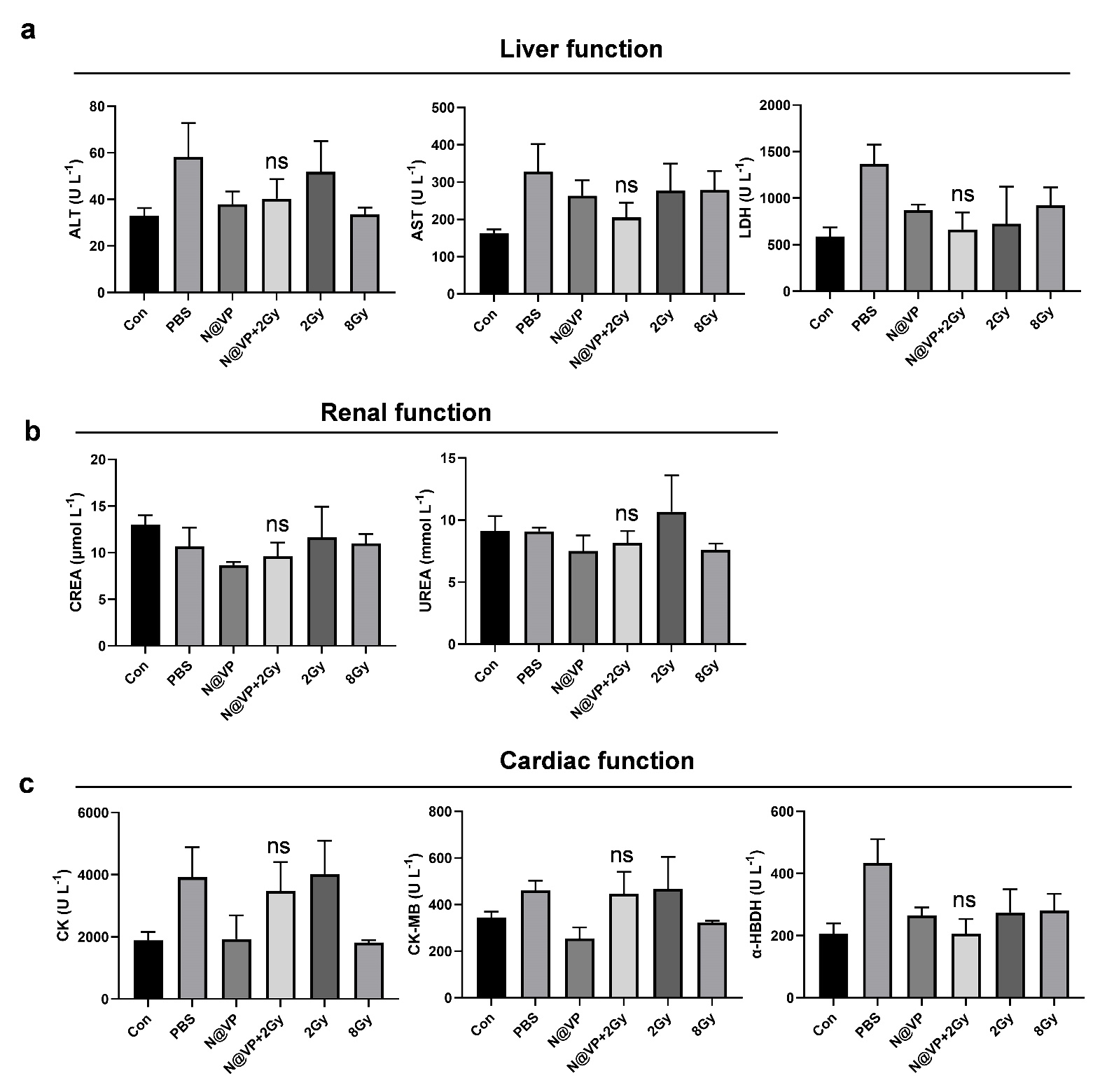
**

**Figure S15.** Safety study of N@VP + 2 Gy treatment. Blood samples were collected from the eyeball for biochemical examination on the third day after the last treatment. Three no-tumor C57BL/6 mice were used as normal control group (con). The measured biochemical markers include a) liver function (ALT, AST, LDH), b) renal function (CREA, UREA), and c) cardiac function (CK, CK-MB, α-HBDH). P values above the bar graphs are reported as a measure of statistical significance compared with the control group and were calculated by one-way ANOVA (ns represents p > 0.05).


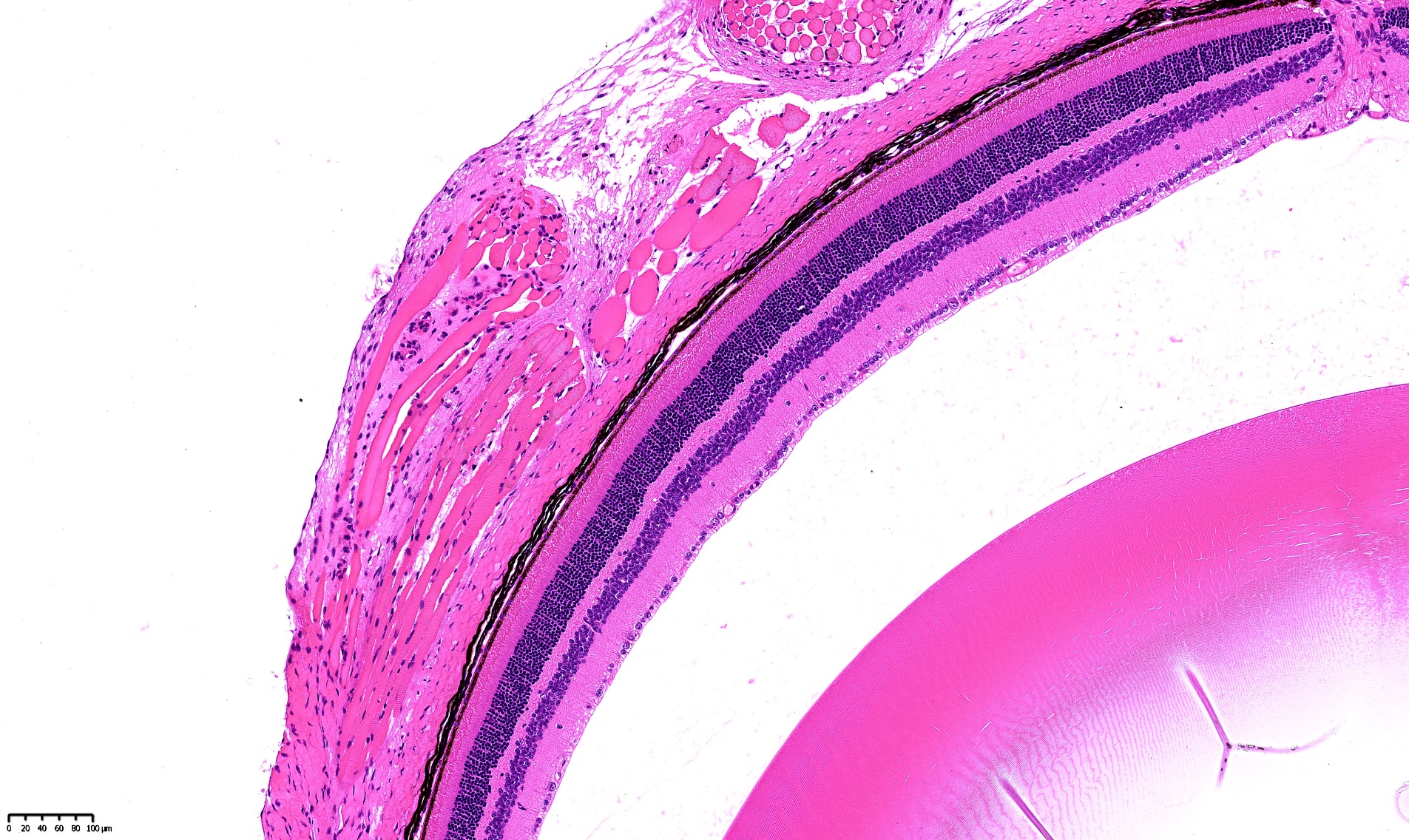


**Figure S16.** LLC-bearing mice were treated with three cycles of N@VP + 2 Gy treatment. H&E staining of eyes from mice at the end of the fourth week following the last treatment. Scale bar: 100 μM. The layers of the retina are clearly demarcated, with normal cellular morphology and orderly arrangement. The choroidal capillaries and pigment cells are abundant. The sclera demonstrates elastic fibers and collagen fibers interwoven into a regular and densely arranged network. The corneal epithelium presents as stratified squamous epithelium with normal cellular morphology and regular arrangement. The stromal connective tissue shows collagen fibers and elastic fibers interwoven into an organized network. No significant pathological changes are observed, injury score = 0.


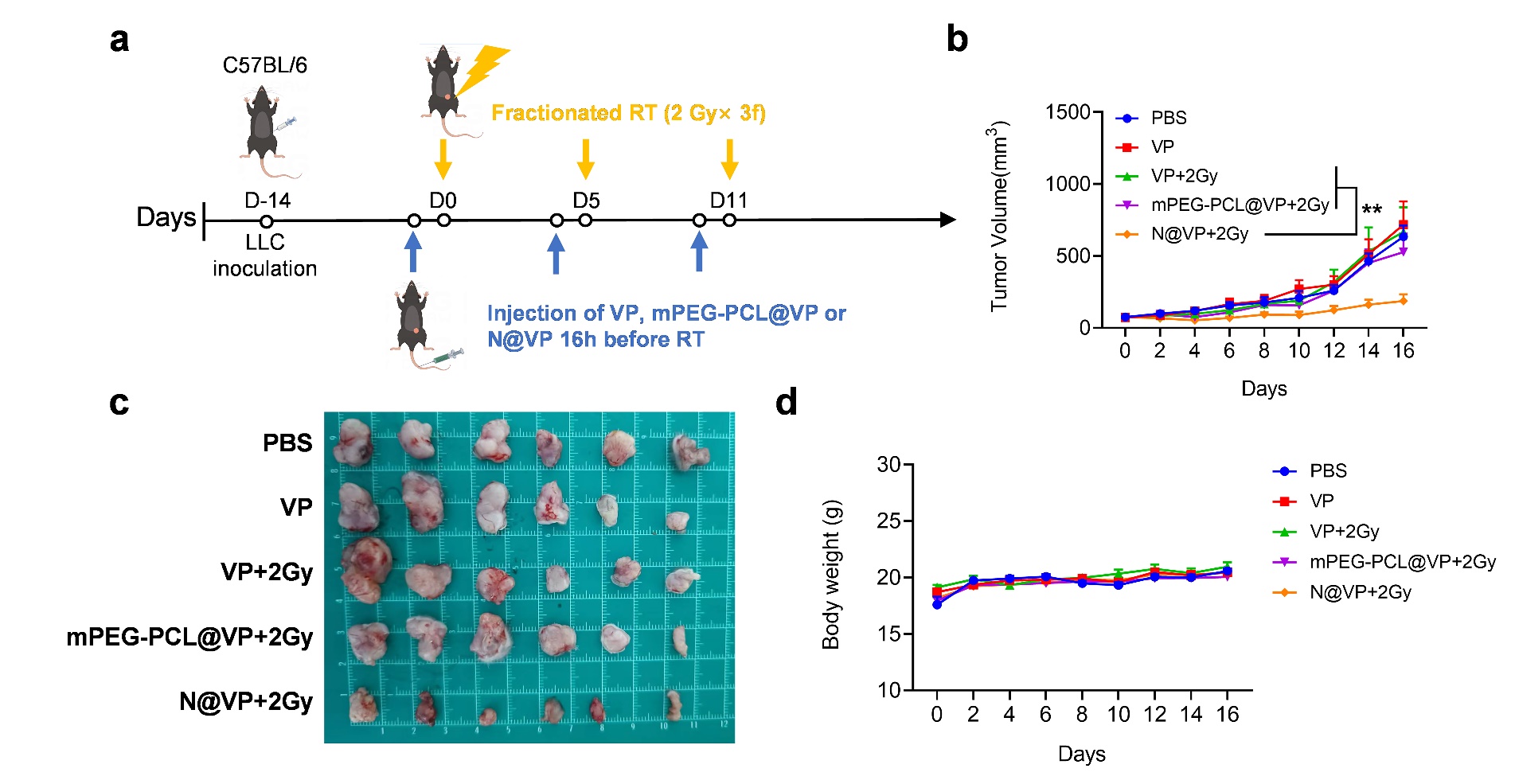


**Figure S17.** In vivo radiosensitizing effects of N@VP in contrast to VP and mPEG-PCL@VP. a) Schematic diagram of the experimental design. b) Tumor growth of mice bearing Lewis lung carcinoma in different groups (n=6). c) Photographs of tumors isolated at the end of experiment. d) Body weight curves (n=6).

**
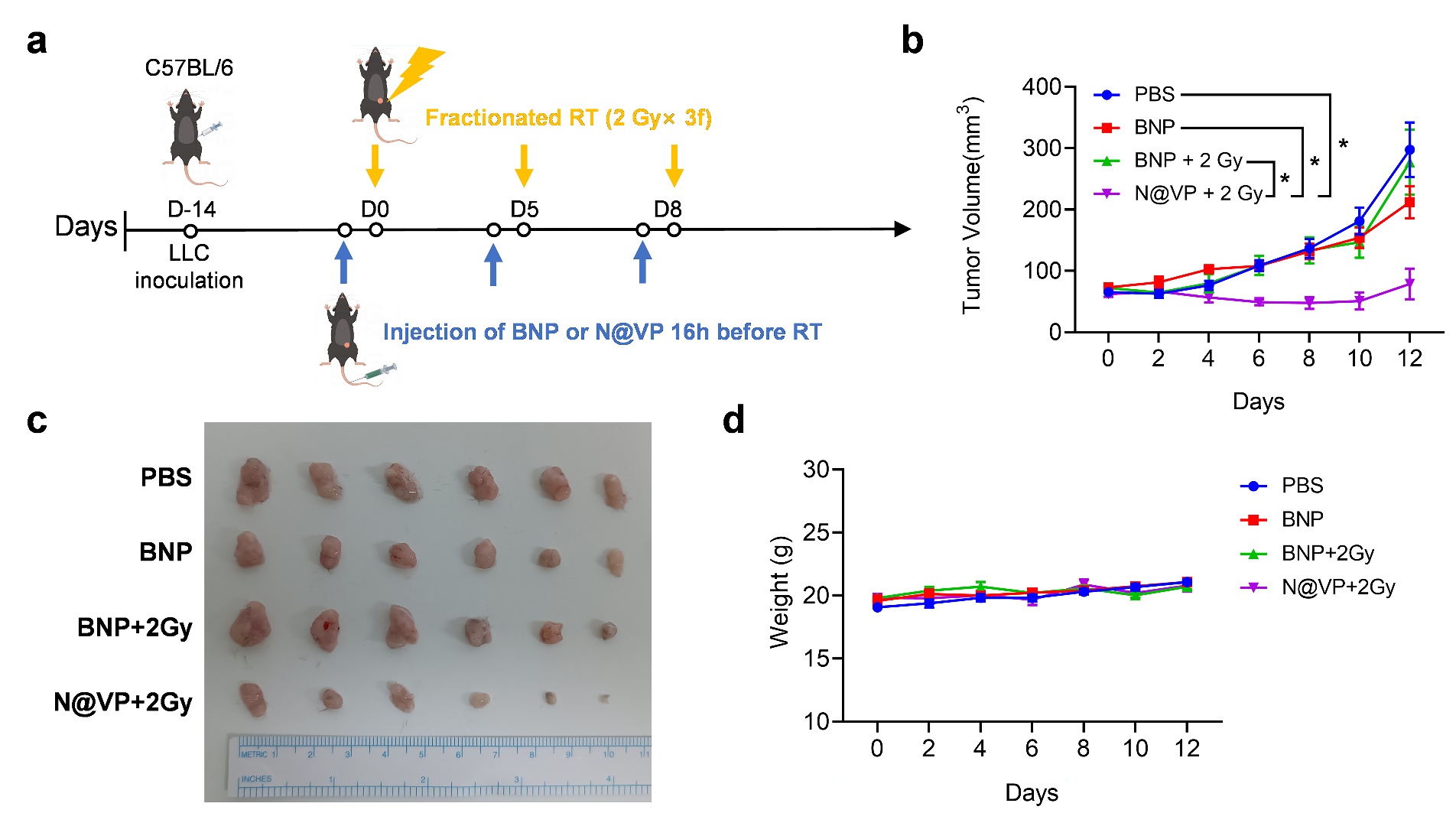
**

**Figure S18.** Antitumor effect of BNP and BNP + 2 Gy. a) Schematic diagram of the experimental design. b) Tumor growth of mice bearing Lewis lung carcinoma in different groups (n=6). P value was calculated by two-way ANOVA. P-value: * p < 0.05; ** p < 0.01; *** p < 0.001. c) Photographs of tumors isolated on the 12^th^ day. d) Body weight curves (n=6).

**
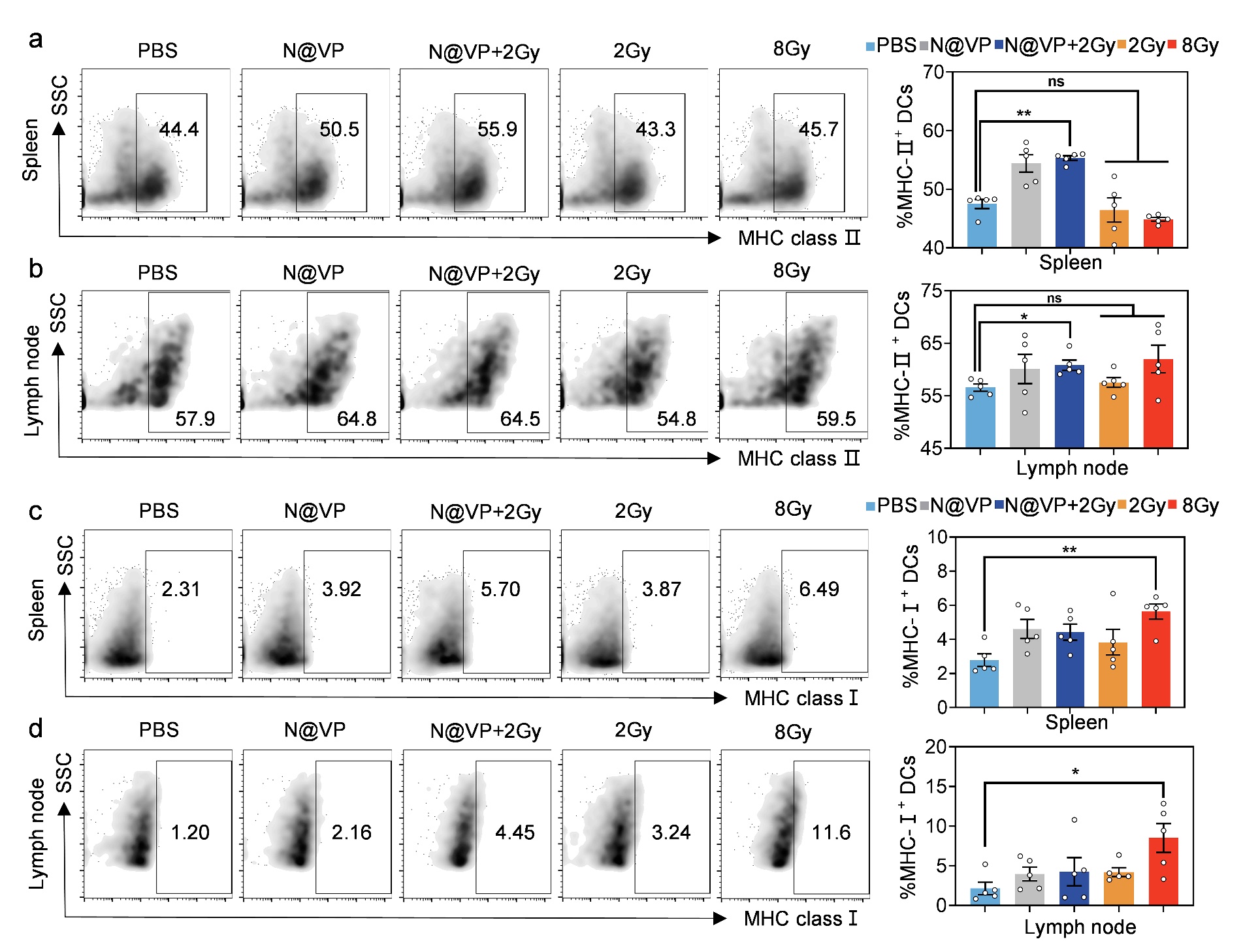
**

**Figure S19.** MHC class Ⅰ and Ⅱ expression. a) Representative flow cytometric plots and statistical graphs of MHC class Ⅱ expression on CD11C^+^ DCs in spleens from LLC-bearing mice after various treatment. P value was calculated by one-way ANOVA (*p < 0.05; **p < 0.01; ***p < 0.001; ****p < 0.0001, ns represents p > 0.05). b) MHC class Ⅱ expression on CD11C^+^ DCs in lymph nodes from LLC-bearing mice after various treatment. Data were analyzed by Students’ t-test (*p < 0.05; **p < 0.01; ***p < 0.001; ****p < 0.0001, ns represents p > 0.05). c, d) MHC class Ⅰ expression on CD11C^+^ DCs in spleens and lymph nodes from mice bearing Lewis Lung Carcinoma that experienced different treatments. The proportion was presented on the right. Data was evaluated by one-way ANOVA (*p < 0.05; **p < 0.01; ***p < 0.001; ****p < 0.0001).

**
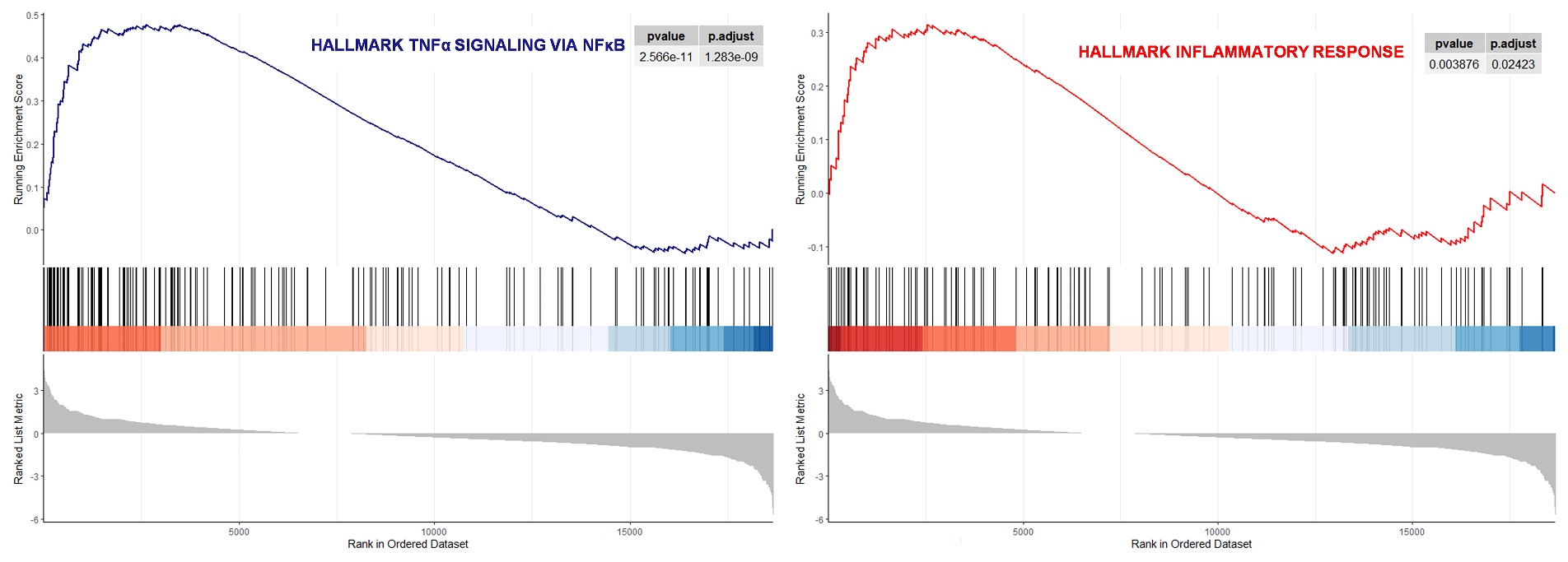
**

**Figure S20.** Significantly activated pathways (HALLMARK INFLAMMATORY RESPONSE and HALLMARK TNFα SIGALING VIA NFκB) enriched by DEGs in the two groups’ RNA sequencing data (Con vs VP+IR) using GSEA, p.adjust < 0.05. GSEA used the curated hallmark gene set collection from MSigDB.

**
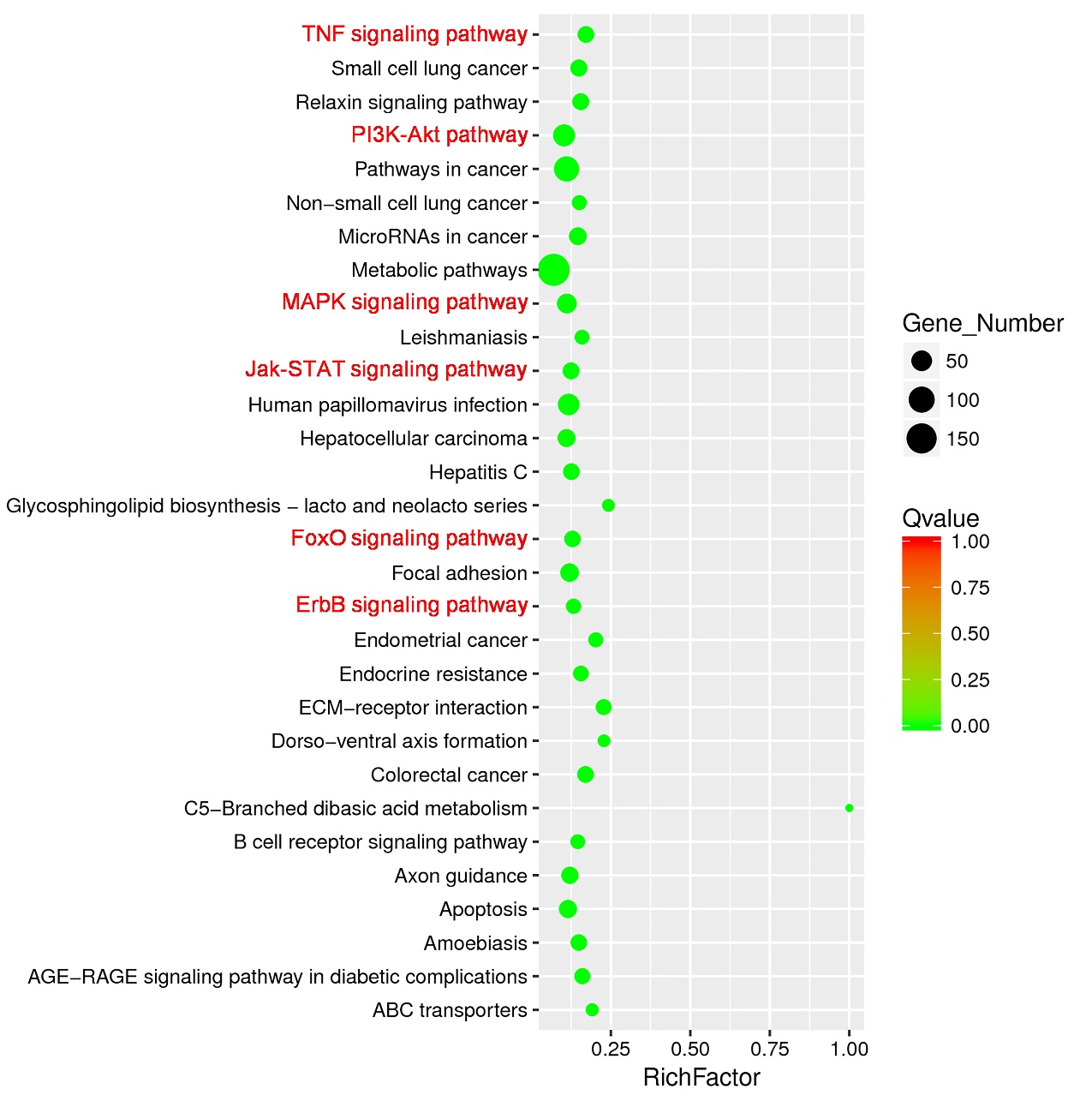
**

**Figure S21.** KEGG enrichment analysis focused on comparing the gene expression differences between the Con group and the VP + IR group.


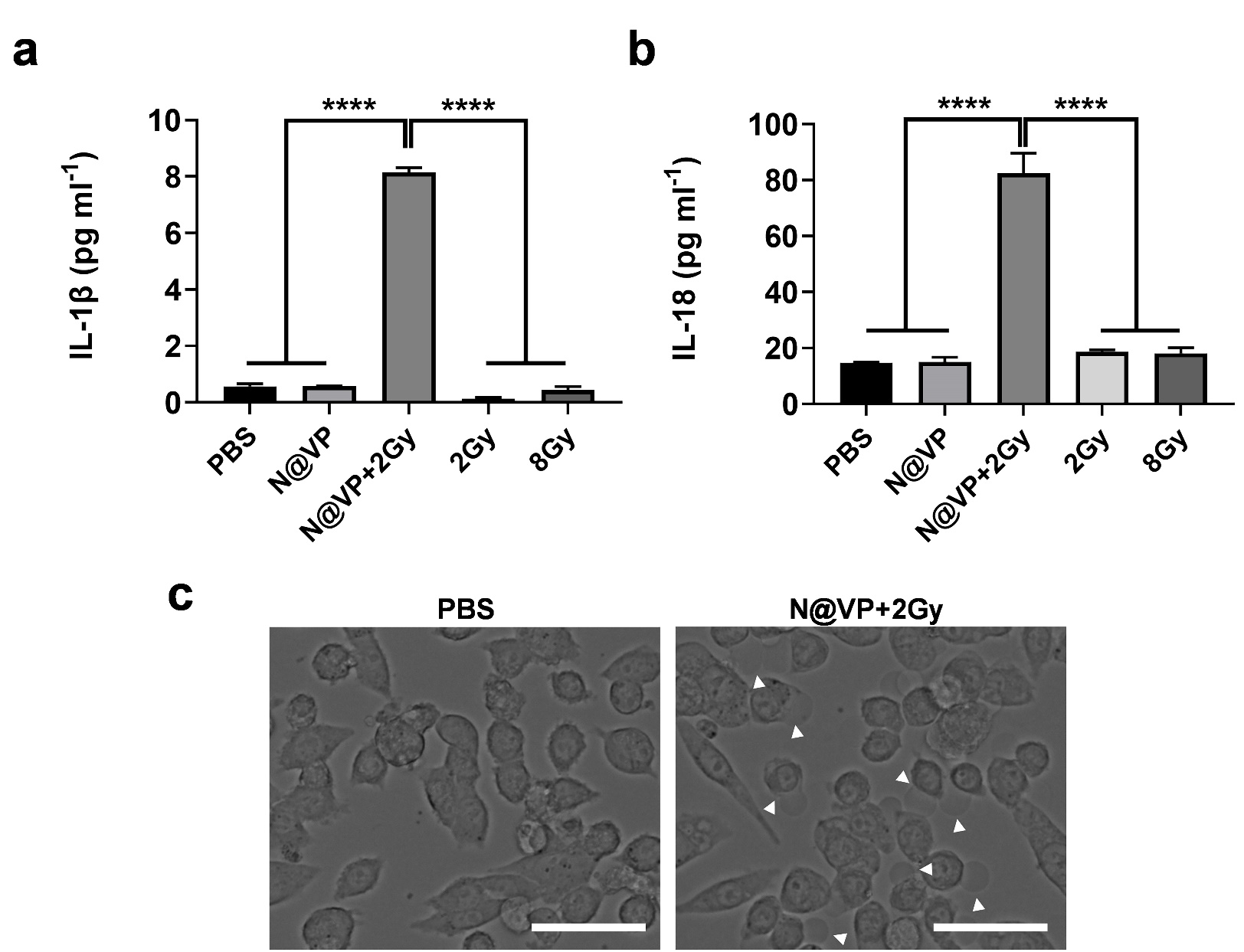


**Figure S22.** N@VP combined with IR induced tumor cell pyroptosis in vitro. a) IL-1β and b) IL-18 secretion by LLC cells into the supernatant was measured by ELISA 12 h after various treatment. P values was calculated by one-way ANOVA. (****p<0.0001). c) 6 h after treatment with N@VP-mediated radiosensitization, LLC cells exhibited typical pyroptosis morphology with cell swelling and membrane blebbing. White arrows indicate pyroptotic "ballooning" structures. Scale bar: 50 µm.

**
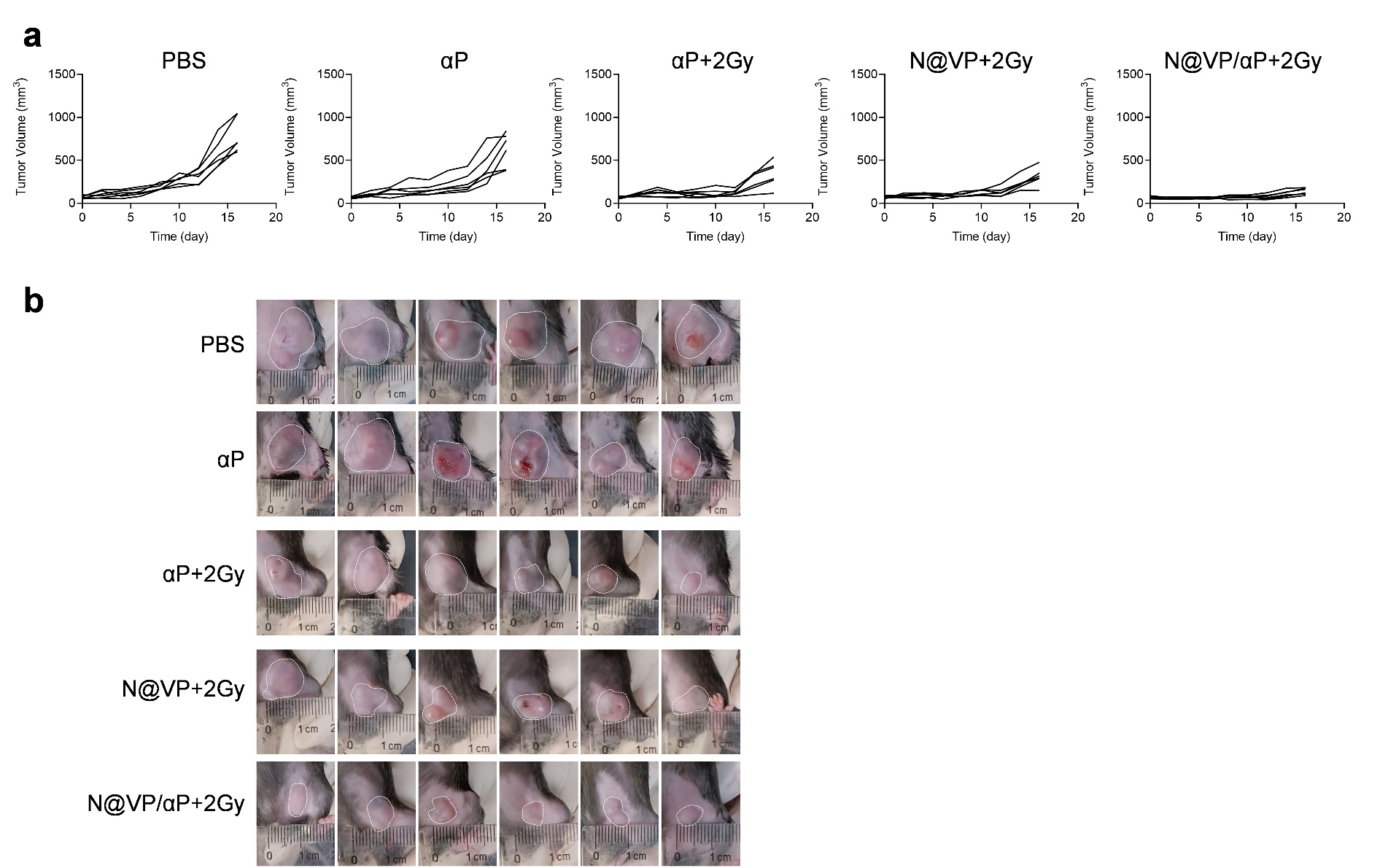
**

**Figure S23.** N@VP + 2 Gy synergized with checkpoint blockade immunotherapy to inhibit tumor growth. a) Tumor curves of each group. b) Photographs of tumors at end point of the experiment.

**
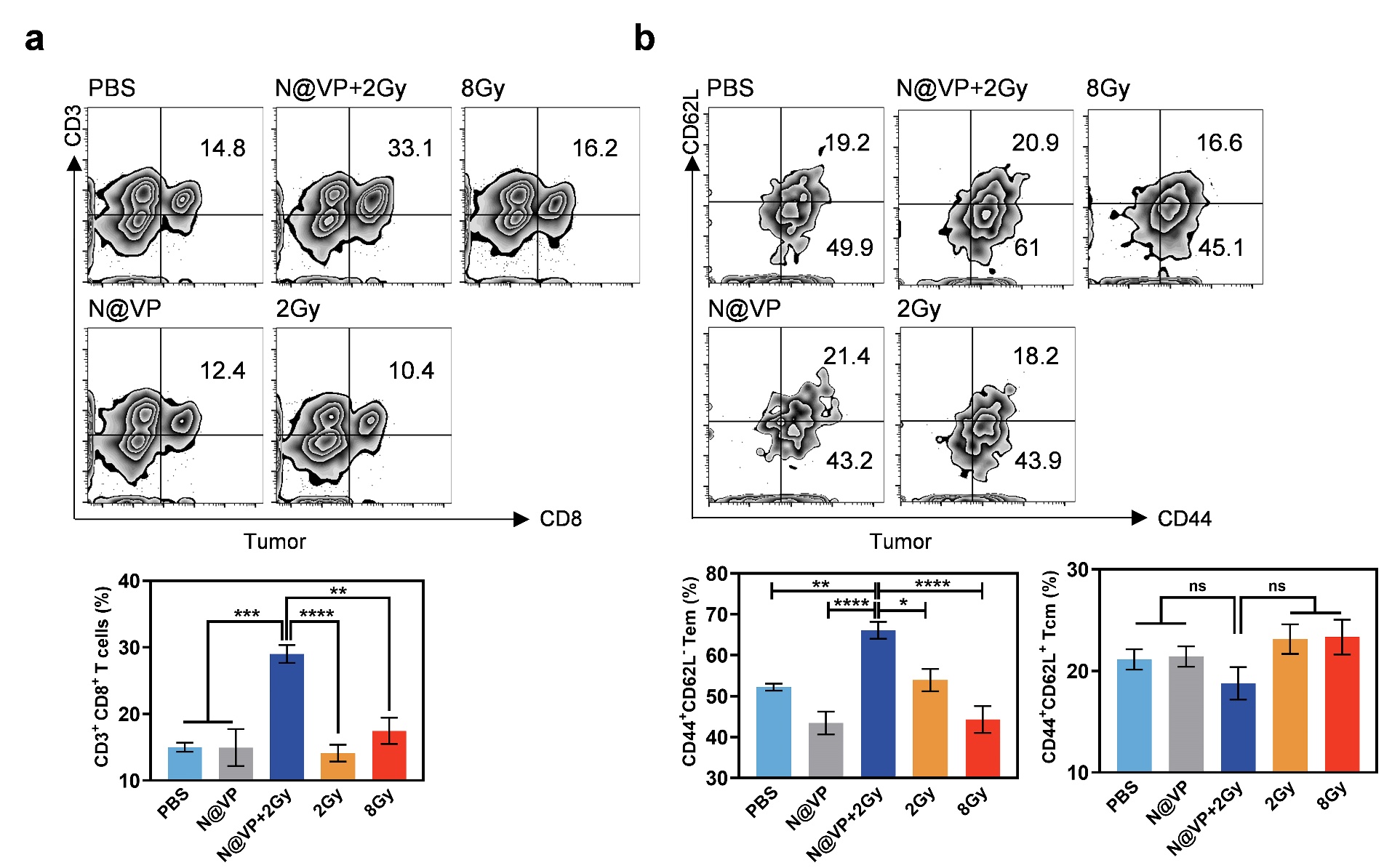
**

**Figure S24.** Enhanced memory CD8^+^ T cells infiltration in tumor microenvironment induced by N@VP-mediated radiosensitization. The investigation was performed on the 14^th^ days after the last treatment. Representative flow cytometry profiles with corresponding quantification (n=5) of a) CD3^+^CD8^+^ T cells and b) CD44^+^CD62L^-^ effector memory (Tem) CD8^+^ T cells (gated on CD3^+^CD8^+^) and CD44^+^CD62L^+^ central memory (Tcm) CD8^+^ T cells percentages (gated on CD3^+^CD8^+^) in tumor tissues from LLC-bearing C57BL/6 mice undergoing various treatments. P value was evaluated by one-way ANOVA (*p < 0.05; **p < 0.01; ***p < 0.001; ****p < 0.0001)


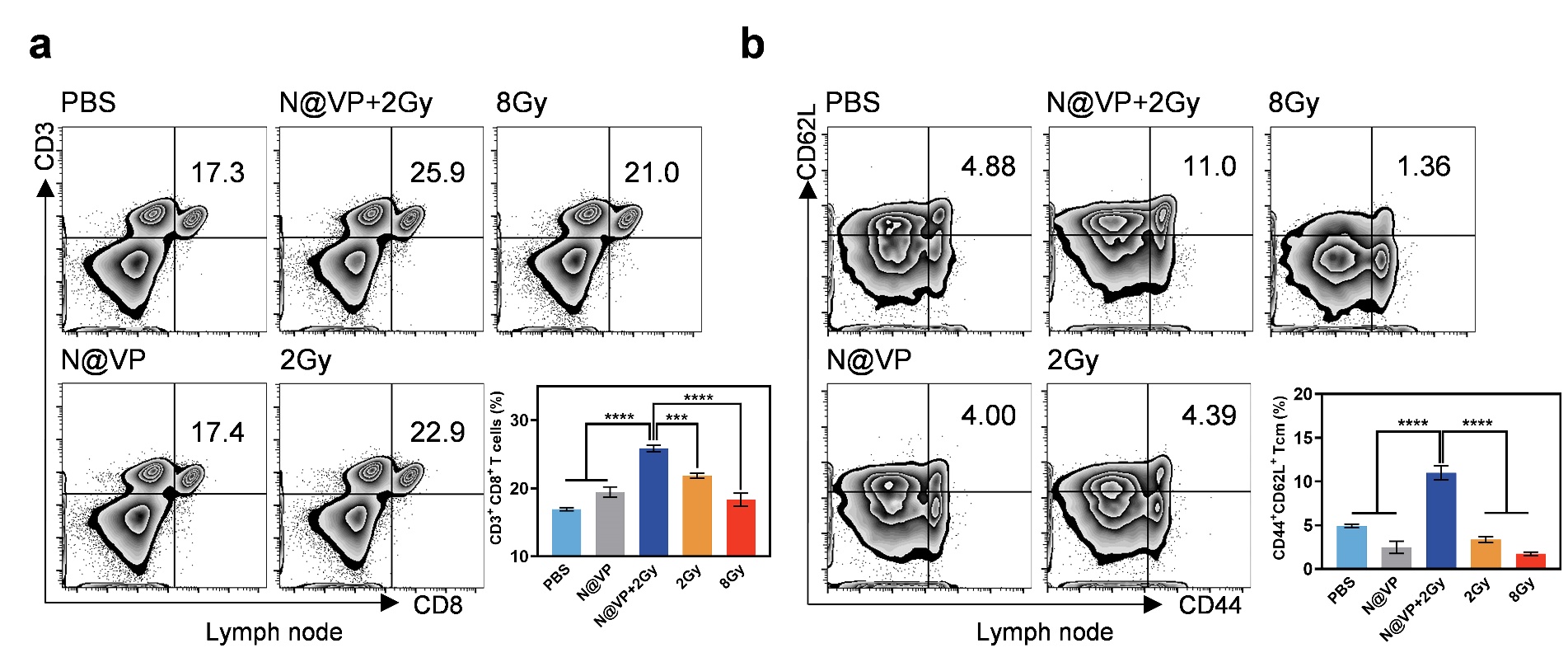


**Figure S25.** Enhanced memory CD8^+^ T cells infiltration in lymph nodes induced by N@VP-mediated radiosensitization. The investigation was performed on the 14^th^ days after the last treatment. Representative flow cytometry profiles with corresponding quantification (n=5) of a) CD3^+^CD8^+^ T cells and b) CD44^+^CD62L^+^ central memory CD8^+^ T cells percentages (gated on CD3^+^CD8^+^) in lymph nodes from LLC-bearing C57BL/6 mice undergoing various treatments. P value was evaluated by one-way ANOVA (*p < 0.05; **p < 0.01; ***p < 0.001; ****p < 0.0001)


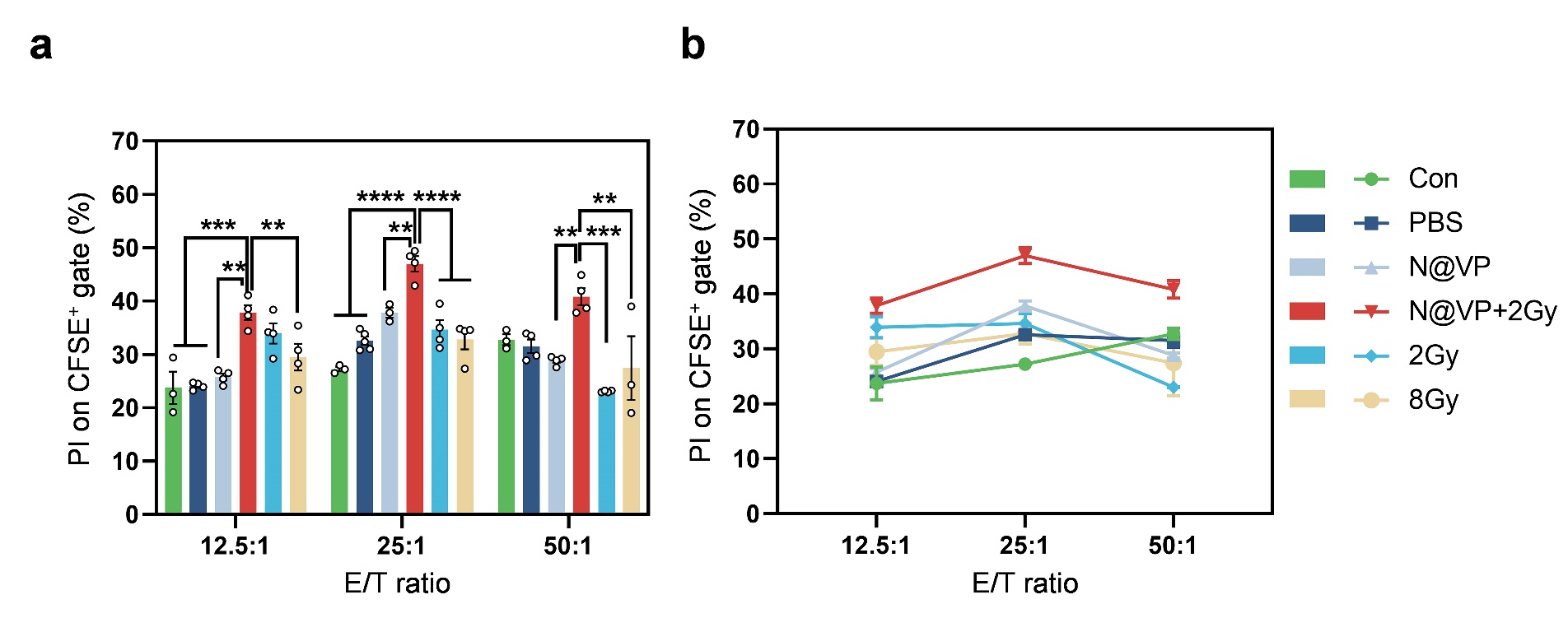


**Figure S26.** T cells collected from mice spleens in different groups preserve cell-mediated specific killing effect. a, b) Percentage of dead target (LLC) cells were determined by propidium iodide (PI) content using flow cytometry after 6 hours co-incubation with CFSE-labeled LLC cells and collected T cells. The effective T cells/tumor cells ratios (E/T ratio) were 12.5:1, 25:1 and 50:1, respectively. P value was evaluated by one-way ANOVA (*p < 0.05; **p < 0.01; ***p < 0.001; ****p < 0.0001)


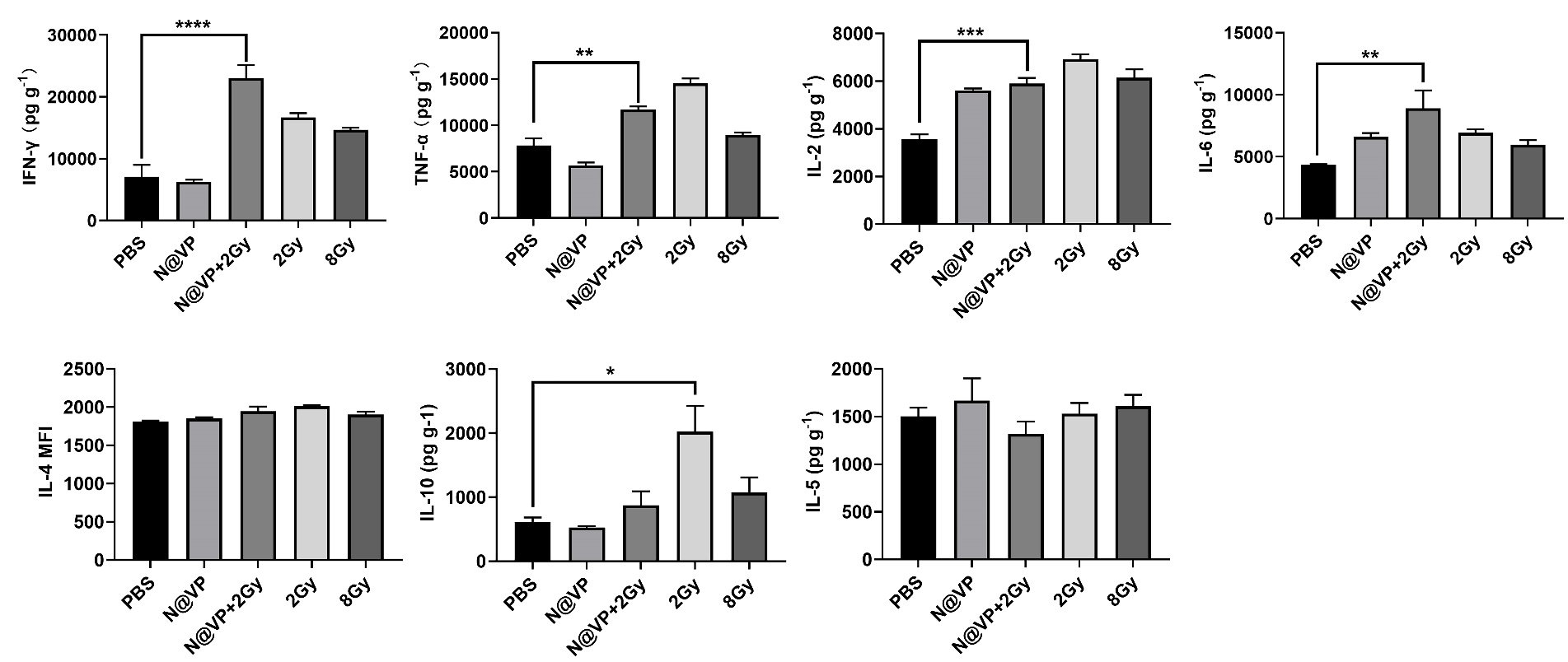


**Figure S27.** Cytokine levels in the LLC tumor tissues from C57BL/6 mice after various treatments. P value was evaluated by one-way ANOVA (*p < 0.05; **p < 0.01; ***p < 0.001; ****p < 0.0001).


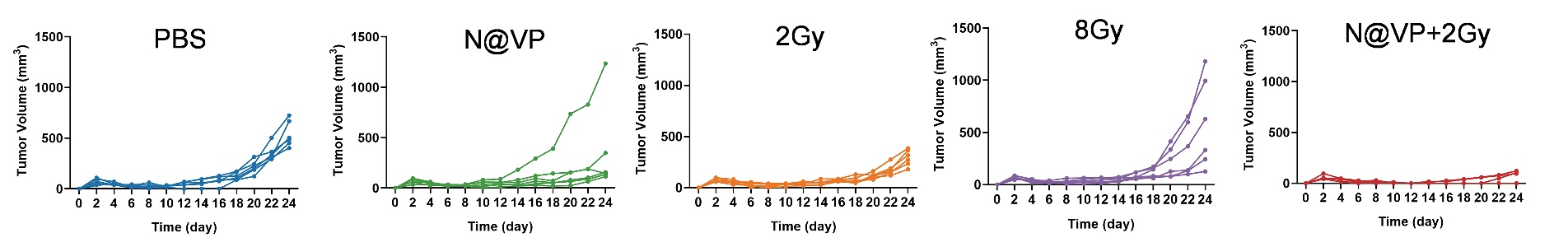


**Figure S28.** Tumor curves of each group (n=6).

**Table S1** List of Abbreviations

| **List of Abbreviations** | |
| --- | --- |
| **Abbreviations** | **Full name or definition** |
| mPEG-PVGLIG-PCL | methoxypolyethyleneglycol-PVGLIG-polycaprolactone |
| N@VP | nanoparticles loading verteporfin |
| Gy | Gray, a radiation dose unit |
| NSCLC | non-small-cell lung cancer |
| ICIs | immune checkpoint inhibitors |
| RT | radiation therapy |
| TAAs | tumor-associated antigens |
| TME | tumor microenvironment |
| 8Gy × 3f | three 8 Gy fractions |
| HDRT | high-dose radiotherapy |
| SBRT | stereotactic body radiation therapy |
| VP | verteporfin |
| PDT | photodynamic therapy |
| ROS | reactive oxygen species |
| ^1^O_2_ | singlet oxygen |
| MMP | matrix metalloproteinase |
| DSBs | double-strand breaks |
| IR | ionizing radiation |
| X-PDT | X-ray-induced photodynamic therapy |
| DAMPs | damage-associated molecular pattern molecules |
| APCs | antigen presenting cells |
| GSDMD | gasdermin D |
| CDRT | conventional-dose radiotherapy |
| SOSG | singlet oxygen sensor green |
| MFI | mean fluorescence intensity |
| MALDI-TOF-MS | matrix-assisted laser desorption/ionization-time-of-flight-mass spectra |
| ^1^H NMR | proton nuclear magnetic resonance |
| PDI | polydispersity index |
| TEM | transmission electron microscope |
| HPLC | high performance liquid chromatography |
| ECM | extracellular matrix |
| LLC | lewis lung carcinoma |
| PBS | phosphate-buffered saline |
| NIR | near-infrared fluorescence |
| BNP | blank nanoparticles |
| N@DiR | DiR-loaded nanoparticles |
| DCFH-DA | dichlorodihydrofluorescein diacetate |
| H&E | hematoxylin-eosin |
| HUVEC | human umbilical vein endothelial cells |
| Th cells | T help cells |
| Treg cells | regulatory T cells |
| TNF-α | tumor necrosis factor alpha |
| IL | interleukin |
| IFN-γ | interferon-gamma |
| CTLs | cytotoxic T lymphocytes |
| HMGB1 | high mobility group box 1 |
| ATP | adenosine triphosphate |
| DAMPs | damage-associated molecular pattern molecules |
| ICD | immunogenic cell death |
| DC | dendritic cells |
| MHC | major histocompatibility complex |
| TIME | tumor immune microenvironment |
| PRRs | pattern recognition receptors |
| DEGs | differentially expressed genes |
| RNA-seq | RNA sequencing |
| GSEA | gene set enrichment analysis |
| KEGG | Kyoto Encyclopedia of Genes and Genomes |
| NLRP3 | domains-containing protein 3 |
| Caspase1, Casp1 | cysteine aspartic acid-specific protease 1 |
| GSDMD-FL | full length gasdermin D |
| GSDMD-N | cleaved GSDMD |
| PD-1 | programmed death-1 |
| PD-L1 | programmed death-ligand 1 |
| αP | anti-PD-1 antibody |
| Con | control |
| E/T ratio | effective T cells/tumor cells ratio |
| Tem | effector memory T cell |
| Tcm | central memory T cell |
| mPEG-NHS | Methoxypolyethyleneglycol-NHS |
| PCL-NH2 | polycaprolactone-NH2 |
| PVGLIG | Pro-Val-Gly-Leu-Ile-Gly, MMP2/MMP9 responsive peptide |
| RPMI | roswell park memorial institute |
| FBS | fetal bovine serum |
| MeV | million electron volts |
| DMF | dimethyl formamide |
| MWCO | molecular weight cut-off |
| DMAP | 4-dimethylaminopyridine |
| EDC | 1-ethyl-3-(3-dimethyl aminopropyl) carbodiimide |
| NHS | N-Hydroxysuccinimide |
| DCM | methylene chloride |
| PVA | polyvinyl alcohol |
| DLS | dynamic light scattering |
| AST | aspartate transaminase |
| ALT | alanine transaminase |
| LDH | lactate dehydrogenase |
| CREA | creatinine |
| CK | creatine kinase |
| CK-MB | creatine kinase isoenzymes |
| α-HBDH | α-hydroxybutyrate dehydrogenase |
